# New Australian stiletto flies: revision of *Manestella* Metz and description of *Medomega* gen. n. (Diptera, Therevidae, Agapophytinae)

**DOI:** 10.3897/zookeys.240.2967

**Published:** 2012-11-09

**Authors:** Shaun L. Winterton, Christine L. Lambkin

**Affiliations:** 1 California State Collection of Arthropods, California Department of Food & Agriculture, Sacramento, California, USA; 2 Entomology, Queensland Museum, South Brisbane, Queensland, Australia

**Keywords:** Asiloidea, natural language description, cybertaxonomy, Lucid Builder

## Abstract

The previously monotypic genus *Manestella* Metz, 2003 is revised with a single species, *Manestella tristriata* (Mann, 1933), redescribed and an additional 14 new species described: *Manestella caesia* **sp. n.**, *Manestella campestris* **sp. n.**, *Manestella canities* **sp. n.**, *Manestella cooloola* **sp. n.**, *Manestella fumosa* **sp. n.**, *Manestella incompleta* **sp. n.**, *Manestella nubis* **sp. n.**, *Manestella obscura* **sp. n.**, *Manestella ocellaris* **sp. n.**, *Manestella persona* **sp. n.**, *Manestella poecilothorax* **sp. n.**, *Manestella umbrapennis* **sp. n.**, *Manestella vasta* **sp. n.** and *Manestella vespera* **sp. n.** The putative sister genus to *Manestella*, *Medomega* **gen. n.**, is described containing six new species: *Medomega averyi* **sp. n.**, *Medomega bailmeup* **sp. n.**, *Medomega chlamydos* **sp. n.**, *Medomega danielsi* **sp. n.**, *Medomega gigasathe* **sp. n.**, and *Medomega nebrias* **sp. n.** Complete taxonomic descriptions were generated from a character matrix developed in Lucid Builder from which natural language descriptions (NLD) were parsed. Images of all species of *Manestella* and *Medomega* **gen. n.** are included, along with dichotomous keys to species.

## Introduction

Australasia is the most species-rich biogeographical region for stiletto flies (Diptera: Therevidae), comprising 375 described species in 26 genera exclusively placed in two subfamilies, Agapophytinae (209 spp. in 23 gen.) and Therevinae (166 spp. in 3 gen.) (Winterton 2009, 2011). All genera but one (i.e. *Irwiniella* Lyneborg, 1976) are endemic to the region. There are also a significant number of new species and several genera in collections remaining to be described, with the fauna expected to total at least twice this number when fully documented.

In their revision of the genus *Psilocephala* Zetterstedt, 1838, Metz et al. (2003) erected *Manestella* Metz, 2003 as a monotypic genus to accommodate *Psilocephala tristriata* Mann (1933). Since then, more undescribed species have been identified in collections, particularly from Western Australia where the genus is relatively species rich. Fourteen new species of *Manestella* are described herein: *Manestella caesia* sp. n., *Manestella campestris* sp. n., *Manestella canities* sp. n., *Manestella cooloola* sp. n., *Manestella fumosa* sp. n., *Manestella incompleta* sp. n., *Manestella nubis* sp. n., *Manestella obscura* sp. n., *Manestella ocellaris* sp. n., *Manestella persona* sp. n., *Manestella poecilothorax* sp. n., *Manestella umbrapennis* sp. n., *Manestella vasta* sp. n. and *Manestella vespera* sp. n. All species are endemic to Australia and are commonly found in coastal heath habitats. *Manestella* includes some of the smallest sized stiletto flies, with body length rarely exceeding 5.0 mm.

A new endemic Australian genus is described and is the putative sister genus to *Manestella*. This close relationship is based on characters in the male genitalia such as the apodemes of the parameral sheath joining midway along distiphallus rather than proximal to the basiphallus (Figs 6E, J, 72E, J); numerous strong setae commonly on the head, thorax, and apices of the gonostylus and inner gonocoxal process, as well as a characteristic glaucous pubescence overlying much of the body. *Medomega* gen. n. contains six new species: *Medomega averyi* sp. n., *Medomega bailmeup* sp. n., *Medomega chlamydos* sp. n., *Medomega danielsi* sp. n., *Medomega gigasathe* sp. n. and *Medomega nebrias* sp. n.

*Manestella* was previously placed in the poorly defined *Taenogera* genus-group (Winterton et al. 1999b; Metz et al. 2003), but along with *Medomega* gen. n., is now contained within a more inclusive Agapophytinae (Winterton 2006, 2011) based on the following characteristics: femoral pile uniformly short and erect and lacking secondary appressed pile; presence of three spermathecae connected to the spermathecal sac duct (Winterton et al. 1999c), female acanthophorite spines A1 and A2 well developed; male ventral apodeme of parameral sheath forked and gonocoxites often with a ventral velutum patch (Winterton et al. 2001). While *Medomega* and *Manestella* are putative sister genera, the relationship of these genera to other Agapophytinae genera is unclear. The presence of velutum on the gonocoxites of some species in both genera, but complete lack of femoral velutum patches in all species, suggests a possible sister relationship with the clade comprising *Bonjeania* Irwin & Lyneborg, 1989, *Acatopygia* Kröber, 1912, *Agapophytus* Guérin, 1831, *Acupalpa*, Kröber, 1914, etc. (i.e. all genera originally placed by Winterton et al. (2001) in Agapophytinae *sensu stricto*). *Manestella* can be differentiated from all other agapophytine genera based on the following characteristics: wing cell m_3_ open, subapical *av* seta on hind femur absent, femora without elongate velutum patches, male usually with multiple rows of postocular setae, and abundant bristle-like setae and glaucous pubescence on the entire body in both sexes (more pronounced in males). *Medomega* gen. n. is differentiated from all other agapophytine genera by the following characteristics: head much higher than long, ocellar tubercle raised; wing vein R_2+3_ reflexed anteriorly with a kink, cell m_3_ open; femoral velutum patches absent and hind femur with one or more subapical *av* setae. Herein we revise *Manestella* and describe *Medomega* gen. n. as new. *Manestella tristriata* (Mann) is redescribed with descriptions of 14 new species of *Manestella* and six new species of *Medomega* gen. n. Keys to species are provided for both genera.

## Materials and methods

Adult morphological terminology follows McAlpine (1981) with genitalic morphology as modified by Winterton et al. (1999a,b) and Winterton (2006). Genitalia were macerated in 10% KOH to remove soft tissue, then rinsed in distilled water and dilute glacial acetic acid, and dissected in 80% ethanol. Female reproductive organs were stained with a saturated solution of Chlorazol Black in 40% ethanol. Genitalia preparations were placed in glycerine in a genitalia vial mounted on the pin beneath the specimen.

Types are deposited in the following institutions and collections: Australian Museum (Sydney) (AMS), Australian National Insect Collection (Canberra) (ANIC), Queensland Museum (Brisbane) (QM), Western Australian Museum (Perth) (WAM), Greg Daniels private collection [to be ultimately housed in the Australian Museum] (GDCB/AMS), California Academy of Sciences (San Francisco) (CAS), California State Collection of Arthropods (Sacramento) (CSCA). Numbers quoted with individual specimens as MEI000000 are unique identifiers in the therevid database MANDALA and are attached to each specimen as a yellow or white label (Kampmeier and Irwin 2009). Material examined lists were exported from MANDALA. Descriptions were constructed using Lucid Builder 3.5, using a matrix database of character states, which were then exported using the natural language function into XML and a text document. Specimen images were taken at different focal points using a digital camera and subsequently combined into a serial montage image using Helicon Focus software. All new nomenclatural acts and literature were registered in Zoobank (Pyle and Michel 2008).

## Taxonomy

### Manestella

Metz

http://species-id.net/wiki/Manestella

Manestella Metz, 2003: 10. Type species *Psilocephala tristriata* Mann, 1933: 331, by original designation.

#### Diagnosis. 

Body length rarely exceeding 5.0 mm. Body usually covered with dense glaucous grey pubescence with darker brown or grey markings on head and scutum, admixed with numerous, dark setae; head length approximately equal to or slightly longer than height; male frons narrow with eyes often contiguous; parafacial pile absent, pubescence with silver velutum band laterally between antennal base and eye; face concave, grey pubescent; male with one or more rows of postocular setae; antennae shorter than or equal to head length; scutal pubescence glaucous, marked with dark brown, markings frequently as two medial stripes anteriorly, fused posteriorly, laterally stripes broken or irregularly tessellated; prosternal depression without setae; metanepisternum with post-spiracular setae absent; setae absent on posterior surface of mid coxa; femoral velutum patches absent; hind femur without subapical *av* setae; fore femur without macrosetae; hind coxal knob present; wing markings typically brown infuscate and white translucent, mottled or banded, sometimes uniform infuscate, often with additional spur veins and/or extra crossveins; vein M_3_ sometimes incomplete; cell m_3_ open; abdominal tergite 2 usually with patch of short setae medially; gonocoxites with diffuse velutum patch ventrally (sometimes absent); inner gonocoxal process present; dorsal and ventral apodemes of parameral sheath joining along distiphallus, both forked; female with three spermathecae; small rounded spermathecal sac present; spermathecal ducts joining to common spermathecal sac duct; acanthophorite spines A1 and A2 present, well developed.

#### Comments.

*Manestella* contains some of the smallest stiletto flies, with a typical body length range of 3.5–5.0 mm. Females of the largest species attain a mere 5.5 mm total body length. External and male genitalic morphology are relatively conserved in this genus, and body colouration is generally grey and brown pubescent with mottled scutum and wings. The male abdomen often has a silver velutum covering. Atypical for therevids, there is considerable variation in wing venation in species in this genus, with individuals sometimes showing different arrangements of veins in each wing (e.g. spur veins or incomplete M_3_). *Manestella* is closely related to *Medomega* gen. n. based on characters such as the apodemes of the parameral sheath joining midway along the distiphallus rather than proximal to the basiphallus; numerous strong setae commonly on the head, thorax, and apices of the gonostylus and inner gonocoxal process, as well as a characteristic glaucous pubescence overlying much of the body. *Manestella* can be identified using the dichotomous key to Australasian genera in Winterton (2011).

#### Included species.

*Manestella caesia* sp. n.; *Manestella campestris* sp. n.; *Manestella canities* sp. n.; *Manestella cooloola* sp. n.; *Manestella fumosa* sp. n.; *Manestella incompleta* sp. n.; *Manestella nubis* sp. n.; *Manestella obscura* sp. n.; *Manestella ocellaris* sp. n.; *Manestella persona* sp. n.; *Manestella poecilothorax* sp. n.; *Manestella tristriata* (Mann); *Manestella umbrapennis* sp. n.; *Manestella vasta* sp. n.; *Manestella vespera* sp. n.

#### Key to *Manestella* species

Most key couplets rely heavily on male characteristics as females are difficult to distinguish for many species. External characters are used where possible throughout the key, but male genitalic dissections should be examined to confirm identity. Unassociated females cannot be confidently separated for most species of *Manestella* except for *Manestella poecilothorax* sp. n.; females are unknown for *Manestella persona* sp. n., *Manestella nubis* sp. n., *Manestella umbrapennis* sp. n. and *Manestella vasta* sp. n.

**Table:** 

1	Wing uniformly dark smoky infuscate (e.g. Figs 13, 26, 35, 39, 56, 59, 65)	2
–	Wing with dark to suffuse mottling tending to more extensive infuscation with white translucent fenestrations (e.g. Figs 20–24, 31, 43, 46, 47, 54), in some species largely translucent with several irregular markings and darkening along wing veins (e.g. Figs 7, 9-10, 48)	8
2(1)	Frons with numerous elongate setae immediately above antennae (Western Australia)	3
–	Frontal setae minute or absent; (Figs 13, 38) (most states)	4
3(2)	Male frontal setae divided medially to form two patches (Fig. 57); outer gonocoxal process less than half the length of inner gonocoxal process (Fig. 58A)	*Manestella umbrapennis* sp. n.
–	Male frontal setae a single patch only, not divided medially (Fig. 62); outer gonocoxal process at least half the length of inner gonocoxal process (Fig. 67A)	*Manestella vespera* sp. n.
4(2)	Abdominal segments 1–2 suffused with dark yellow laterally (Fig. 12); male frons without setae (Fig. 13) (South Australia)	*Manestella campestris* sp. n.
–	Abdomen uniform dark grey-brown (often overlain with grey pubescence); male frons usually with setae, but sometimes reduced or absent (Fig. 27)	5
5(4)	Male frons without setae; outer gonocoxal process relatively short, abruptly narrowed apically (Fig. 41) (Queensland)	*Manestella obscura* sp. n.
–	Male frons with small to minute setae present; outer gonocoxal process relatively elongate, spatulate (Figs 29, 60)	6
6(5)	Male with single row of postocular setae immediately laterad of ocellar tubercle (more irregularly arranged setae laterally) (Figs 35, 36); distiphallus relatively elongate, extending ventrally beyond gonocoxite (Queensland)	*Manestella nubis* sp. n.
–	Male with two well defined rows of postocular setae; distiphallus relatively short, not extending ventrally beyond gonocoxite (Figs 29, 60)	7
7(6)	Male gonocoxites without triangular medially directed process; inner gonocoxal process dark sclerotized, ladle-shaped with numerous strong setae apically; ejaculatory apodeme relatively narrow (Fig. 60) (Western Australia)	*Manestella vasta* sp. n.
–	Male gonocoxites with triangular medially directed process; inner gonocoxal process lightly sclerotized, narrow with few strong setae apically, ejaculatory apodeme relatively broad (Figs 6F-J, 29) (Western Australia)	*Manestella fumosa* sp. n.
8(1)	Femora yellow basally (Figs 1–2); wing mostly translucent, small markings present (darker in female); wing venation yellowish towards base of wing (Figs 48–49); gonocoxite with shorter and darker setae ventrally (Fig. 50) (Western Australia)	*Manestella poecilothorax* sp. n.
–	Femora uniformly dark (rarely with yellowish suffusion); wing variable, but usually with extensive dark banding or mottling, venation dark throughout, rarely yellowish at wing base; gonocoxite setae uniform in colour and length	9
9(8)	Scutellum with two pairs of large macrosetae (Figs 3, 47) (Western Australia)	*Manestella persona* sp. n.
–	Scutellum with one pair of large macrosetae (smaller setae occasionally present laterad)	10
10(9)	Male frontal setae relatively short, distinctly weaker than setae on scape	13
–	Male frontal setae relatively long and robust, approximately equal in length and thickness to setae on scape (Figs 17, 42)	11
11(10)	Male with single row of postocular setae immediately laterad of ocellar tubercle; male abdomen brown pubescent dorsally, uniform grey pubescent laterally (Figs 42–43); gonocoxites with glossy, glabrous area posteromedially (Queensland)	*Manestella ocellaris* sp. n.
–	Male with two rows of postocular setae (irregular) immediately laterad of ocellar tubercle; male abdomen completely covered with silver grey pubescence (Fig. 18–19), or with silver grey pubescence posterolaterally on abdominal tergites 1–5 (Fig. 30); gonocoxites with uniform velutum ventrally, without distinctive glabrous area	12
12(11)	Wing vein M_3_ complete, joining to wing margin; male abdomen covered with uniform silver-grey velutinous pubescence (Fig. 17) (Western Australia)	*Manestella canities* sp. n.
–	Wing M_3_ frequently incomplete, terminating before wing margin (Fig. 30); male abdomen only with silver-grey pubescence laterally (Figs 30, 32) (Western Australia)	*Manestella incompleta* sp. n.
13(10)	Gonocoxites with dark medially directed sclerotized process immediately ventral of ventral lobe (*cf*. Fig. 6H) (Fig. 25) (Queensland)	*Manestella cooloola* sp. n.
–	Gonocoxites without dark sclerotized process ventral of ventral lobe (Figs 11, 55)	14
14(13)	Male wing mostly white translucent (Figs 7, 8); frons relatively flat, not protruding (Fig. 8) (southern Australia)	*Manestella caesia* sp. n.
–	Male wing more extensively marked (Figs 51, 52); frons protruding anteriorly (eastern Australia)	*Manestella tristriata* (Mann, 1933)

### Manestella caesia sp. n.

urn:lsid:zoobank.org:act:FF995D63-CD6E-4591-BD44-6E989E2DAFAB

http://species-id.net/wiki/Manestella_caesia

Figs 5A 7 8 9 10 11

#### Type material.

**Holotype** male, AUSTRALIA: **Western Australia:** 27.4 km N Payne’s Find, [-29.25, 117.667], 400m, 3.x.1962, E. S. Ross, D. Q. Cavagnaro (MEI_025419, CAS).

**Paratypes**. AUSTRALIA: **Western Australia**: male, Badgingarra National Park, 40 km E Cervantes, [-30.5, 115.067], 30.x.1987, M. E. Irwin, E. I. Schlinger (MEI_088349, CAS); male, 2 females, Lesueur NP: Cockleshell Gully: 20 Sep-9 Nov 2003 C Lambkin N Starick J Recsei Eucalyptus woodland: Malaise 59 m: ANIC Bulk Sample 2175 30°08'47"S, 115°06'27"E (GPS) (ANIC_29:018313, 018314, 018315, ANIC); 4 males, 15 km N Wanneroo, [-31.75, 115.833], 24.x.1987, M. E. Irwin; sand hill with low heath (MEI_022886, 022887, 022889, 022890, WAM). **South Australia:** male, 18 km SSW Pinnaroo, [-35.417, 140.817], 20–24.x.1983, ex. ethanol, I. D. Naumann, J. C. Cardale (MEI_022888, ANIC).

#### Other material examined.

AUSTRALIA: **Western Australia:** 3 males, 2 females, Lesueur NP: Cockleshell Gully: 20 Sep-9 Nov 2003 C. Lambkin, N. Starick, J. Recsei, Eucalyptus woodland: Malaise 59 m: ANIC Bulk Sample 2175, 30°08'47"S, 115°06'27"E (GPS) (ANIC_29: 018316, 018317, 018318, 018319, 018326, ANIC); **Victoria**: male, 5 females, Wyperfeld National Park, Murrayville Track, 45.2 km SSE Murrayville, 14–20.xi.2002, C. Lambkin, D. Yeates, N. Starick, J. Recsei, 35°39'26"S, 141°19'30"E [-35.657, 141.325] (ANIC_29:009093, 009094, 009095, 009096, 009100, 009104, ANIC).

#### Diagnosis.

Frontal setae smaller than setae on scape (in both sexes); male postocular setae in single row with irregular setae dorsomedially; male wing mostly white translucent, with brown infuscate markings apically on cells bm, br and d, female wing darker with markings along most wing veins; femora brown, yellow apically; male abdomen with velutum; triangular ventromedial process on gonocoxites absent.

#### Description.

Body length= 3.5–4.0 mm (male), 3.5–4.5 mm (female). *Head*. Frontal pubescence silver-grey with dark brown markings, profile flat, lower frontal markings as narrow brown stripe (male), or brown quadrangle dorsally, brown band above antennae (female); frontal setae dark; male frontal vestiture with patch of short setae above antenna, shorter than setae on scape, female frontal vestiture with short to moderate length setae; male frons width at narrowest point narrower than anterior ocellus but eyes not contiguous; male postocular setae black, as a single row, additional setae irregularly arranged medially, female with two regular rows; occiput pubescence grey, narrow triangular marking medially (female); genal setae white, elongate, dense and curved anteriorly; antennal scape shorter than flagellum, vestiture as numerous large dark setae (admixed with shorter setae); flagellum brown. *Thorax*. Scutum pubescence grey with brown markings, vestiture as scattered short dark setae, longer and paler posteriorly in male; scutal markings as two dark medial stripes anteriorly, joining posteriorly, stripes broken to tessellate laterally; scutal macrosetae dark; pleuron with silver-grey pubescence; katatergite setae uniformly pale; coxae dark, overlain with silver-grey pubescence; coxal setae mostly pale; femora dark grey-brown, apices yellow, vestiture as extensive long pale setae (male) or short dark setae, admixed with longer pale setae (female); tibiae yellow, dark grey-brown apically; tarsi dark brown, basal portions of tarsomeres 1–2 yellow; wing white translucent, infuscate along wing veins and apically in cells cu-p, bm and discal (darker in female); venation dark, yellowish basally; scutal chaetotaxy (pairs): notopleural (np) macrosetae 3, supra alar (sa) macrosetae 1, post alar (pa) macrosetae 1, dorsocentral (dc) macrosetae 5–6, scutellar (sc) macrosetae 1. *Abdomen*. Male abdomen base colour brown-black, obscured by extensive velutum, with silver velutum on tergites 2–7, vestiture mostly elongate pale setae, denser laterally; terminalia brown; female abdominal markings with tergites dark brown dorsally (grey pubescence laterally), intersegmental membrane distinctly pale, well defined. *Male genitalia*. Gonocoxite without triangular ventromedial process, velutum extensive, longer posteromedially; outer gonocoxal process relatively elongate, narrowed distally; setae on gonocoxites pale; genitalia dark with grey pubescence, outer gonocoxal process and inner gonocoxal process pale distally.

#### Comments.

*Manestella caesia* sp. n. has a relatively broad distribution throughout the southern mainland states. This species is differentiated form all other *Manestella* by the relatively flat frons with only a few short dark setae, single row of postocular setae and velutinous pubescence on the abdomen in the male.

#### Etymology.

The specific epithet is derived from the Latin *caesius*, bluish-grey; referring to the overall glaucous pubescent body colour.

### Manestella campestris sp. n.

urn:lsid:zoobank.org:act:D3119A1B-0E0F-4447-B07E-66A12AF875F4

http://species-id.net/wiki/Manestella_campestris

Figs 12 13 14 15 16

#### Type material.

**Holotype** male, AUSTRALIA: **Western Australia:** Warren River, 6 mi. SE Pemberton, 16.i.1971, ex. Malaise trap, G. A. Holloway [-34.507, 115.993] (AMS).

**Paratypes.** AUSTRALIA: **Western Australia:** 3 males, same data as holotype [-34.507, 115.993] (AMS); male, 2 females, 24 mi. E Pinjarra 19.i.1971, G. A. Holloway, H. Hughes [-32.556, 116.279] (CAS).

#### Diagnosis.

Frontal setae absent or minute, much smaller than scape setae; frons not protruding; male postocular setae in two rows; male dark yellow to cream laterally on abdominal segments 1–2; wing uniform smoky infuscate; femora brown, male hind femur yellow basally; male abdomen without velutum; gonocoxites without triangular ventromedial processes.

#### Description.

Body length= 3.5–4.0 mm (male), 4.5–5.0 mm (female). *Head*. Frontal pubescence silver-grey with dark brown markings, profile flat, lower frontal markings brown quadrangle dorsally, brown band above antennae; female frontal vestiture with uniform minute setae, setae dark (absent in male); male frons width at narrowest point narrower than anterior ocellus but not contiguous; male postocular setae as two or more rows immediately laterad of ocellar tubercle, setae black; occipital pubescence grey or tan-brown; genal setae pale; antennal scape shorter than flagellum, numerous large dark setae; flagellum brown or brownish orange, darker distally. *Thorax*. Scutal pubescence grey-tan with brown markings, markings as two dark medial stripes anteriorly, joining posteriorly, lateral stripes broken to tessellate (more extensive in female), vestiture as scattered short dark setae (longer in male); scutal macrosetae dark; pleuron with silver-grey pubescence; katatergite setae uniformly pale; anepisternum with grey-brown marking dorsally; coxae dark, overlain with silver-grey pubescence, setae mostly pale; femora dark brown, apices yellow or dark brown, male hind femur yellow basally, vestiture uniform short dark setae; tibiae yellow, dark grey-brown apically; tarsi dark yellow with apices brown; distal segments completely brown; winguniform smoky infuscate; scutal chaetotaxy (pairs): np, 3; sa, 1; pa, 1; dc, 5; sc, 1. *Abdomen*. Male without silver velutum on tergites; base colour dark brown, dark yellow to cream laterally on segments 1–2; vestiture mostly elongate pale setae, denser laterally (darker setae posteriorly); terminalia brown; female abdomen mostly dark brown, anterior segments slightly paler; intersegmental membrane distinctly pale coloured, well delineated. *Male genitalia*. Gonocoxite without triangular ventromedial process, gonocoxite glabrous ventrally, velutum absent; outer gonocoxal process relatively elongate, broad distally; distiphallus slightly spiral-shaped.

#### Comments.

*Manestella campestris* sp. n. is a western species with uniformly smoky infuscate wings, flat frons without macrosetae and gonocoxites without extensive velutum. Abdominal segments 1–2 are slightly lighter in color (more obvious in male) than the rest of the abdomen and this characteristic is unique to this species.

#### Etymology.

The specific epithet is derived from the Latin *campester*, field or plain; referring to the topography of the region that this species was collected.

### Manestella canities sp. n.

urn:lsid:zoobank.org:act:6A5A0E85-5E89-43A7-9B13-3AF774C6CB43

http://species-id.net/wiki/Manestella_canities

Figs 6A–E 17 18 19 20 21

#### Type material.

**Holotype** male, AUSTRALIA: Western Australia, Stirling Range National Park, Stirling Range, Gold Holes [-34.433, 118.067], 20.xi.1987, M. E. Irwin. (MEI_023077, WAM).

#### Paratypes.

8 males, 6 females, same data as holotype (MEI_023070, 023071, 023073, 023076, 023078, 023089, 023092, 023094, 023849, ANIC; MEI_023079, 023080, 023088, 023097, 023099, CAS); 5 males, Stirling Ranges NP, Chester Pass Road: Eucalyptus open woodland 230m, C. Lambkin, J. Recsei, 3–15.xi.2003: Malaise ANIC Bulk Sample 2191, 34°26.033'S, 118°04.386'E (GPS) (ANIC_29:017918, 017919, 017920, 017921, 017922 ANIC).

#### Other material examined.

AUSTRALIA: **Western Australia:** 2 males, female, Stirling Ranges NP, Chester Pass Road: dry creek, sandy soil, 270m, C. Lambkin, J. Recsei, 3–15.xi.2003, Malaise ANIC Bulk Sample 2193, 34°23.684’S, 117°52.962’E (GPS) (ANIC_29:018342, 018343, 018344, ANIC).

#### Diagnosis.

Wing mottled; male frontal and scape setae similar length; male frons protruding slightly; two rows of postocular setae in male; femora black; tibiae dark yellow to brown; male abdomen with silver velutum; ventromedial projection absent on gonocoxites.

#### Description.

Body length= 3.0–3.5 mm (male), 4.0–4.5 mm (female). *Head*. Frontal pubescence silver-grey with dark brown markings, profile with lower frons raised as rounded tubercle around antennal base, lower frontal markings brown medial stripe and spot above antennal base (male) or brown quadrangle medially, brown mark laterally above antennal base (female) (highly variable and irregular in female); male frontal vestiture with patch of short setae above antenna, shorter than setae on scape; female frontal vestiture with more extensive short to moderate length setae (longer dorsally), setae dark; male frons width at narrowest point narrower than anterior ocellus but not contiguous; postocular setae black, in male as two or more rows immediately laterad of ocellar tubercle, or as a single row, with additional setae irregularly arranged medially; occipital pubescence grey, narrow triangular marking medially; genal setae white, elongate, dense and curved anteriorly; antennal scape equal length to flagellum, densely covered with large, dark setae (longer than scape setae); flagellum brown (with grey pubescence). *Thorax*. Scutal pubescence grey with brown markings; vestiture as scattered dark setae, denser anteriorly; scutal markings as two dark medial stripes anteriorly, joining posteriorly, lateral markings irregularly tessellate; macrosetae dark; pleuron with silver-grey pubescence; katatergite setae uniformly pale, rarely with admixed pale and dark setae; coxae dark, overlain with silver-grey pubescence, coxal setae mostly pale; femora dark brown, vestiture as uniform short dark setae (female), or short dark setae, admixed with longer pale setae (male); tibiae yellow, dark grey-brown apically; tarsi dark yellow with apices brown, distal segments completely brown; wingmottled infuscate (as irregular bands); scutal chaetotaxy(pairs): np, 3–4; sa, 1; pa, 1–2; dc, 6–8; sc, 1. *Abdomen*. Male abdomen base colour brown-black, with silver velutum on tergites 2–7, vestiture mostly elongate pale setae, denser laterally; terminalia brown; female abdominal markings with tergites dark brown dorsally, pale grey laterally on anterior segments; female intersegmental membrane distinctly pale, well delineated. *Male genitalia*. Gonocoxite without triangular ventromedial process, gonocoxite with extensive velutum, longer posteriorly; outer gonocoxal process relatively elongate, narrow distally.

#### Comments.

*Manestella canities* sp. n. is known from southwestern Western Australia and is easily diagnosed by the mottled wing, male with protruding frons with dark setae and two rows of postocular macrosetae, and the gonocoxite with extensive velutum but triangular ventromedial process absent.

#### Etymology.

The specific epithet is derived from the Latin *canus*, grey to white; referring to the grey pubescent body colour.

### Manestella cooloola sp. n.

urn:lsid:zoobank.org:act:D4B58E4A-5502-495A-85DE-73D265481F58

http://species-id.net/wiki/Manestella_cooloola

**Figure 4.**
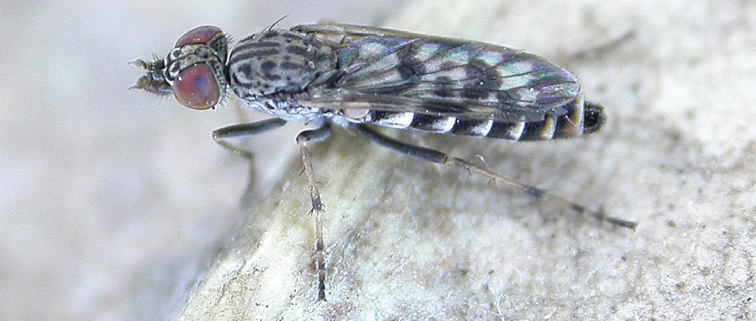
*Manestella cooloola* sp. n., female, Queensland, Great Sandy National Park. Body length = 4.0 mm. Photograph credit: Shaun L. Winterton.

Figs 4 5B 22 23 24 25

#### Type material.

**Holotype** male, AUSTRALIA: **Queensland:** Cooloola Section, Great Sandy National Park [-26.05, 153.06], 6–8.x.2002, J. Skevington (T174963, QM).

**Paratypes.** AUSTRALIA: **Queensland:** 2 males, 1 female, Great Sandy National Park, Cooloola Section, 05.x.1996, S. L. Winterton (CAS) (MEI091005–07); 2 males, 2 females, Great Sandy National Park, Cooloola Section, 01–05.x.1996 [-26.047, 153.075], D. K. Yeates, C. Lambkin, S. L. Winterton (T183043, T183044, T183045, T183046, QM); 1 male, 1 female, Bribie Island, QDPI Fisheries site, heathland-Acacia regrowth [-27.055, 153.193], Malaise trap, S. L. Winterton, N. Power, 12.ix.1997 (CAS).

#### Diagnosis.

Wing mottled; male frontal setae slightly shorter than scape setae; male frons protruding; single row of postocular setae adjacent to ocellar tubercle in male; femora brown; male abdomen with silver velutum; triangular ventromedial projection present on gonocoxites; female frons with concentric brown spot and crescent above antennae.

#### Description.

Body length= 2.5–3.5 mm (male), 4.0–5.0 mm (female). *Head*. Frontal pubescence tan-grey with brown markings, profile with lower frons raised as rounded tubercle around antennal base; lower frontal markings as brown medial stripe and spot above antennal base (male), or brown medial stripe, spot laterally and crescent ventrally (female); frontal setae dark; male frontal vestiture with patch of short setae above antenna, shorter than setae on scape, female frontal vestiture with uniform minute setae; male eyes contiguous above antennae; male postocular setae as single row immediately laterad of ocellar tubercle; postocular setae black; occipital pubescence grey with narrow marking medially; genal setae pale; antennal scape shorter than flagellum, vestiture as numerous large dark setae; flagellum orange-yellow. *Thorax*. Scutal pubescence grey-tan with brown markings, vestiture as scattered short dark setae (female) or scattered short dark setae, longer and paler posteriorly (male), markings as two dark medial stripes anteriorly joining posteriorly, lateral stripes broken to tessellate; scutal macrosetae dark; pleuron with silver-grey pubescence; katatergite setae uniformly pale or with admixed pale and dark setae; anepisternum with grey-brown marking dorsally; coxae dark, overlain with silver-grey pubescence, coxal setae mostly pale; femora dark grey-brown, apices yellow, vestiture as short dark setae, admixed with longer pale setae; tibiae yellow, dark grey-brown apically; tarsi dark yellow with apices brown; wing mottled infuscate (darker in female); scutal chaetotaxy (pairs): np, 3; sa, 1; pa, 1; dc, 3–4; sc, 1. *Abdomen*. Male abdomen base colour brown-black overlain with silver velutum on tergites 2–7, vestiture mostly elongate pale setae, denser laterally; terminalia brown; female abdominal markings with tergites dark brown dorsally, intersegmental membrane distinctly pale, well delineated; grey pubescence posterolaterally on tergites 1–8. *Male genitalia*. Gonocoxite with triangular ventromedial process distinct (rounded apically), gonocoxite velutum extensive, longer medially; outer gonocoxal process relatively short, triangular, narrowed distally; processes on gonocoxite yellow distally.

#### Comments.

*Manestella cooloola* sp. n. is an eastern species found in coastal heath habitats. This species is distinguished by the mottled wing, male gonocoxites with extensive velutum and triangular medial process, and distinctive female frontal pubescence pattern.

#### Etymology.

The specific epithet is named after the Cooloola section of Great Sandy National Park (Queensland), where this species was collected.

### Manestella fumosa sp. n.

urn:lsid:zoobank.org:act:644126B1-9359-4FE9-B3A6-6D17642CF946

http://species-id.net/wiki/Manestella_fumosa

**Figure 6.**
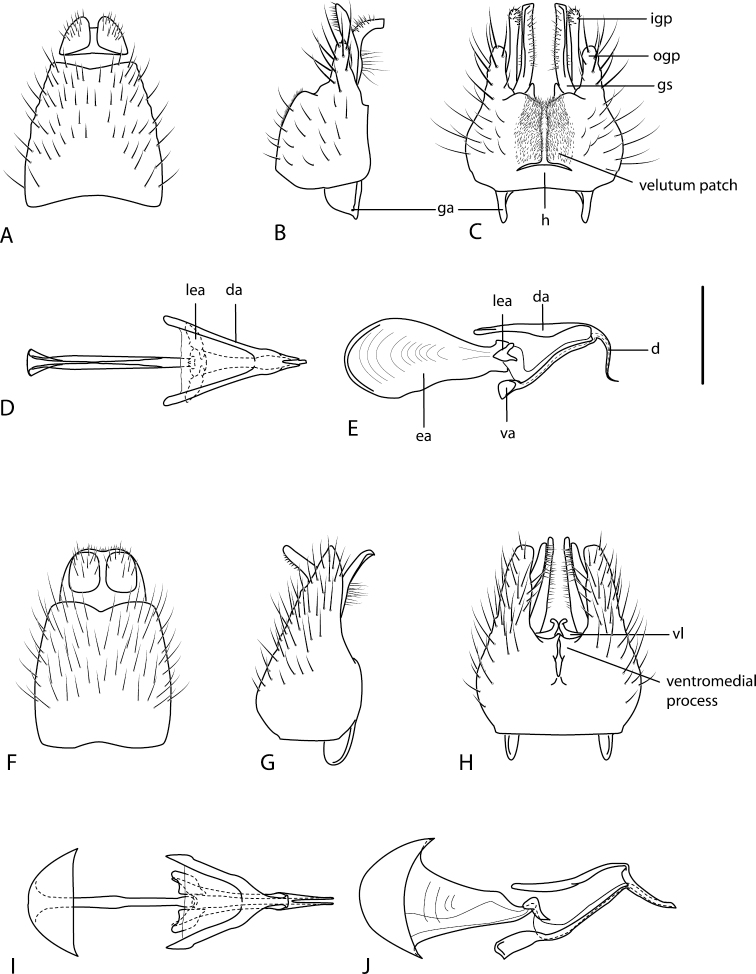
*Manestella* spp., male genitalia. *Manestella canities* sp. n.: **A** epandrium, dorsal **B** gonocoxites, lateral **C** gonocoxites, ventral **D** aedeagus, dorsal **E** aedeagus, lateral. *Manestella fumosa* sp. n.: **F** epandrium, dorsal **G** gonocoxites, lateral **H** gonocoxites, ventral **I** aedeagus, dorsal **J** aedeagus, lateral. Abbreviations: ***d*** distiphallus; ***da*** dorsal apodeme of parameral sheath; ***ea*** ejaculatory apodeme; ***ga*** gonocoxal apodeme; ***ogp*** outer gonocoxal process; ***gs*** gonostylus; ***h*** hypandrium; ***igp*** inner gonocoxal process; ***lea*** lateral ejaculatory apodeme; ***va*** ventral apodeme of parameral sheath; ***vl*** ventral lobe. Scale line = 0.2 mm.

Figs 6F–J 26 27 28 29

#### Type material.

**Holotype** male, AUSTRALIA: **Western Australia:** W of Norseman, *Eucalyptus* woodland, dry gully to salt lake, Malaise trap, C. Lambkin et al., ANIC bulk sample 2184, 1–17.xi.2003 271m [-32.186, 121.721] (WAM).

**Paratypes.** AUSTRALIA: **Western Australia:** 2 males, 2 females, W of Norseman, *Eucalyptus* woodland, dry gully to salt lake, Malaise trap, C. Lambkin et al., ANIC bulk sample 2184, 1–17.xi.2003 271m [-32.186, 121.721] (ANIC, CAS).

#### Diagnosis.

Wing uniform infuscate; male frontal setae minute; male frons flat in profile; two rows of postocular setae adjacent to ocellar tubercle in male; femora brown; male abdomen without silver velutum; acute triangular ventromedial projection present on gonocoxites; gonocoxites without velutum patch; female frontal markings as irregular brown quadrangle dorsally and brown band above antennae.

#### Description.

Body length= 3.0–4.0 mm (male), 4.0 mm (female). *Head*. Frontal pubescence silver-grey with dark brown markings, profile flat, lower frontal markings as small brown spot medially, suffused with light brown above antennae (male), or irregular brown quadrangle dorsally and brown band above antennae (female); male frontal vestiture with patch of short setae above antenna, shorter than setae on scape, female frontal vestiture with short to moderate length setae, frontal setae dark; male frons width at narrowest point narrower than anterior ocellus but not contiguous; male postocular setae as two or more rows immediately laterad of ocellar tubercle (rows well defined); postocular setae black, occipital pubescence grey, triangular marking medially (narrow); genal setae pale; antennal scape shorter than flagellum, scape vestiture as numerous large dark setae; flagellum orange-brown. *Thorax*. Scutal pubescence grey with extensive brown markings, vestiture scattered dark setae, denser anteriorly, scutal markings as two dark medial stripes anteriorly, joining posteriorly, lateral markings broken to tessellate; scutal macrosetae dark; pleuron with silver-grey pubescence; katatergite setae white; anepisternum with brown marking dorsally; coxae dark, overlain with silver-grey pubescence, setae mostly pale; femora dark brown with short dark setae admixed with longer pale setae; tibiae yellow, dark grey-brown apically; tarsi dark yellow with brown apices; terminal segments completely dark; wing uniform smoky infuscate (slightly darker anteriorly); scutal chaetotaxy (pairs): np, 3; sa, 1; pa, 1; dc, 5–6; sc, 1. *Abdomen*. Male abdomen base colour dark brown dorsally, grey pubescent laterally, without silver velutum dorsally, vestiture as short dark setae dorsally, longer pale setae laterally; terminalia brown to dark yellow; female abdominal markings with tergites dark brown dorsally, grey laterally, intersegmental membrane pale coloured, well defined. *Male genitalia*. Gonocoxite with acute triangular ventromedial process; gonocoxite relatively elongate posterolaterally, narrowed distally, velutum patch absent; ejaculatory apodeme greatly enlarged.

#### Comments.

*Manestella fumosa* sp. n. is a distinctive western species with uniform smoky wings, flat male frons with setae reduced, two rows of postocular macrosetae, male gonocoxites with velutum patch absent and triangular ventromedial process present (Figs 6H (arrowed), 29).

#### Etymology.

The specific epithet is derived from the Latin *fumus*, smoke; referring to the dark infuscate wings.

### Manestella incompleta sp. n.

urn:lsid:zoobank.org:act:3F339F86-5192-4140-B75D-6A1E2CD01605

http://species-id.net/wiki/Manestella_incompleta

Figs 30 31 32 33 34

#### Type material.

**Holotype** male, AUSTRALIA: **Western Australia:** Walyunga National Park, [Darling Range], [-31.733, 116.083], 10.xi–16.xi.1987, Malaise trap, M. E. Irwin, E. I. Schlinger. (MEI_023083, WAM).

**Paratypes.** AUSTRALIA: **Western Australia:** 1 male, 4 females, Walyunga National Park, [Darling Range], [-31.733, 116.083], 10.xi–16.xi.1987, 24.xi.1987, Malaise trap, M. E. Irwin, E. I. Schlinger. (MEI_022836, 023104, 023106, 022854, 022855, ANIC); 2 males, 3 females, Kalamunda National Park, Darling Range, Helena River, [-31.95, 116.067], 16.xi.1987, 24.xi.1987, Malaise trap, M. E. Irwin, E. I. Schlinger (MEI_022835, 022837, 022851, 022856, 022858, CAS).

#### Other material examined.

AUSTRALIA: **Western Australia:** 2 females, Charles Darwin Reserve, 12km NE HQ, 29.509°S, 117.05°E, 325m 14–19.ix.2009, Malaise 18313, Jam, *Acacia acuminata*, flowering herbs C. Lambkin, G. Monteith (83249, WAM; T183050, QM); 1 male, same except 19–23.ix.2009, Malaise 18436 (T183051, QM); 2 females, Charles Darwin Reserve, Wanarra Rd, 29.58°S, 116.996°E, 300m 19–23.ix.2009, Malaise 18430, York Gums, *Acacia*, flowering herbs C. Lambkin, G. Monteith (83250, 83251, WAM); 1 male, Karara, 13km SE Boiada Camp, 29.26°S, 116.628°E, 292m 15–24.ix.2009, FIT trap 18352, York Gum/*Acacia* woodland, near clay pan, G. Monteith C. Lambkin (T183047, QM); 1 female, Karara, 16.9 km SE Boiada Camp, 29.256°S, 116.675°E, 312m 15–24.ix.2009, Malaise 18359, *Acacia* woodland, flowering herbs, C. Lambkin G. Monteith (T183048, QM); 1 female, same except 18–24.ix.2009, Malaise 18407 (T183049, QM); 2 females, Lochada, 29.095°S, 116.547°E, 17.ix.2009, Sweep Net on *Calycopeplus paucifolias* 360, R. Leijs (83252, WAM; T183052, QM).

#### Diagnosis.

Wing dark mottled; frontal setae similar length to scape setae; male frons protruding in profile; multiple rows of postocular setae adjacent to ocellar tubercle in male; femora brown; abdomen grey-silver pubescent laterally, brown dorsally; gonocoxites without ventromedial projection; female frontal markings as narrow medial stripe, irregular brown quadrangle dorsally and brown band above antennae.

#### Description.

Body length= 3.5–4.5 mm (male), 4.0–5.0 mm (female). *Head*. Frontal pubescence silver-grey with dark brown markings, lower frons raised as rounded tubercle around antennal base, lower frontal markings brown medial stripe and spot above antennal base (male), or brown quadrangle dorsally, brown band above antennae (female); frontal setae dark, setae similar length to setae on scape (slightly weaker dorsally in female); male frons width at narrowest point narrower than anterior ocellus but not contiguous; male postocular setae as two or more rows immediately laterad of ocellar tubercle; postocular setae black, relatively elongate; occipital pubescence grey, narrow triangular marking medially (postocular setal bases dark); genal setae white, elongate, dense and curved anteriorly; antennal scape equal length to flagellum, densely covered with large, dark setae; flagellum brown. *Thorax*. Scutal pubescence grey-tan with brown markings, numerous elongate dark setae, scutal markings as two dark medial stripes anteriorly, joining posteriorly, lateral stripes broken to tessellate; scutal macrosetae dark; pleuron with silver-grey pubescence; katatergite setae uniformly pale; anepisternum with grey-brown marking dorsally; coxae dark, overlain with silver-grey pubescence, setae mostly pale; femora dark brown with short dark setae admixed with longer pale setae; tibiae yellow, dark grey-brown apically and sometimes also basally; tarsi dark brown with yellow basally on basal segments; wing mottled infuscate, darker and more extensive in male; vein M_3_ often incomplete, ending before wing margin; scutal chaetotaxy (pairs) np, 3; sa, 1; pa, 1; dc, 5–6; sc, 1. *Abdomen*. Base colour dark brown dorsally, grey pubescent laterally; male without extensive silver velutum on tergites, vestiture mostly as elongate pale setae, denser laterally; terminalia brown; female intersegmental membranes distinctly pale, well defined, wider on posterior segments. *Male genitalia*. Gonocoxite without ventromedial process, outer gonocoxal process relatively elongate, narrowed; gonocoxite velutum extensive, distinct; gonocoxite macrosetae dark.

#### Comments.

*Manestella incompleta* sp. n. is a western species distinguished by the mottled wings, male with protruding frons with dark macrosetae, two rows of postocular macrosetae in male, gonocoxites without ventromedial process and velutum patch present, and wing vein M_3_ terminating before wing margin.

#### Etymology.

The specific epithet refers to the incomplete M_3_ vein frequently exhibited by individuals of this species.

### Manestella nubis sp. n.

urn:lsid:zoobank.org:act:B2CD63CE-5324-4103-A6EE-04369628CA50

http://species-id.net/wiki/Manestella_nubis

Figs 35 36

#### Type material.

**Holotype** male, AUSTRALIA: **Queensland:** Carnarvon National Park, Mount Moffatt Section, Headquarters (site 12), [-25.022, 147.954], 740m, 17.xi.1995, D. K. Yeates (MEI_030881, T174964, QM).

#### Diagnosis.

Wing uniform infuscate; male frontal setae minute; male frons flat in profile; single row of postocular setae adjacent to ocellar tubercle in male; femora brown; male abdomen without silver velutum; triangular ventromedial projection absent on gonocoxites, velutum patch absent; distiphallus extending ventrally well beyond gonocoxites.

#### Description.

Body length= 3.5 mm (male). *Head*. Frontal pubescence silver-grey with dark brown markings, frons profile flat, frontal markings as narrow brown medial stripe and suffuse spot above antennal base, patch of short setae above antenna, shorter than setae on scape, setae dark; male frons width at narrowest point narrower than anterior ocellus but not contiguous; male postocular setae as a single row immediately laterad of ocellar tubercle, additional setae irregularly arranged laterally; occipital pubescence grey, narrow brown stripe medially; genal setae white, short and curved anteriorly; antenna scape shorter than flagellum with dark setae, flagellum brown. *Thorax*. Scutal pubescence grey with brown markings, two dark medial stripes anteriorly joining posteriorly, lateral stripes broken to tessellate (pattern diffuse); scattered dark setae, denser anteriorly; katatergite setae uniformly pale; anepisternum uniform grey pubescent; coxae dark, overlain with silver-grey pubescence; coxal setae mostly pale; femora dark grey-brown, apices paler, vestiture short dark setae, admixed with longer pale setae; tibiae yellow, dark grey-brown apically; tarsi dark yellow with brown apices, distal segments completely dark; wing uniform smoky infuscate; scutal chaetotaxy(pairs) np, 3; sa, 1; pa, 1; dc, 4; sc, 1. *Abdomen*. Base colour dark brown; overlain with brown pubescence dorsally, grey pubescent laterally, without silver velutum on tergites, vestiture mostly as elongate pale setae, denser laterally; terminalia brown. *Male genitalia*. Gonocoxite without triangular medial process, outer gonocoxal process relatively elongate, spatulate distally, gonocoxite pubescence barely evident, without velutum patch ventromedially; distiphallus elongate, curved ventrally beyond gonocoxite.

#### Comments.

*Manestella nubis* sp. n. is only known from a single male specimen from Queensland. This species is distinguished by the flat male frons, uniformly infuscate wings, and distiphallus projecting ventrally beyond gonocoxites.

#### Etymology.

The specific epithet is the Latin *nubes*, smoke; referring to the infuscate wings.

### Manestella obscura sp. n.

urn:lsid:zoobank.org:act:3ED49AC6-4F16-436D-AD7B-3CD3BE889CAB

http://species-id.net/wiki/Manestella_obscura

Figs 37 38 39 40 41

#### Type material.

**Holotype** male, AUSTRALIA: **Queensland:** Brisbane Forest Park, Scrub Road, [-27.417, 152.833], 3.x–10.x.1997, Malaise trap, S. Winterton, N. Power, D. White (MEI_091115, T174965, QM).

**Paratypes.** AUSTRALIA: **Queensland:** male, 3 females, Brisbane Forest Park, Scrub Road, [-27.417, 152.833], 12.ix–10.x.1997, Malaise trap, S. Winterton, N. Power, D. White (T183039, T183040, T183041, T183042, QM); male, 2 females, Brisbane Forest Park, Scrub Road, [-27.428, 152.838] Malaise trap, 28.ix–15.x.2002, J. Skevington, J. M. Cumming (CAS).

#### Diagnosis.

Wing uniform infuscate; male frontal setae absent; male frons flat in profile; multiple rows of postocular setae adjacent to ocellar tubercle in male; femora brown; male abdomen with grey pubescence laterally; gonocoxites without ventromedial projection; female frontal markings as diffuse brown quadrangle medially and brown spot above antennae.

#### Description.

Body length= 3.0–4.0 mm (male), 4.0–4.5 mm (female). *Head*. Frontal pubescence grey with brown markings, profile flat, lower frontal markings as brown medial stripe and spot above antennal base (male), or brown quadrangle dorsomedially with diffuse brown band above antennae (female); male frontal vestiture absent, female frontal vestiture as uniform small dark setae, male frons width at narrowest point narrower than anterior ocellus, sometimes contiguous; male postocular setae as two or more irregular rows immediately laterad of ocellar tubercle; occipital pubescence grey, narrow brown stripe medially; genal setae pale; antennal scape shorter than flagellum, numerous dark setae; flagellum brown. *Thorax*. Scutal pubescence grey-tan with brown markings, scattered dark setae, denser anteriorly, scutal markings as two dark medial stripes anteriorly, joined posteriorly, lateral stripes broken to tessellate; pleuron grey pubescent, darker in female with brownish base colour; katatergite setae uniformly pale; coxae dark, overlain with grey pubescence; femora brown with yellow basally and apically, uniform short dark setae, sometimes admixed with longer white setae; tibiae yellow, dark grey-brown apically; tarsi dark yellow with apices brown; wing uniform smoky infuscate; scutal chaetotaxy (pairs): np, 3; sa, 1; pa, 1, dc, 4–5; sc, 1. *Abdomen*. Male abdomen base colour dark brown dorsally, grey pubescent laterally, with sparse silver velutum on tergites 2–7 (laterally), elongate pale setae, denser laterally; terminalia brown; female abdominal tergites dark brown dorsally, intersegmental membrane distinctly pale, well defined. *Male genitalia*. Gonocoxite without ventromedial process, gonocoxite velutum barely evident; outer gonocoxal process relatively short and acuminate; gonocoxite with posterolateral process relatively short, triangular; distiphallus short, straight.

#### Comments.

*Manestella obscura* sp. n. is an eastern species distinguished by the uniformly infuscate wing, male with frons flat and without setae, two rows of postocular macrosetae in both sexes, gonocoxite without a triangular ventromedial process or velutum patch.

#### Etymology.

The specific epithet is derived from the Latin *obscurus*, dark, indistinct; referring to the dark infuscate wings.

### Manestella ocellaris sp. n.

urn:lsid:zoobank.org:act:A92552DE-A172-4994-9CB7-09165F0215AE

http://species-id.net/wiki/Manestella_ocellaris

Figs 42 43 44 45

#### Type material. 

**Holotype** male, AUSTRALIA: **Queensland:** Burrum Heads, 6.ix.1987, G. & A. Daniels [-27.18, 152.6] (AMS).

**Paratypes.** AUSTRALIA: **Queensland:** 6 males, 2 females, Burrum Heads, 6.ix.1987, G. & A. Daniels [-27.18, 152.6] (AMS, CAS).

#### Diagnosis.

Wing mottled infuscate, fenestrations faint in male; male frontal setae similar length to scape setae; frons protruding in profile; single row of postocular macrosetae adjacent to ocellar tubercle in male; femora brown with yellow suffusion; male abdomen without silver velutum; gonocoxites without ventromedial projection, posterolateral area glossy, glabrous; female frontal markings as narrow medial stripe and two diffuse brown spots laterally along eye margin.

#### Description.

Body length= 3.0–4.0 mm (male), 4.0–5.0 mm (female). *Head*. Frontal pubescence silver-grey with light brown markings, lower frontal markings as a narrow brown medial stripe and diffuse spot above antennal base, an additional spot laterally in female; lower frons protruding anteriorly; male frontal vestiture with patch of dark setae above antenna, equal length to setae on scape, female frontal vestiture with uniform small setae, longer immediately above antenna; male frons width at narrowest point less than width of anterior ocellus but not contiguous; male postocular setae as single row immediately laterad of ocellar tubercle, setae black; occiput pubescence grey, narrow brown stripe medially; genal setae pale; antennal scape equal length to flagellum, numerous large dark setae; flagellum dark yellow to brown. *Thorax*. Scutal markings grey with dark brown pattern, two brown pubescence medial stripes anteriorly, joining posteriorly, lateral stripes broken to tessellate, scattered dark setae, denser anteriorly; pleuron with silver-grey pubescence, anepisternum with diffuse brown marking dorsally; katatergite setae uniformly pale; coxae dark, overlain with silver-grey pubescence, setae mostly pale; femora brown with yellowish suffusion (variable intensity), short dark setae admixed with longer pale setae; tibiae yellow, dark grey-brown apically; tarsi dark yellow with apices brown; wing mottled infuscate, fenestrations faint; scutal chaetotaxy (pairs): np, 3; sa, 1; pa, 1; dc, 4–5; sc, 1. *Abdomen*. Abdomen dark brown, grey pubescent laterally; male abdominal vestiture mostly elongate pale setae, denser and more elongate laterally; female intersegmental membrane distinctly pale; terminalia brown. *Male genitalia*. Gonocoxite without triangular medial process, gonocoxite process relatively elongate and spatulate; inner gonocoxal process with strong dark setae apically, gonocoxite velutum extensive, distinct, shiny glabrous area posterolaterally.

#### Comments.

*Manestella ocellaris* sp. n. is an eastern species close to *Manestella cooloola* sp. n. and *Manestella tristriata* based on the mottled wing and protruding male frons with large macrosetae. This species can be distinguished by the lack of velutum on the male abdomen and by the glossy, glabrous patch posteriorly on the gonocoxite.

#### Etymology.

The specific epithet is derived from the Latin *ocellatus*, eyelike spots; referring to the brown markings on the frons.

### Manestella persona sp. n.

urn:lsid:zoobank.org:act:E1C521A6-A74B-43F2-8B3F-0CA02AF111C8

http://species-id.net/wiki/Manestella_persona

Figs 3 46 47

#### Type material.

**Holotype** male, AUSTRALIA: **Western Australia:** Golden Bay, -32.426, 115.771, 19.xi.2008, vegetated dunes, S. L. Winterton, S. D. Gaimari (WAM).

#### Diagnosis.

Wing dark banded infuscate; male frontal setae similar length to scape setae; frons protruding in profile; two rows of postocular macrosetae adjacent to ocellar tubercle in male; head and thoracic setae relatively elongate; scutellum with two pairs of macrosetae; femora dark brown; male abdomen with dense silver velutum; gonocoxites without ventromedial projection, velutum patch present.

#### Description.

Body length= 4.0 mm (male). *Head*. Frontal pubescence silver-grey with dark brown markings, lower frons profile raised around antennal base, frontal markings as brown medial stripe and spot above antennal base; frontal vestiture with small patch of dark, elongate setae above antenna, similar length to scape setae; male frons width at narrowest point narrower than anterior ocellus but not contiguous; male postocular setae as two or more rows immediately laterad of ocellar tubercle, setae black; occipital pubescence grey, triangular marking medially and suffuse brown along postocular ridge; genal setae white, elongate, dense and curved anteriorly; antennal scape equal length to flagellum, scape silver-grey pubescent, densely covered with large, dark setae; flagellum brown, overlain with silver pubescence. *Thorax*. Scutal pubescence grey with brown markings, numerous relatively long, scattered dark setae, denser anteriorly; markings as two dark medial stripes anteriorly joining posteriorly, lateral stripes broken to tessellate; scutal macrosetae dark; pleuron with silver-grey pubescence; katatergite setae uniformly pale; anepisternum with grey-brown marking dorsally; coxae dark, overlain with silver-grey pubescence, setae mostly pale; femora dark brown, overlain with grey pubescence, short dark setae admixed with longer pale setae; tibiae yellow, dark grey-brown apically; tarsi dark yellow with apices brown; wing dark banded infuscate; vein M_3_ incomplete, ending before wing margin; scutal chaetotaxy (pairs): np, 3–4; sa, 1; pa, 1; dc, 6; sc, 2. *Abdomen*. Male abdomen base colour darkish, obscured by extensive silver velutum on tergites 2–7; vestiture mostly elongate pale setae, denser laterally; terminalia brown. *Male genitalia*. Gonocoxite without medial process, gonocoxite posterolateral projection relatively elongate, narrowed distally; gonocoxite velutum extensive, distinct.

#### Comments.

The female of *Manestella persona* sp. n. is unknown. This western species is closely related to *Manestella incompleta* sp. n. based on the wing mottling, male frons and gonocoxite shape and incomplete M_3_ vein. *Manestella persona* sp. n. can be easily distinguished by the two pairs of scutellar macrosetae and longer scutal macrosetae.

#### Etymology.

The specific epithet is derived from the Latin *persona*, mask; referring to the brown markings on the frons.

### Manestella poecilothorax sp. n.

urn:lsid:zoobank.org:act:61068214-A949-4C2F-8099-95C709C9F9D0

http://species-id.net/wiki/Manestella_poecilothorax

Figs 1 2 48 49 50

#### Type material.

**Holotype** male, AUSTRALIA: **Western Australia:** Golden Bay, -32.426, 115.771, 19.xi.2008, vegetated dunes, S. L. Winterton & S. D. Gaimari (WAM).

#### Paratypes.

AUSTRALIA: **Western Australia:** 4 males, 3 females, Nambung National Park, 5 km S Cervantes, [-30.5, 115.067], 4.xi.1987, E. I. Schlinger, M. E. Irwin (MEI_022955, 022957, 022981, WAM; MEI_022959, 022961, 022976, 022978, CAS); 1 male, Kalbarri, [-27.717, 114.167], 30.ix.1973, L. P. Kelsey; sand dunes (MEI_022922, ANIC).

#### Diagnosis.

Wing mostly white translucent with slight infuscation (darker in female); male frontal setae few in number, minute; male frons flat in profile; single row of postocular setae adjacent to ocellar tubercle in male; hind femur yellow with brown patch; male abdomen with grey pubescence; gonocoxites without ventromedial projection; female frontal markings as brown medial stripe, with brown band and spot above antennae.

#### Description.

Body length= 3.0–3.5 mm (male), 4.0–5.0 mm (female). *Head*. Frontal pubescence silver-grey with tan suffusion and brown markings, brown medial stripe and spot above antennal base (male), medial stripe with brown spot dorsolaterally, brown band above antenna (female), frons profile flat in male, slightly rounded in female, male frontal vestiture with small patch of minute setae above antenna, female with short to moderate length setae (longer dorsally), always much shorter than setae on scape; setae on frons and scape dark, admixed with 2–3 white setae laterally; male frons width at narrowest point contiguous, male postocular setae as single row immediately laterad of ocellar tubercle, setae black, with occasional lighter, more elongate seta(e) dorsally; occipital pubescence grey; genal setae pale; antennal scape shorter than flagellum, scape overlain with grey pubescence; flagellum brown with grey pubescence. *Thorax*. Scutal pubescence grey with darker grey-brown pattern; scattered short dark setae, slightly denser anteriorly; scutal markings as two brown medial stripes anteriorly, joining posteriorly, lateral stripes broken to tessellate (darker and more extensive in female); scutal macrosetae dark; pleuron with grey pubescence; katatergite setae uniformly pale; coxae dark, overlain with silver-grey pubescence, setae mostly pale; femora brown with sparse grey pubescence yellow apically and basally, hind femur yellow in basal two thirds, extensive long pale setae; tibiae yellow, dark grey-brown apically; tarsi dark yellow with apices brown; wing mostly white translucent, dark markings apically in cells cu-p, bm and discal, centrally in cell br (darker in female); vein M_3_ incomplete; scutal chaetotaxy (pairs): np, 3; sa, 1; pa, 1; dc, 5–6; sc, 1. *Abdomen*. Male abdomen base colour darkish, overlain with sparse grey pubescence on tergites 2–7, segments 2–3 with cream margin, mostly elongate pale setae, denser laterally; terminalia brown or dark yellow, female abdominal markings with tergites dark brown dorsally, intersegmental membrane distinctly pale, well defined. *Male genitalia*. Gonocoxite without medial process, gonocoxite posterolateral process relatively short, narrowed distally, gonocoxite velutum patch extensive, distinct; patch of shorter, darker setae posteroventrally on gonocoxite.

#### Comments.

*Manestella poecilothorax* sp. n. is a western species with distinctive femoral, scutal and wing colouration. The pale terminalia, incomplete vein M_3_ and flat male frons with minute setae also differentiates this species.

#### Etymology.

The specific epithet is derived from the Greek *poekilos*, spotted; referring to the brown spotted or pied markings on the thorax.

### Manestella tristriata

(Mann)

http://species-id.net/wiki/Manestella_tristriata

Figs 51 52 53 54 55

Psilocephala tristriata Mann, 1933: 331 *–* Irwin and Lyneborg 1989: 358 [catalogue].
Manestella tristriata (Mann) *–*Metz et al. 2003: 13.

#### Type material.

**Holotype** male, AUSTRALIA: **Victoria:** Kiata [-36.363, 141.790], Oct. 1928, F. E. Wilson (NMV).

**‘Allotype’**. AUSTRALIA: **Victoria:** female, Kiata [-36.363, 141.790], Oct. 1928, F. E. Wilson (NMV).

#### Other material examined. 

AUSTRALIA: **Victoria:** 5 males, 5 females, Little Desert National Park, Western Block, Elliots track, 61.5km WSW Nhill [-36.536, 141.028], 19–22.xi.2002, C. Lambkin, D. Yeates, N. Starick, J. Recsei (ANIC).

#### Diagnosis.

Wing mottled infuscate; male frontal setae shorter than scape setae; frons protruding in profile; multiple irregular rows of postocular setae adjacent to ocellar tubercle in male; femora dark brown; male abdomen with dense silver velutum; gonocoxites without ventromedial projection, velutum patch distinct.

#### Redescription.

Body length= 3.0–4.0 mm (male), 4.0–5.0 mm (female). *Head*. Frontal pubescence silver-grey with dark brown markings, profile raised as rounded tubercle around antennal base, narrow brown medial stripe and dark spot above antennal base (male), or brown medial stripe, spot laterally and crescent above antennal base (female); male with patch of short dark setae within dark spot above antenna, shorter than setae on scape, female with scattered short setae, longer dorsally; male frons width at narrowest point narrower than anterior ocellus but not contiguous; male with multiple irregular rows of black postocular setae; occipital pubescence grey, narrow triangular marking medially; genal setae pale; antennal scape almost equal length to flagellum, numerous large dark setae, flagellum brown with silver pubescence laterally and medially. *Thorax*. Scutal pubescence grey with dark brown pattern, scattered dark setae, denser anteriorly, two dark medial stripes anteriorly, joining posteriorly, lateral stripes broken to tessellate; scutal macrosetae long and dark; pleuron with silver-grey pubescence; katatergite setae uniformly pale; anepisternum with grey-brown marking dorsally; coxae dark or pale, overlain with silver-grey pubescence, setae mostly pale; femora dark grey-brown, apices yellow, short dark setae, admixed with longer pale setae; tibiae yellow, dark grey-brown apically; tarsi dark yellow with apices brown; wing mottled infuscate; vein M_3_ complete to wing margin;scutal chaetotaxy (pairs): np, 3; sa, 1; pa, 1; dc, 6; sc, 1. *Abdomen*. Male abdomen base colour darkish, obscured by extensive silver velutum on tergites 2–7, vestiture mostly elongate pale setae, denser laterally; terminalia brown; female abdominal markings with tergites dark brown dorsally, intersegmental membrane distinctly pale, well defined. *Male genitalia*. Gonocoxite without medial process; outer gonocoxal process relatively short, truncated, narrow distally; gonocoxite velutum extensive, distinct with prominent medial tuft.

#### Comments.

*Manestella tristriata* is the type for the genus, and was originally described by Mann (1933) as a species of the broadly defined ‘dump’-genus *Psilocephala* Zetterstedt. Metz et al. (2003) erected *Manestella* based on this species. This species is differentiated from all other *Manestella* species based on the mottled wing, protruding frons, frontal setae smaller than scape setae, and the distinctive tufted velutum patch on the ventral surface of the gonocoxite.

### Manestella umbrapennis sp. n.

urn:lsid:zoobank.org:act:4E5205D2-2FF8-4461-8BCB-B624EA8CCB75

http://species-id.net/wiki/Manestella_umbrapennis

Figs 56 57 58

#### Type material.

**Holotype** male, AUSTRALIA: **Western Australia:** 3 km S Dawesville, at Tim’s Thicket Road, [-32.633, 115.633], 25.x.1987, M. E. Irwin, white sand plain (MEI_022865, WAM).

**Paratypes.** AUSTRALIA: **Western Australia:** 2 males, 3 km S Dawesville, at Tim’s Thicket Road, [-32.633, 115.633], 25.x.1987, M. E. Irwin, white sand plain (MEI_022864, 022866, WAM); 2 males, Kalbarri, Gabba Gabba Gully, [-27.667, 114.167], 17.ix.1981, 19.ix.1981, L. P. Kelsey, on heath (MEI_022869, 022870, ANIC); 3 males, Kalbarri, Shore Road, [-27.717, 114.167], 21.ix.1981, L. P. Kelsey; heath/dunes (MEI_022872, 022874, 022881, ANIC).

#### Diagnosis.

Wing largely uniform infuscate; male frontal setae similar size to scape setae, patch divided medially; male frons protruding in profile; two rows of postocular setae adjacent to ocellar tubercle in male; femora dark brown; male abdomen with brown pubescence, grey laterally; gonocoxites without ventromedial projection or velutum patch.

#### Description.

Body length= 3.0–3.5 mm (male). *Head*. Frontal pubescence silver and dark brown, lower frons protruding around antennal base, markings as brown medial stripe, suffuse brown laterally; frontal vestiture with dense covering of dark, erect elongate setae, narrowly divided medially into two patches above antennae; male frons width at narrowest point narrower than anterior ocellus but not contiguous; two rows of black postocular setae; occipital pubescence grey, narrow triangular marking medially; genal setae white, elongate, dense and curved anteriorly; antennal scape equal length to flagellum, densely covered with large, dark setae; flagellum brown. *Thorax*. Scutal pubescence extensively dark brown with light grey dorsocentral stripes and narrow medial stripe, mottled grey anterolaterally; numerous elongate dark setae, denser anteriorly; macrosetae dark, relatively elongate; pleuron with silver-grey pubescence; katatergite setae uniformly pale; anepisternum with brown marking dorsally; coxae dark, overlain with silver-grey pubescence, setae mostly pale; femora dark brown, short dark setae admixed with longer pale setae; tibiae dark yellow to brown, darker brown apically; tarsi brown, basitarsis dark yellow basally; wing largely uniform smoky infuscate with very faint fenestration centrally; vein M_3_ complete to wing margin; scutal chaetotaxy (pairs): np, 3; sa, 1; pa, 1; dc, 6; sc, 1. *Abdomen*. Male abdomen base colour dark brown, grey pubescent laterally, short dark setae dorsally, longer pale setae laterally; intersegmental membranes distinctly pale; terminalia brown. *Male genitalia*. Gonocoxite without triangular ventromedial process, outer gonocoxal process very short, truncated, gonocoxite velutum patch not present; ejaculatory apodeme narrow.

#### Comments.

The female is unknown for *Manestella umbrapennis* sp. n. This western species is closely related to *Manestella vespera* sp. n. based on the largely uniformly infuscate wing and protruding male frons with large macrosetae, and absence of ventromedial triangular process or velutum patch on the gonocoxite. This species can be distinguished by the male frontal macrosetae patch being divided medially, and by the shape of the aedeagus.

#### Etymology.

The specific epithet is derived from the Latin *umbra*, shadow and *penna*, wing; referring to the brown infuscate wings.

### Manestella vasta sp. n.

urn:lsid:zoobank.org:act:A1A49DCA-E5CD-4889-A91A-C22DADA97382

http://species-id.net/wiki/Manestella_vasta

Figs 59 60

#### Type material.

**Holotype** male, AUSTRALIA: **Western Australia:** 55 km W Paynes Find [-29.263, 117.685], 16.ix.1983, E. I. Schlinger, M. E. Irwin, scrub-desert with annual flowers (MEI_023118, WAM).

**Paratype.** AUSTRALIA: **Western Australia:** male, 30 miles E Merredin [-31.483, 118.286], 375 m, 16.ix.1962, E. S. Ross, D. Q. Cavagnaro (MEI_ 023111, CAS).

#### Diagnosis.

Wing uniform pale infuscate; male frontal setae smaller than scape setae; male frons flat in profile; two rows of postocular setae adjacent to ocellar tubercle in male; femora brown; male abdomen without silver velutum; triangular ventromedial projection absent on gonocoxites; inner gonocoxal process with dense tuft of strong macrosetae.

#### Description.

Body length= 3.0–3.6 mm (male). *Head*. Frontal pubescence silver-grey with dark brown markings, profile flat, narrow brown stripe medially, light brown suffusion above antenna; patch of short black setae above antenna, shorter than setae on scape; male frons width at narrowest point narrower than anterior ocellus but not contiguous; male postocular setae as two rows immediately laterad of ocellar tubercle (rows well defined); postocular setae black, occipital pubescence grey, triangular marking medially (narrow); genal setae pale; antennal scape shorter than flagellum, numerous large dark setae; flagellum brown. *Thorax*. Scutal pubescence grey-tan with brown markings, two dark medial stripes anteriorly, joining posteriorly, lateral stripes broken to tessellate, scattered dark setae, denser anteriorly; scutal macrosetae dark; pleuron with silver-grey pubescence; katatergite setae uniformly pale; anepisternum with grey-brown marking dorsally; coxae dark, overlain with silver-grey pubescence, setae white; femora dark brown with short black setae admixed with longer white setae; tibiae yellow, brown apically; tarsi dark yellow with brown apices; wing uniform smoky infuscate (slightly darker anteriorly); scutal chaetotaxy (pairs): np, 3; sa, 1; pa, 1; dc, 7–8; sc, 1. *Abdomen*. male abdomen base colour dark brown, grey pubescent laterally, without silver velutum dorsally, short dark setae dorsally, longer pale setae laterally; terminalia brown. *Male genitalia*. Gonocoxite without triangular medial process; gonocoxite relatively elongate posterolaterally, narrowed distally; gonocoxite with sparse velutum present ventrally; inner gonocoxal process with dense tuft of strong macrosetae; ejaculatory apodeme narrow.

#### Comments.

The female is unknown for this species. *Manestella vasta* sp. n. can be distinguished by the uniformly infuscate wing, flat male frons with macrosetae present and the inner gonocoxal process having a patch of string macrosetae apically.

#### Etymology.

The specific epithet is derived from the Latin, *vastus*, vast, empty; referring to the desolate habitat of this species.

### Manestella vespera sp. n.

urn:lsid:zoobank.org:act:8C9D32AC-E8C4-492A-9C95-27FF0179AC4F

http://species-id.net/wiki/Manestella_vespera

**Figure 61.**
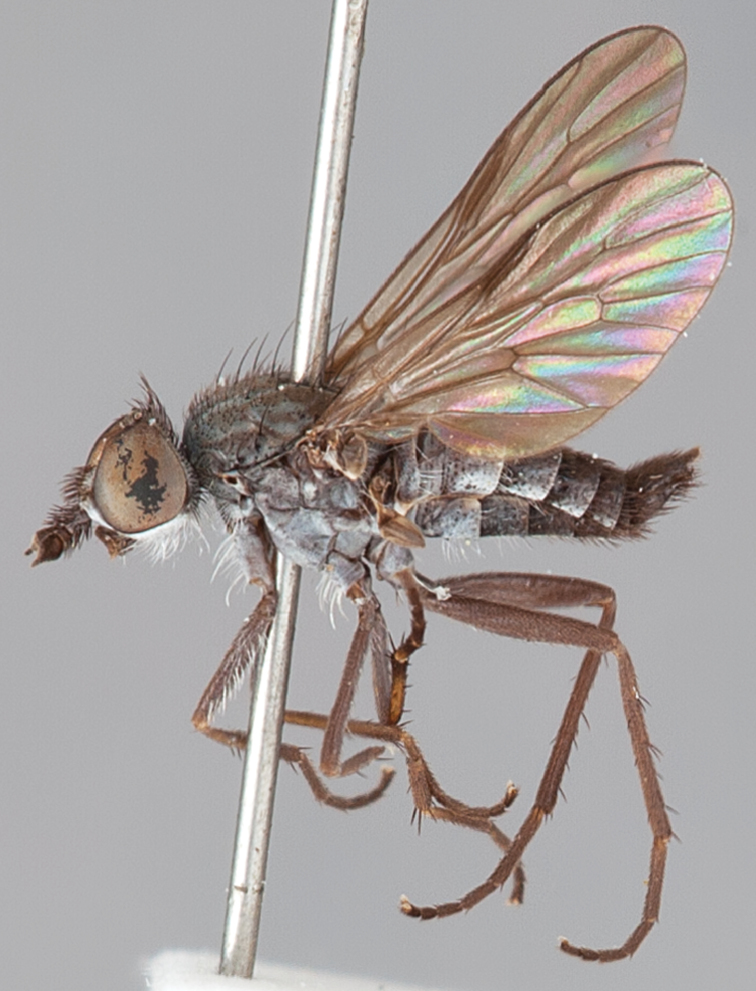
*Manestella vespera* sp. n., male, lateral view. Body length = 3.4 mm.

Figs 61 62 63 64 65 66 67

#### Type material. 

**Holotype** male, AUSTRALIA: **Western Australia:** Walyunga National Park, 40 km NE Perth, [-31.733, 116.083], 26.xi.1987, M. E. Irwin, E. I. Schlinger (MEI_022811, WAM).

**Paratypes.** AUSTRALIA: **Western Australia:** 4 males, 4 females, Walyunga National Park, 40 km NE Perth, [-31.733, 116.083], 26.xi.1987, M. E. Irwin, E. I. Schlinger (MEI_022807, 022810, 022816, 022821, WAM; 022822, 022823, 022833, 022834, CAS); 1 female, Walyunga National Park, 40 km NE Perth, [-32, 115.833], 26–29.x.1987, M. E. Irwin. (MEI_088344, CAS).

#### Diagnosis.

Wing uniform infuscate; male frontal setae similar size to scape setae, patch not divided medially; male frons protruding in profile, rounded; two rows of postocular setae adjacent to ocellar tubercle in male; femora dark brown; male abdomen with brown pubescence, grey laterally; gonocoxites without ventromedial projection; velutum patch reduced; female frontal markings as broad brown quadrangle, silver along eye margin and brown spot above antennae; ejaculatory apodeme enlarged, distiphallus with ventral bulb.

#### Description.

Body length= 3.0–3.5 mm (male), 4.0–5.5 mm (female). *Head*. Frontal pubescence silver with dark brown markings, lower frons protruding above antennal base, rounded, markings as brown medial stripe, suffuse brown laterally (male), or as broad brown quadrangle, silver along eye margin and brown spot above antennae (female); frontal vestiture with dense covering of dark, erect elongate setae in single patch above antennae; male frons width at narrowest point narrower than anterior ocellus but not contiguous; two irregular rows of black postocular setae; occipital pubescence grey, narrow stripe medially; genal setae white, dense and curved anteriorly; antennal scape equal length to flagellum, densely covered with large, dark setae; flagellum brown. *Thorax*. Scutal pubescence extensively dark brown with light grey dorsocentral stripes, grey anterolaterally; numerous relatively elongate dark setae, denser anteriorly; macrosetae dark; pleuron with silver-grey pubescence; katatergite setae uniformly pale; anepisternum without brown marking dorsally; coxae dark, overlain with silver-grey pubescence, setae pale; femora dark brown, short dark setae admixed with longer pale setae; tibiae brown, fore and mid tibiae suffuse with dark yellow basally; tarsi brown, basitarsis dark yellow basally; wing uniform dark smoky infuscate; vein M_3_ complete to wing margin; scutal chaetotaxy (pairs) np, 3; sa, 1; pa, 1; dc, 5–6; sc, 1. *Abdomen*. Male abdomen base colour dark brown, grey pubescent laterally, short dark setae dorsally, longer pale setae laterally; terminalia brown. *Male genitalia*. Gonocoxite without ventromedial process, gonocoxal process relatively long, narrow apically; gonocoxite velutum reduced; ejaculatory apodeme enlarged; distiphallus with ventral bulb.

#### Comments.

*Manestella vespera* sp. n. is a western species closely related to *Manestella umbrapennis* sp. n. This species can be distinguished by the shape of the aedeagus and by the male frontal patch of macrosetae not being divided medially.

#### Etymology.

The specific epithet is derived from the Latin *vesper*, evening, west; referring to both the brown infuscate wings and western distribution of this species.

### Medomega gen. n.

urn:lsid:zoobank.org:act:160AE28E-B03B-4E4A-95A3-256F6F68AAED

http://species-id.net/wiki/Medomega

#### Type species:

*Medomega danielsi* sp. n.

#### Diagnosis.

Body length 6.0–9.0 mm. Body usually covered with dense glaucous grey pubescence; head higher to much higher than long, pronounced ventral projection of head; male frons width variable, ranging from narrower than anterior ocellus to slightly wider than ocellar tubercle, eyes not contiguous; ocellar tubercle raised, black with short, dark setae in both sexes; antennae length variable; scape cylindrical, sometimes bulbous with strong setae, length variable but usually not longer than flagellum; flagellum turbinate with apical style; parafacial pile present but never extensive, rarely absent; face not concave, flat, grey pubescent; palpus 2-segmented, apical segment relatively short; prosternal depression without setae; metanepisternum with post-spiracular setae absent; posterior surface of mid coxa usually bare, sometimes pilose; wing vein R_2+3_ reflexed anteriorly with kink approximately midway; cell m_3_ open (Fig. 71); femoral velutum patches absent; fore femur sometimes with *pd* setae; hind femur with one or more subapical *av* setae; abdominal tergite 2 often with patch of short setae medially; wing infuscate brown and translucent white, mottled to fenestrate; hind coxal knob present; gonocoxite extended posterolaterally; inner gonocoxal process present; dorsal and ventral apodemes of parameral sheath joining along distiphallus, both forked; pubescence sparsely present on gonocoxites; three spermathecae joined to spermathecal sac duct; spermathecal sac present; acanthophorite spines A1 and A2 present, well developed.

#### Comments.

*Medomega* gen. n. is a distinctive genus differentiated from other agapophytine therevids based on the absence of velutum on the femora, cell m_3_ open, head typically higher than long, and mottled wings with vein R_2+3_ reflexed (Fig. 71). In the key to genera by Winterton (2011) *Medomega* gen. n. can be identified by modifying the existing couplets as follows:

**Table:** 

20	Body usually large to medium sized, robust, glossy dark metallic blue or orange; abdomen abruptly tapered; small patch of postspiracular setae present on thorax; wing extensively black (sometimes hyaline basally) or orange infuscate	21.
–	Body size variable, usually relatively slender, never glossy metallic blue or orange; abdomen elongate, evenly tapered; thoracic postspiracular setae absent; wing infuscation variable, usually banded or hyaline, never uniform orange or black	22a.
21	Scape short, setae on antennae and head relatively short; two pairs of scutellar setae; wing with uniform orange infuscation…*Eupsilocephala* Kröber.
–	Scape elongate with numerous enlarged setae; single pair of scutellar setae; wing either with uniform black infuscation or hyaline basally	*Johnmannia* Irwin & Lyneborg.
22a	Body distinctly covered with glaucous grey and brown pubescence; head higher than long; wing usually mottled, wing vein R_2+3_ reflexed anteriorly	*Medomega* gen. n.
–	Body pubescence variable, but usually not densely grey and brown; head not distinctly higher than long, usually longer than high; wing vein R_2+3_ with gentle curve or straight, not with abrupt kink	22.
22	Male and female occiput convex, variously overlain with bronze, matte black, silver and gold pubescence; multiple rows of postocular setae in male; abdomen of equal diameter along length; distiphallus broad, cylindrical; medium to large individuals	*Taenogera* Kröber.
–	Male occiput typically flat to concave, not distinctly convex, rarely overlain with bronze, matte black, silver and gold pubescence; usually single row of postocular setae in male; abdomen tapered; distiphallus usually narrow; size variable	23.

#### Etymology.

The genus name is derived from the Greek, *medos*, counsel, plan and *mega*, very great; referring to the aristocratic shape of the head.

#### Included species.

*Medomega averyi* sp. n.; *Medomega bailmeup* sp. n.; *Medomega chlamydos* sp. n.; *Medomega danielsi* sp. n.; *Medomega gigasathe* sp. n.; *Medomega nebrias* sp. n.;

#### Key to species of *Medomega* gen. n.

**Table:** 

1	Mid coxa with pale setae on posterior surface	2
–	Mid coxa without setae on posterior surface	3
2(1)	Postocular and scutal macrosetae white in male, mostly dark in female; frons without dark spots along eye margin; male abdomen with extensive covering of elongate white setae (Fig. 73); wing mostly white translucent, with slight infuscation along wing veins (Figs 73, 74, 75, 76) (Western Australia)	*Medomega averyi* sp. n.
–	Postocular and scutal macrosetae uniform black in both sexes; large dark spots on frons along to eye margin (Fig. 78); male abdomen without extensive covering of elongate white setae (Fig. 77); wing dark infuscate with white stellate fenestration (Figs 79, 80, 81) (Northern Territory)	*Medomega bailmeup* sp. n.
3(1)	Scutum tan with dark setal bases (Fig. 90); male terminalia very large relative to abdomen length (1/3 total length) (Figs 89, 91, 92) (Western Australia)	*Medomega gigasathe* sp. n.
–	Scutum grey with extensive dark markings not restricted to setal bases (Figs 83, 85, 86); male terminalia rarely unusually large relative to abdomen length	4
4(3)	All setae on head, thorax and abdomen white (Figs 82, 83); abdomen with extensive covering of elongate white setae laterally (Fig. 82); wing mostly white translucent, with slight infuscation along wing veins (Fig. 82, 83) (male only) (Western Australia)	*Medomega chlamydos* sp. n.
–	At least scutal macrosetae dark, usually more extensive dark setae on head (Figs 86, 94); abdomen either with silver velutum pubescence in male only or completely absent, never as white elongate setae; wing extensively infuscate, often as dappled fenestration (Figs 86, 94)	5
5(4)	Scape slightly shorter than flagellum (Fig. 85); male with dense silver pubescence on abdomen, especially laterally; male terminalia orange, relatively large compared with abdomen length (Figs 72F-J, 85) (Queensland)	*Medomega danielsi* sp. n.
–	Scape equal in length to flagellum (Fig. 93); male abdomen with sparse silver velutum covering; male terminalia dark, not large compared to abdomen length (Figs. 93, 94, 98) (New South Wales)	*Medomega nebrias* sp. n.

### Medomega averyi sp. n.

urn:lsid:zoobank.org:act:9851AA0D-0C52-4B9A-8D9B-0F2E31228AB9

http://species-id.net/wiki/Medomega_averyi

**Figure 68.**
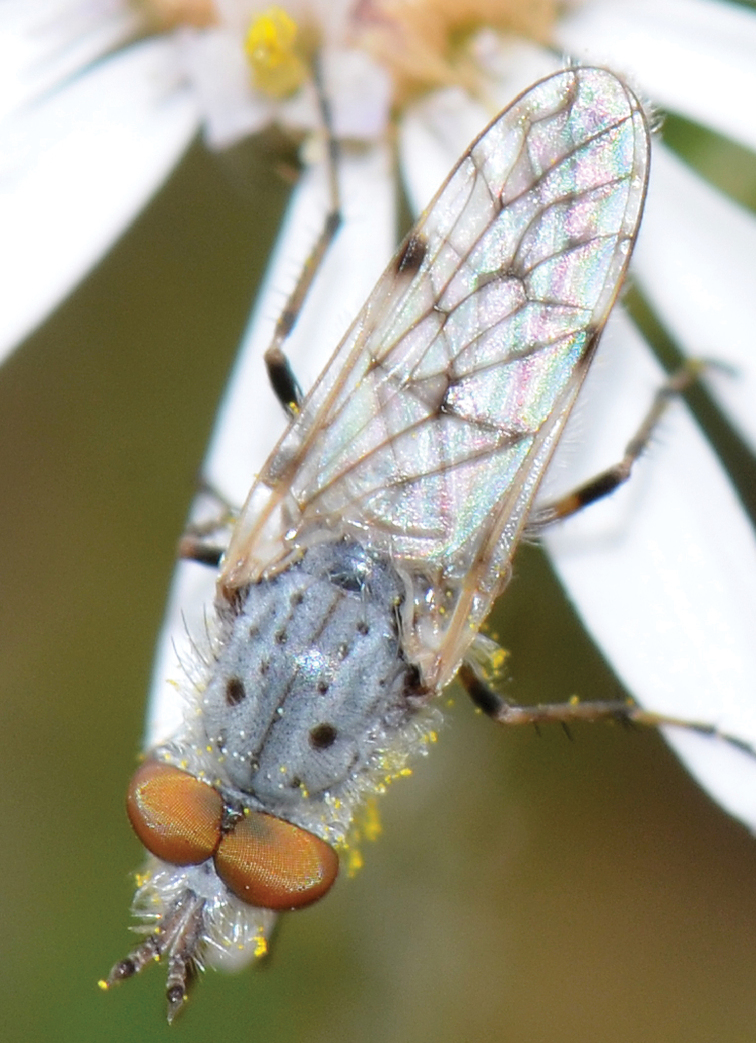
*Medomega averyi* sp. n., male, Western Australia, Stratton. Body length = 7.0 mm. Photograph credit: Fred and Jean Hort

Figs 68 69 70 73 74 75 76

#### Type material. 

**Holotype** male, AUSTRALIA: **Western Australia:** Talbot Road Nature Reserve, O’Connor Road, Stratton [-31.874, 116.044] *Banksia* open woodland, on white sand, feeding on *Oleria pauddentata*, 27.iv.2012, F. Hort, #328 (WAM83222, WAM).

**Paratypes.** AUSTRALIA: **Western Australia:** male, female, same data as holotype (WAM83214, 83215, CAS); 8 females, same data as holotype (WAM83216–83221, 83223–83224, (WAM).

#### Other material examined. 

AUSTRALIA: **Western Australia:** male, Geraldton Dist. [District] Glenfield [-28.692, 114.611], 18.iv.1973, N. McFarland (MEI_032074, BMNH).

#### Diagnosis.

Wing white translucent with irregular brown marginal mottling; most head and body setae white; scutum with narrow medial stripe and spots laterally; scape yellow, brown laterally, longer than flagellum; posterior surface of mid coxa with setae; extensive silver setae on abdomen.

#### Description.

Body length= 5.0–5.7 mm (male), 5.5–7.0 mm (female). *Head*. Male frontal pubescence silver-grey, slightly rounded in profile, patch of fine white setae between antennal base and eye margin; female frons silver-grey laterally, brown-tan medially, frons flat, short dark setae laterally, concentrated above antennal base; male frons width at narrowest point narrower than anterior ocellus, sometimes contiguous; male with single row of white postocular macrosetae immediately laterad of ocellar tubercle, female with scattered dark and pale macrosetae; occipital pubescence grey, narrow marking medially; genal and parafacial setae white, elongate and fine; antennal scape longer than flagellum, orange-yellow, usually with dark suffusion laterally, overlain with sparse grey pubescence admixed with numerous large white setae, several dark setae dorsally; flagellum orange-yellow, distal half dark. *Thorax*. Scutal pubescence silver-grey with narrow brown medial stripe and irregular spots laterally; numerous elongate white setae covering scutum, macrosetae white (male) or black (female), setal bases dark; pleuron with silver-grey pubescence; katatergite setae uniformly white; anepisternum with grey-brown marking; coxae overlain with silver-grey pubescence, setae white, mid coxa with setae on posterior surface; fore femur dark brown-black, mid and hind femora dark yellow, black apically, dense long white setae on anterior and posterior surfaces, hind femur with single *av* setae apically, grey pubescence on all femora; tibiae yellow, black basally and apically; tarsi dark, basitarsus dark yellow basally; wing white translucent, faint mottled infuscation marginally and along wing veins, darker and more extensive in female;scutal chaetotaxy (pairs): np, 3; sa, 1; pa, 1; dc, 3; sc, 1. *Abdomen*. Male abdomen base colour darkish, obscured by dense silver velutum on tergites 2–7, admixed with extensive long erect or semi-appressed silver-white setae; terminalia dark yellow with grey pubescence; female abdomen brown with grey pubescence laterally and posteriorly on segments 1–6; terminalia dark yellow. *Male genitalia*. Epandrium not elongate; gonocoxite with trapezoid shaped outer gonocoxal process.

#### Comments.

*Medomega averyi* sp. n. can be distinguished by the extensive silver-white pile on the head and body, elongate scape, male with white macrosetae on head and thorax, wings mostly white translucent and setae on the posterior surface of the mid coxa. See additional comments under *Medomega chlamydos* sp. n.

#### Etymology.

It is an honour to name this species after the grandfather of the senior author, Avery “Joe” Winterton.

### Medomega bailmeup sp. n.

urn:lsid:zoobank.org:act:66D7D2AE-BE17-4B66-AD21-067DAB2AFC52

http://species-id.net/wiki/Medomega_bailmeup

Figs 72A-E 77 78 79 80 81

#### Type material.

**Holotype** male, AUSTRALIA: **Northern Territory:** Keep River National Park, Bail-Me-Up Creek, 23.7 km SSW Jarrnarm Camp Ground [-15.9652, 129.0311], 13–20.vi.2001, M.E. Irwin, F.D. Parker, C. Lambkin, Malaise in dry creek bed (ANIC).

#### Paratypes.

AUSTRALIA: **Northern Territory:** 20 males, 5 females, Keep River National Park, Bail-Me-Up Creek, 23.7 km SSW Jarrnarm Camp Ground [-15.9652, 129.0311], 13–30.vi.2001, M.E. Irwin, F.D. Parker, C. Lambkin, Malaise in dry creek bed (ANIC, QM, CAS). ; 4 males, Hazard Creek, 23.7 km SSW Jarrnarm Camp Ground; Malaise; 3-9.vi.2001 ME Irwin, FD Parker, C Lambkin 15°57'33"S, 129°01'44"E (GPS) (MEI_163688, 163690, 163691, 163692, ANIC); 4 males, Jarrnarm Camp Ground; pan trap; 8.vi.2001; FD Parker, ME Irwin, 15°45'44"S, 129°05'55"E (GPS) (MEI_163679, 163680, 163681, 163684, ANIC); 4 females, 3.7 km S Jarrnarm junction; flight intercept trap; 31.v-9.vi.2001; R. Oberprieler, A. Calder, L. Boutin 15°47'49"S, 129°06'31"E (GPS) (MEI_164208, 164209, 164210, 164211, ANIC); 2 females, Keep River Gorge; Malaise in rocky dry river bed; 11-20.vi.2001 ME Irwin, FD Parker, C Lambkin 15°50'00"S, 129°06'35"E(GPS) (MEI_164266, 164267, ANIC); 3 males, Limestone Gorge; pan trap; 7.vi.2001; ME Irwin, FD Parker, C Lambkin 16°03'01"S, 130°24'07"E (GPS) (MEI_163686, 163687, 163689, ANIC); 2 females, Keep River National Park; Keep River Gorge; Malaise in rocky dry river bed; 11/20-VI-2001 ME Irwin, FD Parker, C Lambkin 15°50'00"S, 129°06'35"E(GPS) (MEI_164226, 164227, ANIC).

#### Diagnosis.

Wing dark brown with white fenestration; head and body macrosetae black, coxal macrosetae white; frons with dark spots along eye margins; scutum with narrow medial stripe and irregular tessellate pattern laterally; scape yellow, red-brown dorsally, mostly dark setae with several pale setae basally; posterior surface of mid coxa with long pale setae; abdomen with sparse, silver pubescence admixed with sparse, erect pale setae especially laterally (darker posteriorly).

#### Description.

Body length= 5.0–5.5 mm (male), 5.0–6.0 mm (female). *Head*. Frontal pubescence silver-grey, suffused with tan dorsally, large dark brown spot along eye margin; frontal profile flat; numerous, slender dark setae on lower frons below and incorporating spots, setae short in female, elongate in male; male frons width at narrowest point slightly wider than ocellar tubercle, wider in female; ocellar tubercle black pubescent with dense patch of anteriorly directed setae; single row of black postocular setae, female with additional single setae posterior to row; occipital pubescence silver-grey with slight tan suffusion; parafacial and genal setae slender, admixed brown and white, elongate in male; antennal scape longer than to flagellum, bulbous, yellow with red-brown suffusion dorsally, setae black, few setae white ventrally near base; flagellum orange-yellow. *Thorax*. Scutal pubescence grey with brown markings, narrow medial stripe, irregular tessellate pattern laterally, scattered short, black setae, longer and paler posteriorly, especially in male; scutal macrosetae black; pleuron brown, overlain with grey pubescence; katatergite with admixed white and black setae; anepisternum with grey-brown marking dorsally; coxae dark, overlain with grey pubescence, setae white; mid coxa with slender setae on posterior surface; femora dark brown with grey pubescence, yellow apically, short dark setae admixed with longer pale setae, especially posteriorly, admixed with black setae on fore femur; tibiae yellow, dark brown bands apically and basally; tarsi dark brown, first and second segments yellow basally; wing dark brown fenestrate, fenestrations white, irregular dappled with more than one per cell; scutal chaetotaxy (pairs): np, 3; sa, 1; pa, 1; dc, 2; sc, 1. *Abdomen*. Male abdomen base colour dark brown, grey pubescent laterally, without dense silver velutum on tergites, elongate pale setae, female abdomen dark brown, grey pubescent laterally, female intersegmental membrane distinctly pale on segments 2–3, setae uniformly short and dark; terminalia orange to dark yellow (brown suffusion in male). Male genitalia. Gonocoxite with sparse pubescence; gonostylus not forked; outer gonocoxal process relatively short and rounded apically; dorsal apodeme of parameral sheath ‘T’-shaped; ventral apodeme subequal length to dorsal apodeme; hypandrium as a band joining to separate halves of separate gonocoxites.

#### Comments.

*Medomega bailmeup* sp. n. is known from a single collecting event in the Northern Territory and is morphologically similar to *Medomega nebrias* sp. n. based on the fenestrate wing markings, scutal markings and antennal shape. It can easily be differentiated from the latter by the dark spots laterally on the frons and setae on the posterior surface of the mid coxa.

#### Etymology.

The species epithet is derived from the type locality of this species.

### Medomega chlamydos sp. n.

urn:lsid:zoobank.org:act:9851AA0D-0C52-4B9A-8D9B-0F2E31228AB9

http://species-id.net/wiki/Medomega_chlamydos

Figs 82 83 84

#### Type material.

**Holotype** male, AUSTRALIA: **Western Australia:** Kennedy Range N.P., Malaise at edge of Falls pool [-24.645, 115.173], 26.iv–10.v.2003, M. E. Irwin, F. D. Parker (WAM).

**Paratype.** AUSTRALIA: **Western Australia:** male, Mt. Augustus N.P., Malaise at Edney’s Springs [-24.365, 116.87], 25.iv–7.v.2003, M. E. Irwin, F. D. Parker (CAS).

#### Diagnosis.

Wing white translucent with irregular apical brown mottling; all head and body setae white; scutum with narrow medial stripe; scape dark with silver-grey pubescence; posterior surface of mid coxa without setae; extensive appressed setae on abdomen.

#### Description.

Body length= 6.5–7.0 mm (male). *Head*. Male frontal pubescence silver-grey, flat to slightly rounded in profile, strip of fine white setae along eye margin; male frons width at narrowest point narrower than anterior ocellus but not contiguous; male with single row of white postocular macrosetae immediately laterad of ocellar tubercle (additional row of smaller setae anteriorly); occipital pubescence grey, narrow triangular marking medially; genal and parafacial setae white; antennal scape equal to flagellum in length, overlain with dense silver-grey pubescence admixed with numerous large white setae; flagellum orange-yellow, terminus dark. *Thorax*. Scutal pubescence silver-grey with narrow brown medial stripe and irregular tessellate markings laterally; numerous elongate white setae covering scutum, macrosetae white, setal bases dark; pleuron with silver-grey pubescence; katatergite setae uniformly white; anepisternum with grey-brown marking; coxae dark, overlain with silver-grey pubescence, setae mostly white, mid coxa without setae on posterior surface; femora dark grey-brown, apices yellow, dense long white setae on anterior and posterior surfaces, hind femur with two *av* setae apically; tibiae yellow midway, dark grey-brown apically and basally; tarsi brown, basitarsus dark yellow basally; wing white translucent, faint mottled infuscation apically and along wing veins;scutal chaetotaxy (pairs): np, 3; sa, 1; pa, 1; dc, 3; sc, 1. *Abdomen*. Male abdomen base colour darkish, obscured by dense silver velutum on tergites 2–7, admixed with extensive, appressed, long silver-white setae; terminalia dark yellow. *Male genitalia*. Gonocoxite with membranous medial atrium and sparse pubescence laterally; outer gonocoxal process relatively broad and spatulate.

#### Comments.

*Medomega chlamydos* sp. n. can be distinguished by the extensive silver-white pile on the head and body, white macrosetae, wings mostly white translucent and lack of setae on the posterior surface of the mid coxa. The latter character is present in the closely related *Medomega averyi* sp. n. Both *Medomega chlamydos* sp. n. and *Medomega averyi* sp. n. share distinctive characteristics which suggest adaptation to a hot arid climate, including extensive white or silvery pile and white translucent wings, features found in other desert inhabiting species, which reflect rather than absorb solar radiation and presumably help regulate body temperature. The female is unknown for this species.

#### Etymology.

The species epithet is from the Greek, *chlamydos*, mantle, cloak; referring to the extensive white pile on the body.

### Medomega danielsi sp. n.

urn:lsid:zoobank.org:act:7FFF180F-3F9C-4604-BEE2-262274309ACE

http://species-id.net/wiki/Medomega_danielsi

**Figure 71.**
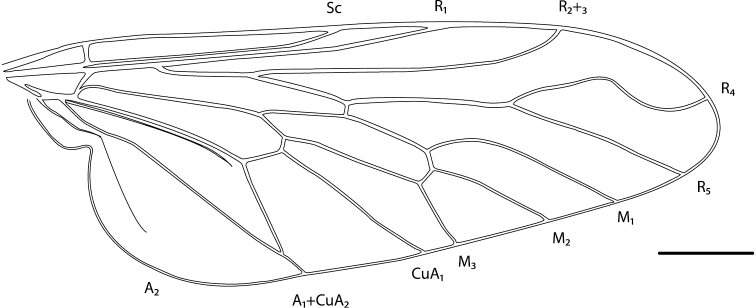
*Medomega danielsi* sp. n., wing. Scale line = 0.2 mm.

**Figure 72.**
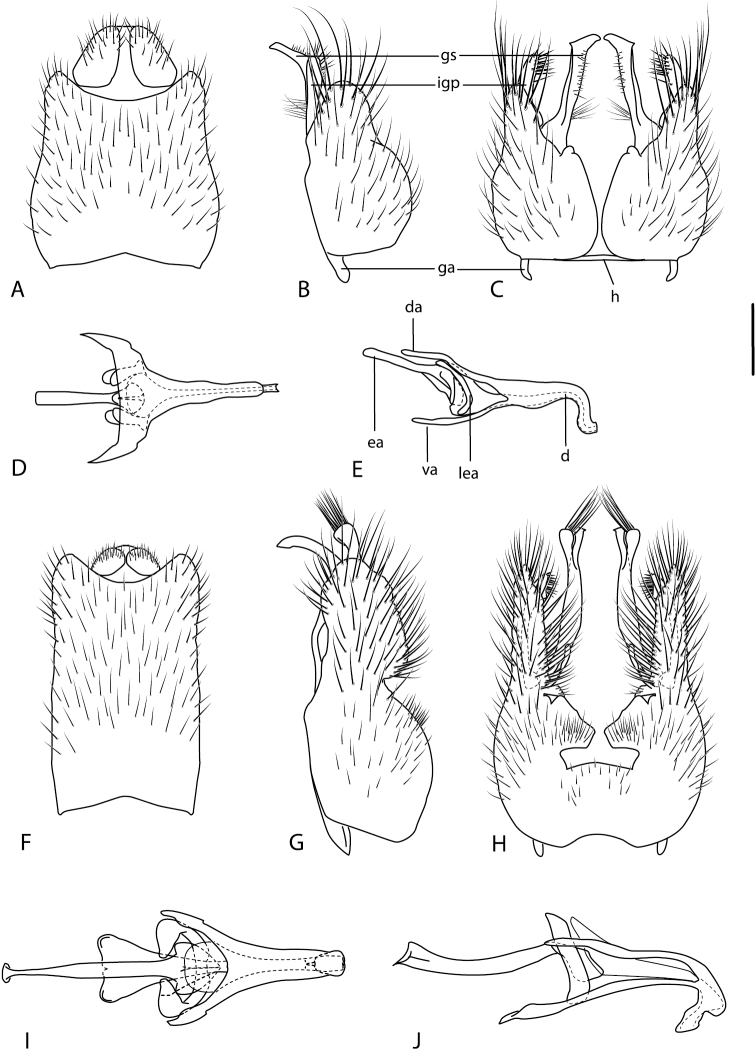
*Medomega* spp., male genitalia. *Medomega bailmeup* sp. n.: **A** epandrium, dorsal **B** gonocoxites, lateral **C** gonocoxites, ventral **D** aedeagus, dorsal **E** aedeagus, lateral. *Medomega danielsi* sp. n.: **F** epandrium, dorsal **G** gonocoxites, lateral **H** gonocoxites, ventral **I** aedeagus, dorsal **J** aedeagus, lateral. Scale line = 0.2 mm.

Figs 71 72F–J 85 86 87 88

#### Type material.

**Holotype** male, AUSTRALIA: **Queensland:** Lake Broadwater, nr. Dalby, site A, 27°21'S, 151°06'E [-27.35, 151.1], 2.v.1987, G. & A. Daniels, mv lamp (MEI_030092, AMS).

**Paratypes.** AUSTRALIA: **Queensland:** 2 females, Lake Broadwater, nr. Dalby, site A, 27°21'S, 151°06'E [-27.35, 151.1], 2.v.1987, G. & A. Daniels, mv lamp, (MEI_030094, CAS) (MEI_30095, AMS); male, 5 km N Leyburn, 27°58'S, 151°38'E [-27.96, 151.63], 2.ix.1993, 450m mv lamp, G. & A. Daniels, C. J. Burwell (MEI_033678, CAS).

#### Diagnosis.

Wing brown infuscate with white fenestration; head and body macrosetae mostly black, coxal macrosetae white; scutum with broad medial stripe and irregular tessellate pattern laterally; parafacial setae absent; male frons wider than anterior ocellus at narrowest point; scape yellow with sparse grey pubescence; posterior surface of mid coxa without setae; abdomen with silver velutum but without appressed setae.

**Description.** Body length= 6.0 mm (male), 6.0–6.5 mm (female). *Head*. Frontal pubescence silver-grey with dark brown markings, small brown spot medially and above antenna; frons profile flat; small patch of short, dark setae close to eye margin (male), or more scatter across upper frons (female); male frons width at narrowest point slightly wider than anterior ocellus; male with single row of black postocular setae, additional setae irregularly arranged medially, white setae ventrally; occipital pubescence grey; parafacial setae absent, genal setae dark, relatively short, longer ventrally; antennal scape slightly shorter than flagellum, orange-yellow with sparse grey pubescence, black setae dorsally, yellow ventrally and laterally; flagellum orange-yellow. *Thorax*. Scutal pubescence silver-grey velutum with darker grey pattern and dark setal bases, broad dark medial stripe anteriorly, narrowed posteriorly, lateral stripes broken to tessellate, scattered short dark setae, longer and paler posteriorly; scutal macrosetae dark; pleuron with silver-grey pubescence; katatergite setae uniformly pale; anepisternum with brown marking dorsally and ventrally; coxae dark, overlain with silver-grey pubescence, setae pale, mid coxa without setae on posterior surface; femora dark brown, suffused yellow basally on mid and hind femora, yellow apically, uniform short dark setae; tibiae yellow, dark brown apically and basally; tarsi brown, yellow basally on basal segments; wing dark infuscate with white fenestration (darker in female); scutal chaetotaxy (pairs): np, 3; sa, 1; pa, 1; dc, 2-3; sc, 1. *Abdomen*. Male abdomen base colour darkish obscured by extensive silver velutum on tergites 2–7, vestiture mostly elongate pale setae, slightly denser laterally; terminalia brown or dark yellow, female abdominal markings with tergites dark brown dorsally, grey pubescent posterolaterally; intersegmental membrane between tergites 1–2, and 2–3 distinctly pale coloured, well defined. *Male genitalia*. Epandrium quadrangular, elongate; gonocoxite with sparse pubescence; gonocoxite fused medially with hypandrium; outer gonocoxal process very broad and spatulate; inner gonocoxal process shorter than outer gonocoxal process, apically bearing medially directed short dark setae; gonostylus forked, ventral arm with tuft of elongate macrosetae in a comb.

#### Comments.

This species is known southeastern Queensland, just west of the Great Dividing Range. The wing and scutal markings are distinctive, along with the elongate male genitalia with a forked gonostylus. The gonocoxites are fused medially, which appears to be a rare character in this genus.

#### Etymology.

This species is named in honour of Greg Daniels, the collector of this species.

### Medomega gigasathe sp. n.

urn:lsid:zoobank.org:act:9851AA0D-0C52-4B9A-8D9B-0F2E31228AB9

http://species-id.net/wiki/Medomega_gigasathe

Figs 89 90 91 92

#### Type material.

**Holotype** male, AUSTRALIA: **Western Australia:** 57 km S Norseman, 32°38'S, 121°32'E [-32.633, 121.533], 30.xii.1985, G. & A. Daniels, mv lamp (AMS).

#### Diagnosis.

Wing white with irregular brown fenestrate mottling; most head and body macrosetae black, coxal macrosetae admixed black and white; scutum dark yellow-tan with dark setal bases; scape yellow; posterior surface of mid coxa without setae; abdomen with silver velutum and numerous white, erect setae; male genitalia greatly enlarged.

#### Description.

Body length= 7.5 mm (male). *Head*. Frontal pubescence tan-brown, small dark brown spot adjacent to eye margin, profile flat, small patch of very short, dark setae close to eye margin; male frons width at narrowest point narrower than anterior ocellus but eyes not contiguous; single row of black postocular setae immediately laterad of ocellar tubercle; occiput overlain with grey-tan pubescence; parafacial setae dark, genal setae dark (longer and pale medially); proboscis elongate, extending anteriorly beyond antenna; antennal scape longer than flagellum, black setae dorsally, yellow ventrally and laterally; flagellum orange-yellow, distal portion dark. *Thorax*. Scutal pubescence grey-tan with setal bases dark brown, scattered short dark setae, longer and paler posteriorly; scutal macrosetae dark; pleuron with silver-grey pubescence; katatergite setae uniform white; anepisternum with grey-brown marking dorsally; coxae yellow, overlain with silver-grey pubescence, mixture of dark and pale setae, mid coxa without setae on posterior surface; femora dark yellow with brown suffusion, apices dark brown, uniform short dark setae; tibiae yellow, dark grey-brown apically; tarsi dark yellow with apices brown (tarsomeres 3-5 brown); wing white translucent, irregularly fenestrate; scutal chaetotaxy (pairs): np, 3; sa, 1; pa, 1; dc, 3; sc, 1. *Abdomen*. Male abdomen base colour darkish, obscured by extensive silver velutum on tergites 2–7, admixed with elongate pale setae, denser laterally, terminalia dark yellow (greatly enlarged). *Male genitalia*. Greatly elongated with epandrium overhanging gonocoxites; gonocoxites fused anteriorly and with large media atrium posteriorly with velutum-covered membrane; gonostylus elongate and irregularly forked apically.

#### Comments.

*Medomega gigasathe* sp. n. is known from a single male specimen from Western Australia. The male genitalia are greatly enlarged, which along with the elongate mouthparts, wing markings and male abdominal velutum, are distinctive for this species.

#### Etymology.

The species epithet is derived from the Greek, *gigas* giant + *sathe* penis; referring to the relatively large genitalic capsule of this species.

### Medomega nebrias sp. n.

urn:lsid:zoobank.org:act:9851AA0D-0C52-4B9A-8D9B-0F2E31228AB9

http://species-id.net/wiki/Medomega_nebrias

**Figure 93.**
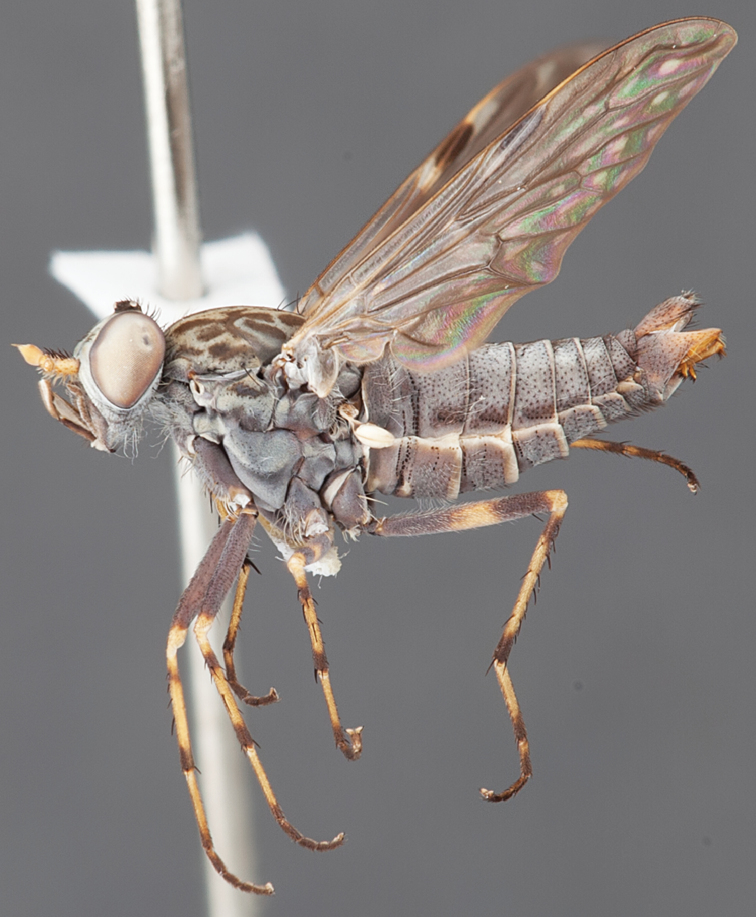
*Medomega nebrias* sp. n., male, lateral view. Body length = 7.0 mm.

Figs 93 94 95 96 97 98

#### Type material.

**Holotype** male, AUSTRALIA: **New South Wales:** Warrumbungle N.P., Wambelong Creek, 31°19.377'S, 149°01.652'E [-31.322, 149.027], 595m, 13.iii–8.iv.2008, S. L. Winterton, J. S. Bartlett, D. J. Tree, Malaise across [dry] creek bed (AMS).

**Paratypes.** AUSTRALIA: **New South Wales:** 10 males, 5 females, Warrumbungle N.P., Wambelong Creek, 31°19.377'S, 149°01.652'E [-31.322, 149.027], 595m, 13.iii–8.iv.2008, S. L. Winterton, J. S. Bartlett, D. J. Tree, Malaise across [dry] creek bed (CAS, CSCA, QM)

#### Diagnosis.

Wing dark brown with white fenestration; most head and body macrosetae black, coxal macrosetae white; scutum with broad medial stripe and irregular tessellate pattern laterally; scape yellow with sparse grey pubescence; posterior surface of mid coxa without setae; abdomen with sparse, silver pubescence admixed with sparse, erect pale setae (darker posteriorly).

#### Description.

Body length= 6.6–7.5 mm (male), 7.0–8.0 mm (female). *Head*. Frontal pubescence silver-grey with pale brown markings, small brown spot medially, suffuse light brown above antennae and along upper eye margin, female with additional brown quadrangle dorsally, incorporating ocellar tubercle; frontal profile flat; sparse, elongate patch of dark setae parallel to eye margin, male frons width at narrowest point slightly wider than ocellar tubercle; two rows of black postocular setae; occipital pubescence grey, narrow triangular marking medially; parafacial and genal setae dark (lighter posteriorly); antennal scape equal to flagellum length, yellow with grey pubescence, dark setae dorsally, pale ventrally and laterally; flagellum orange-yellow, darker patch dorsally, style dark. *Thorax*. Scutal pubescence grey with brown markings, broad medial stripe anteriorly, narrowed posteriorly, irregular tessellate pattern laterally, scattered short, black setae, longer and paler posteriorly; scutal macrosetae black; pleuron with silver-grey pubescence; katatergite with admixed white and black setae; anepisternum with grey-brown marking dorsally; coxae dark, overlain with silver-grey pubescence, setae white; mid coxa without setae on posterior surface; femora dark brown with grey pubescence, yellow patch midway and apically on mid and hind femora, short dark setae admixed with longer pale setae; tibiae yellow, dark brown bands apically and basally; tarsi dark brown, first and second segments yellow basally; wing dark brown fenestrate, fenestrations white, dappled with one per cell; scutal chaetotaxy (pairs): np, 3; sa, 1; pa, 1; dc, 3; sc, 1. *Abdomen*. Male abdomen base colour dark brown dorsally, grey pubescent laterally, without dense silver velutum on tergites, elongate pale setae, denser laterally, female abdomen dark brown, grey pubescent laterally, female intersegmental membrane distinctly pale on segments 2–3, setae uniform short and dark; terminalia orange to dark yellow (brown suffusion in male). *Male genitalia*. Epandrium quadrangular; gonocoxites separate medially without medial atrium, joined anteriorly by band-like hypandrium, extensive velutum ventrally; gonostylus not forked; outer gonocoxal process triangular; distiphallus relatively short and straight; dorsal apodeme length subequal to ventral apodeme.

#### Comments.

*Medomega nebrias* sp. n. is a relatively robust species known from Warrumbungle National Park in New South Wales. The wing and scutal markings suggest a close relationship with *Medomega bailmeup* sp. n.

#### Etymology.

The species epithet is from the Greek, *nebros*, dappled, spotted like a fawn; referring to the wing markings.

## Supplementary Material

XML Treatment for Manestella

XML Treatment for Manestella caesia

XML Treatment for Manestella campestris

XML Treatment for Manestella canities

XML Treatment for Manestella cooloola

XML Treatment for Manestella fumosa

XML Treatment for Manestella incompleta

XML Treatment for Manestella nubis

XML Treatment for Manestella obscura

XML Treatment for Manestella ocellaris

XML Treatment for Manestella persona

XML Treatment for Manestella poecilothorax

XML Treatment for Manestella tristriata

XML Treatment for Manestella umbrapennis

XML Treatment for Manestella vasta

XML Treatment for Manestella vespera

XML Treatment for Medomega

XML Treatment for Medomega averyi

XML Treatment for Medomega bailmeup

XML Treatment for Medomega chlamydos

XML Treatment for Medomega danielsi

XML Treatment for Medomega gigasathe

XML Treatment for Medomega nebrias

## Figures and Tables

**Figure 1.**
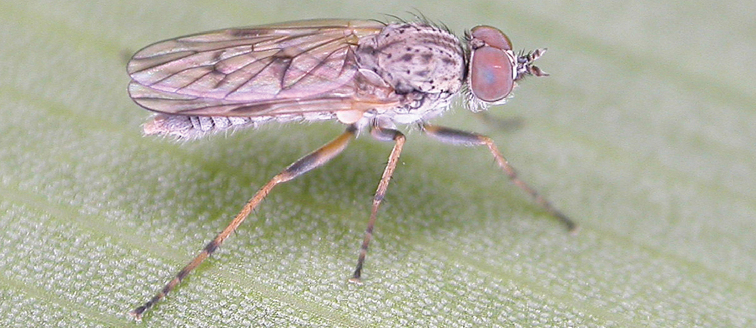
*Manestella poecilothorax* sp. n., male, Western Australia, Golden Bay dunes. Body length = 3.0 mm. Photograph credit: Shaun L. Winterton.

**Figure 2.**
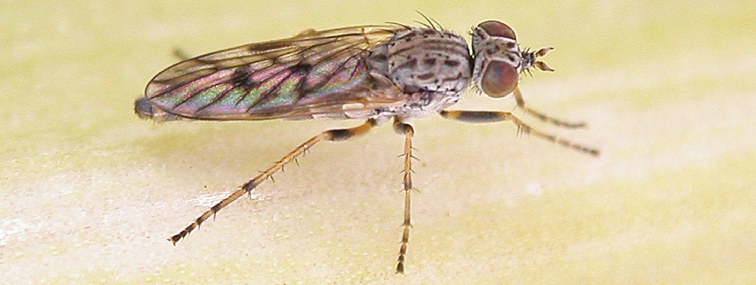
*Manestella poecilothorax* sp. n., female, Western Australia, Golden Bay dunes. Body length = 4.0 mm. Photograph credit: Shaun L. Winterton.

**Figure 3.**
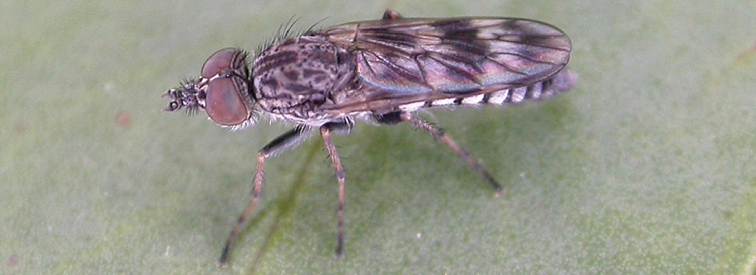
*Manestella persona* sp. n., male, Western Australia, Golden Bay dunes. Body length = 3.0 mm. Photograph credit: Shaun L. Winterton.

**Figure 5.**
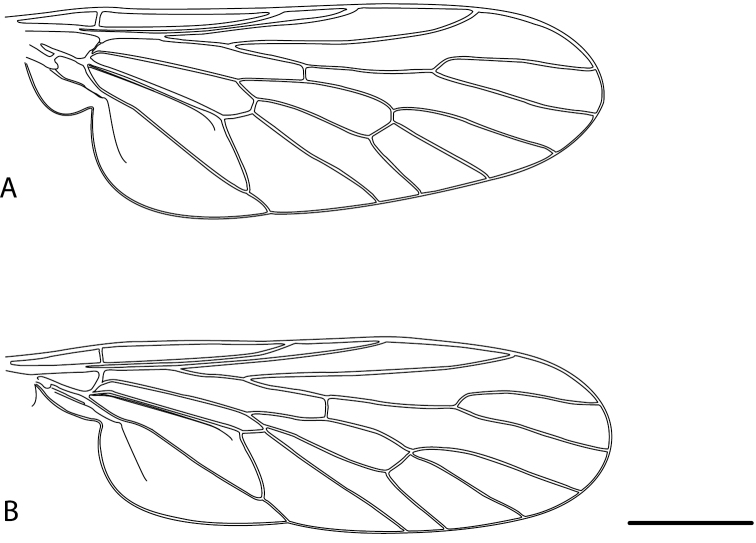
*Manestella* spp.: **A** *Manestella caesia* sp. n., wing **B** *Manestella cooloola* sp. n., wing. Scale line = 0.2 mm.

**Figure 7.**
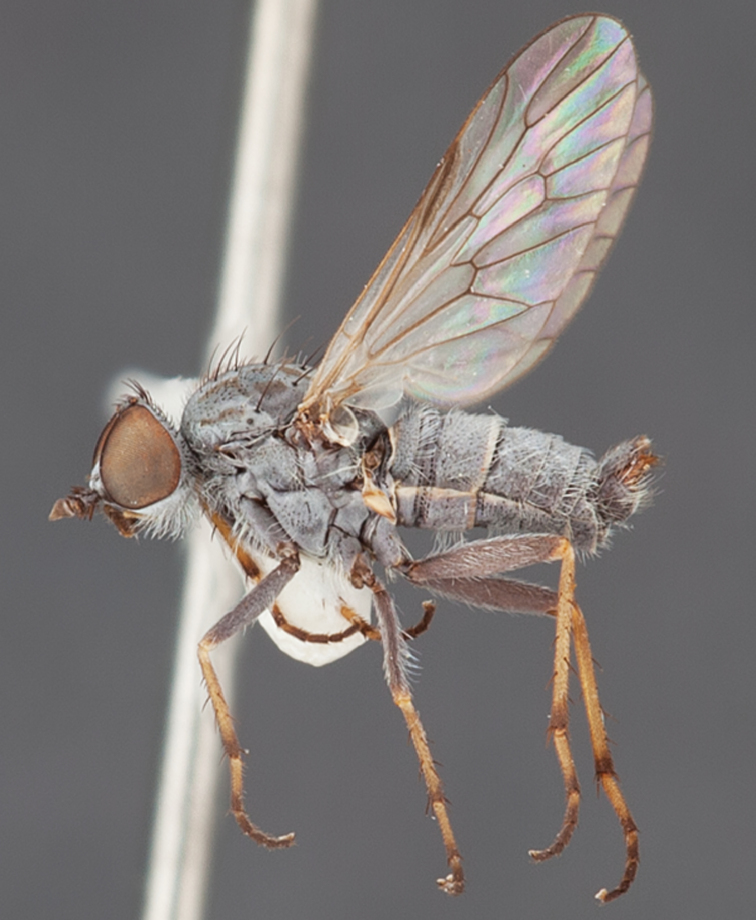
*Manestella caesia* sp. n., male, lateral view. Body length = 3.5 mm.

**Figure 8.**
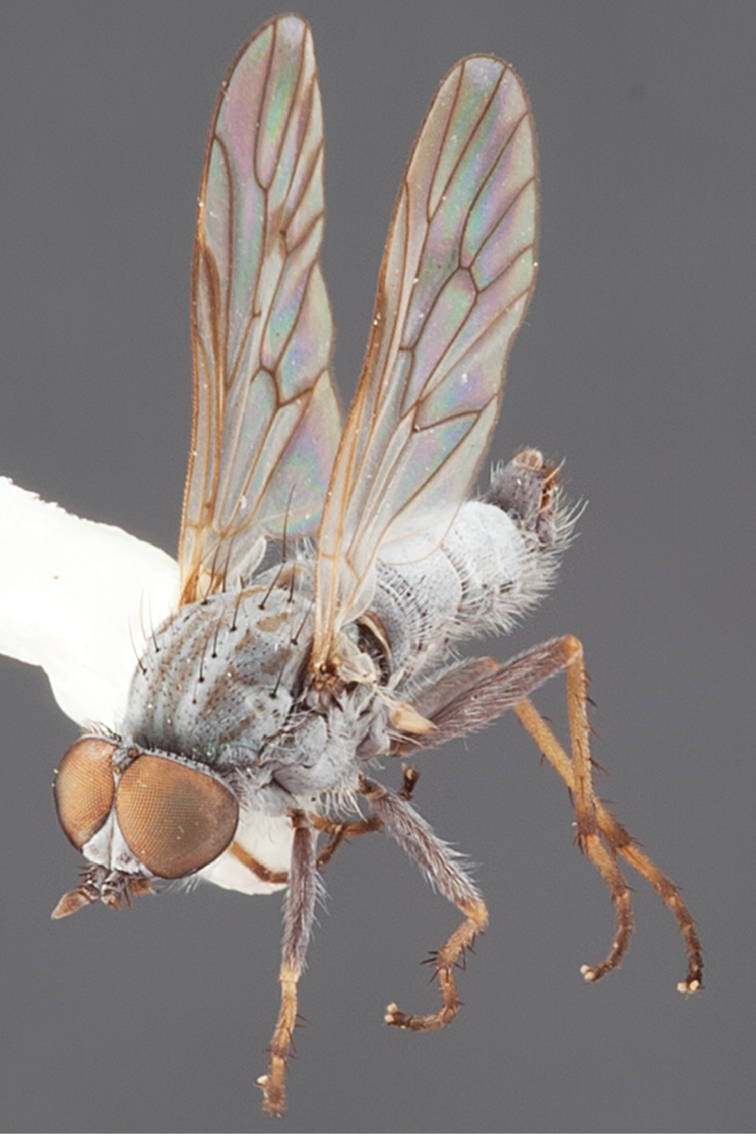
*Manestella caesia* sp. n., male, oblique view. Body length = 3.5 mm.

**Figure 9.**
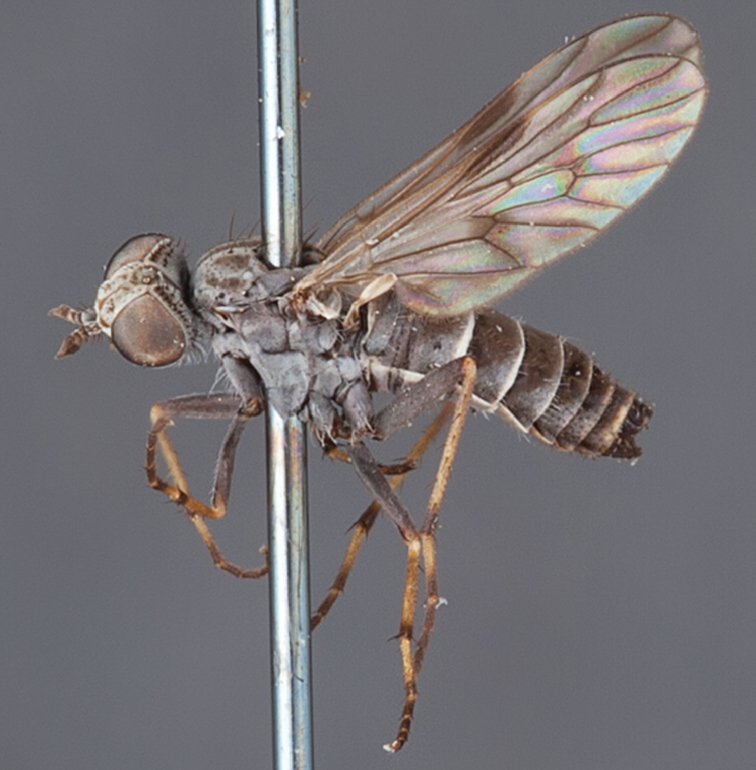
*Manestella caesia* sp. n., female, lateral view. Body length = 4.0 mm.

**Figure 10.**
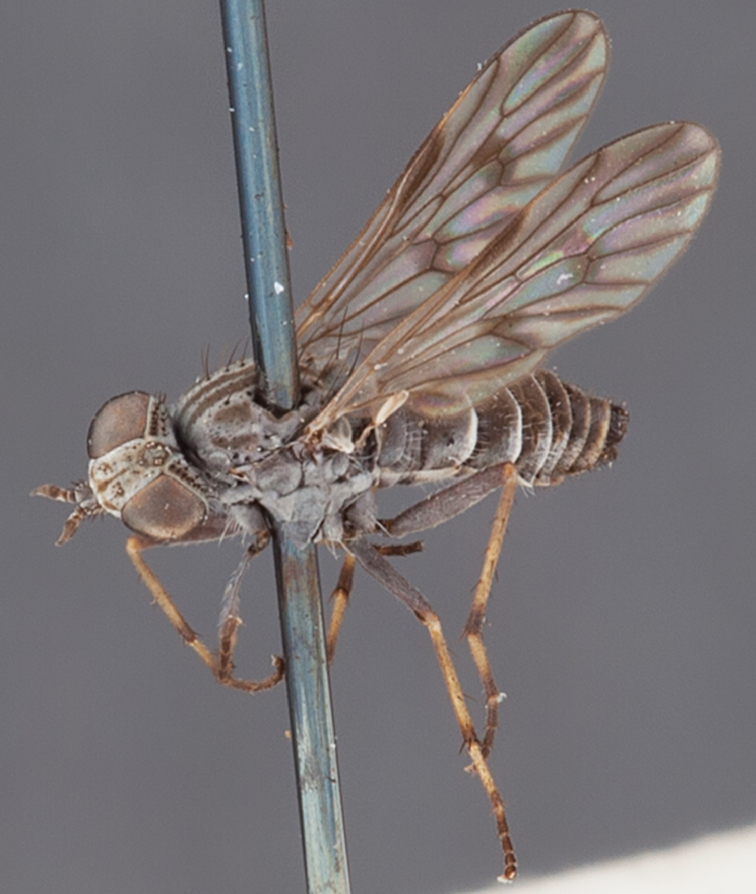
*Manestella caesia* sp. n., female, oblique view. Body length = 4.0 mm.

**Figure 11.**
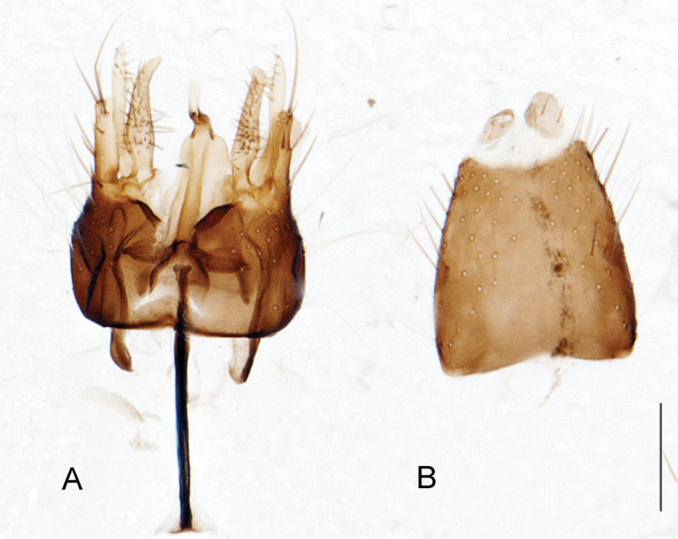
*Manestella caesia* sp. n., male genitalia **A** gonocoxites with aedeagus *in situ*, ventral view **B** epandrium. Scale line = 0.2 mm.

**Figure 12.**
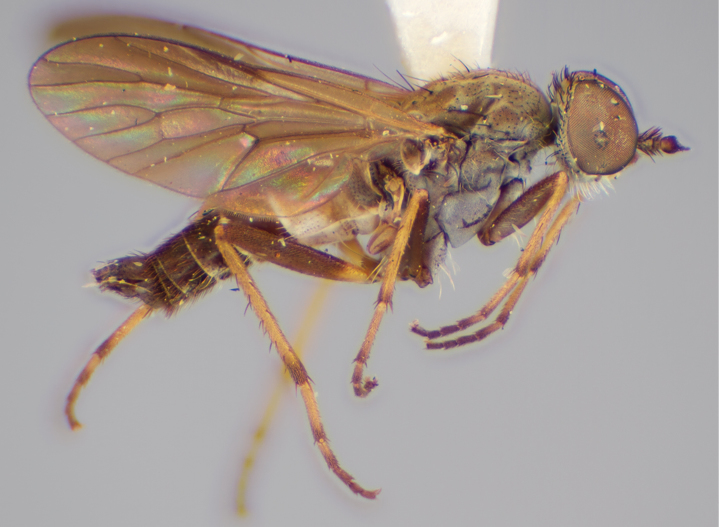
*Manestella campestris* sp. n., male, lateral view. Body length = 3.5 mm.

**Figure 13.**
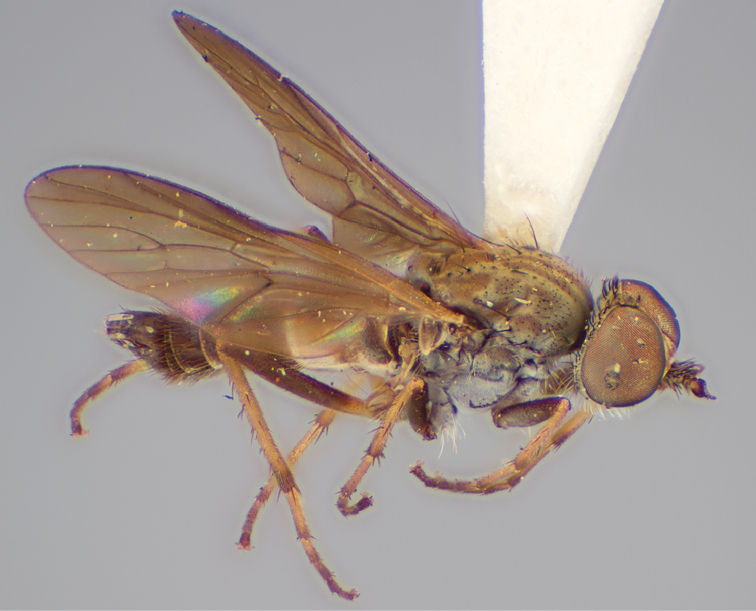
*Manestella campestris* sp. n., male, oblique view. Body length = 3.5 mm.

**Figure 14.**
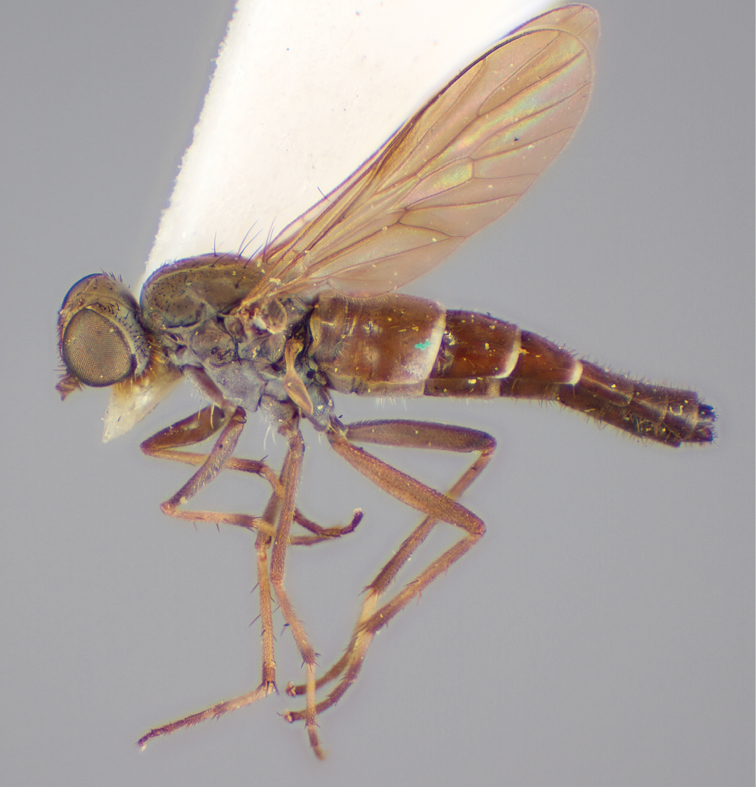
*Manestella campestris* sp. n., female, lateral view. Body length = 4.5 mm.

**Figure 15.**
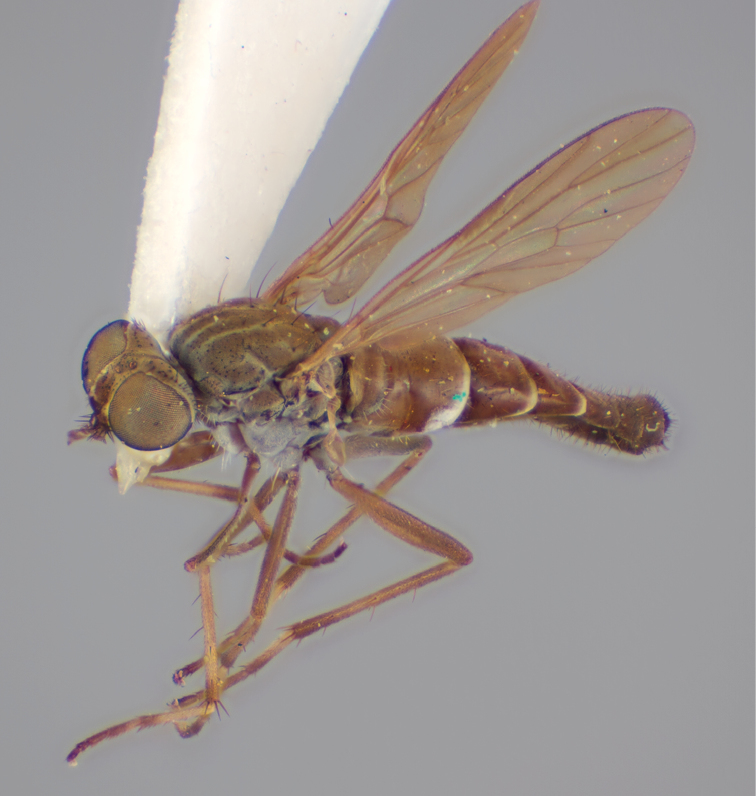
*Manestella campestris* sp. n., female, oblique view. Body length = 4.5 mm.

**Figure 16.**
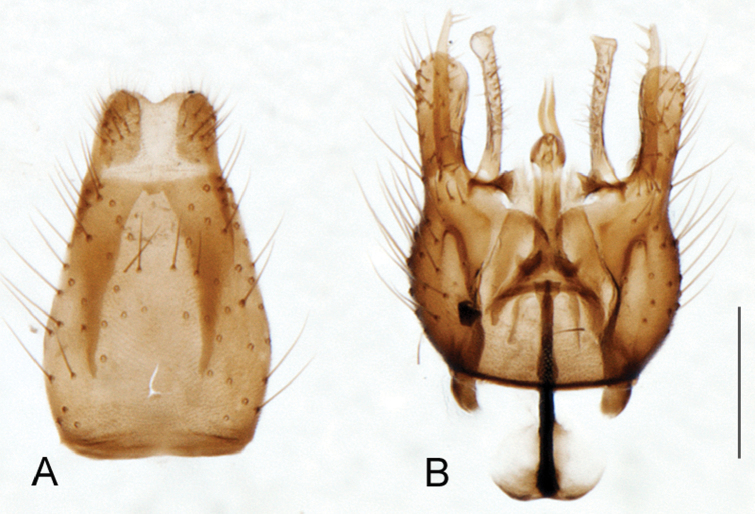
*Manestella campestris* sp. n., male genitalia **A** epandrium **B** gonocoxites with aedeagus *in situ*, ventral view. Scale line = 0.2 mm.

**Figure 17.**
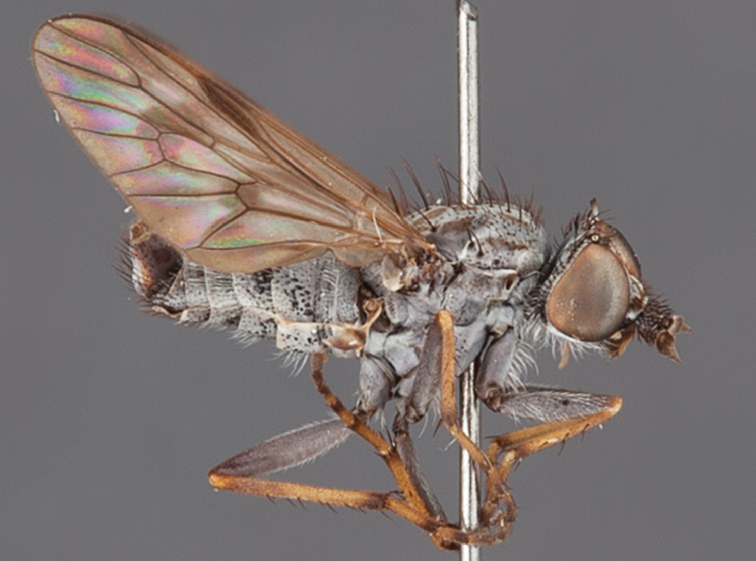
*Manestella canities* sp. n., male, lateral view. Body length = 3.5 mm.

**Figure 18.**
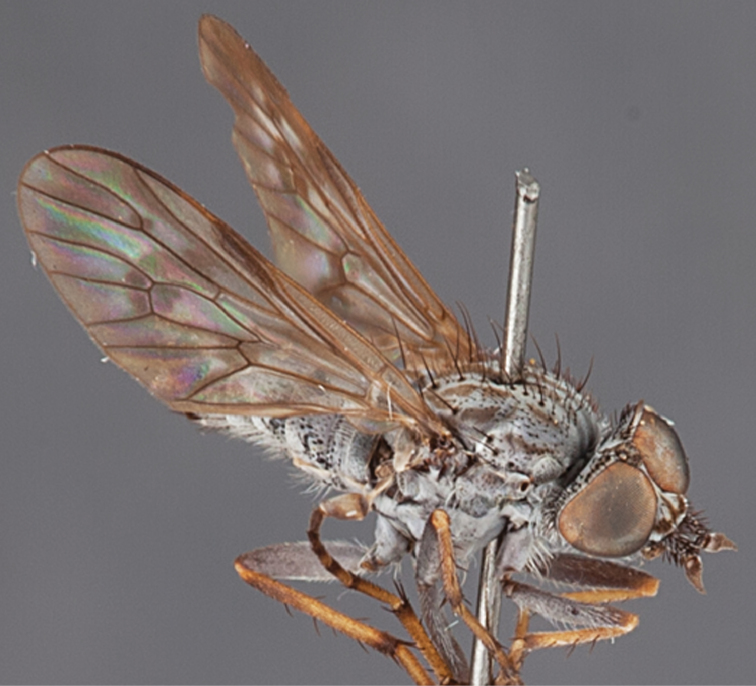
*Manestella canities* sp. n., male, oblique view. Body length = 3.5 mm.

**Figure 19.**
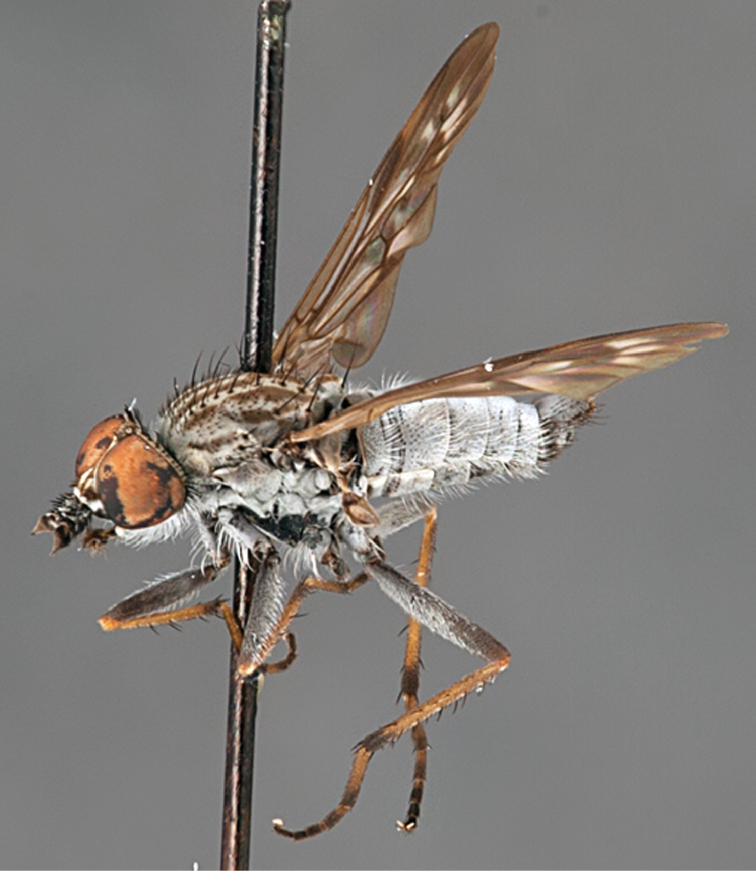
*Manestella canities* sp. n., male, oblique view. Body length = 3.5 mm.

**Figure 20.**
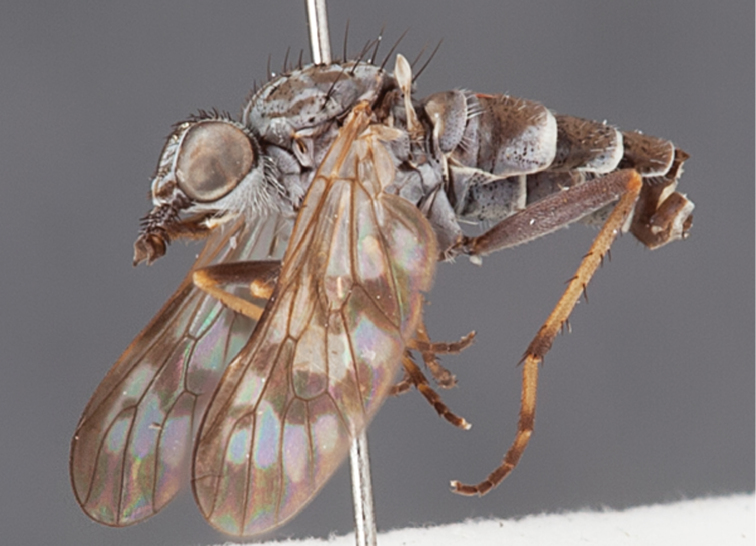
*Manestella canities* sp. n., female, lateral view. Body length = 4.2 mm.

**Figure 21.**
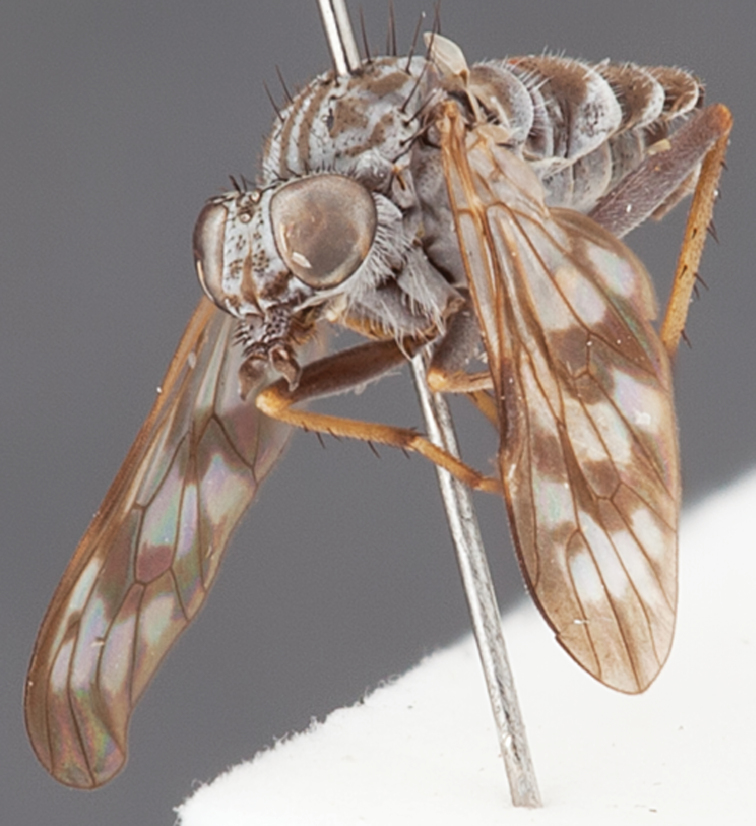
*Manestella canities* sp. n., female, oblique view. Body length = 4.2 mm.

**Figure 22.**
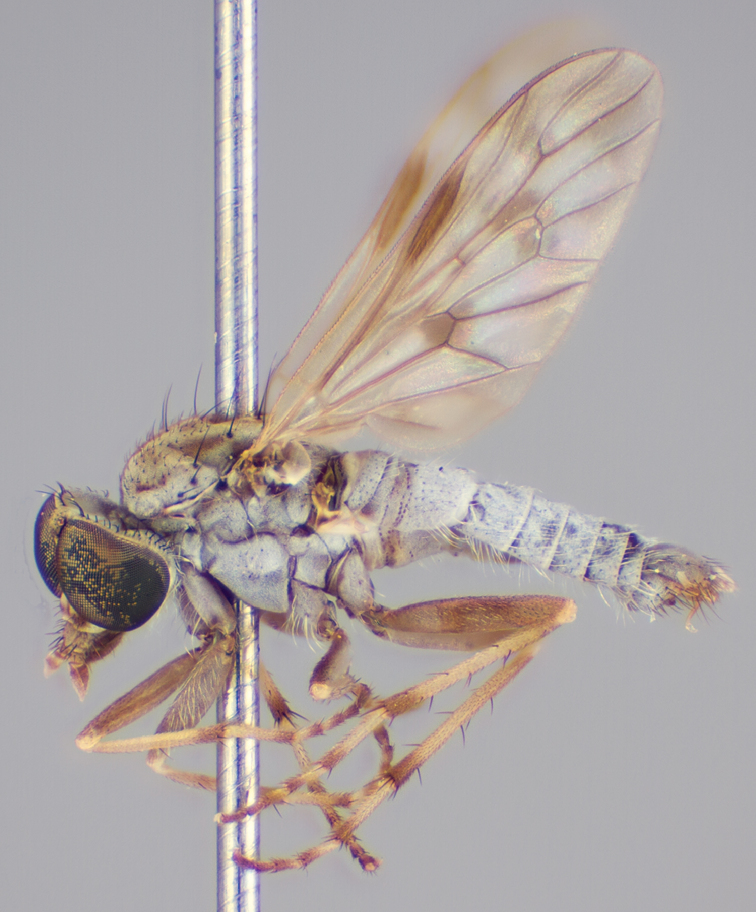
*Manestella cooloola* sp. n., male, lateral view. Body length = 2.8 mm.

**Figure 23.**
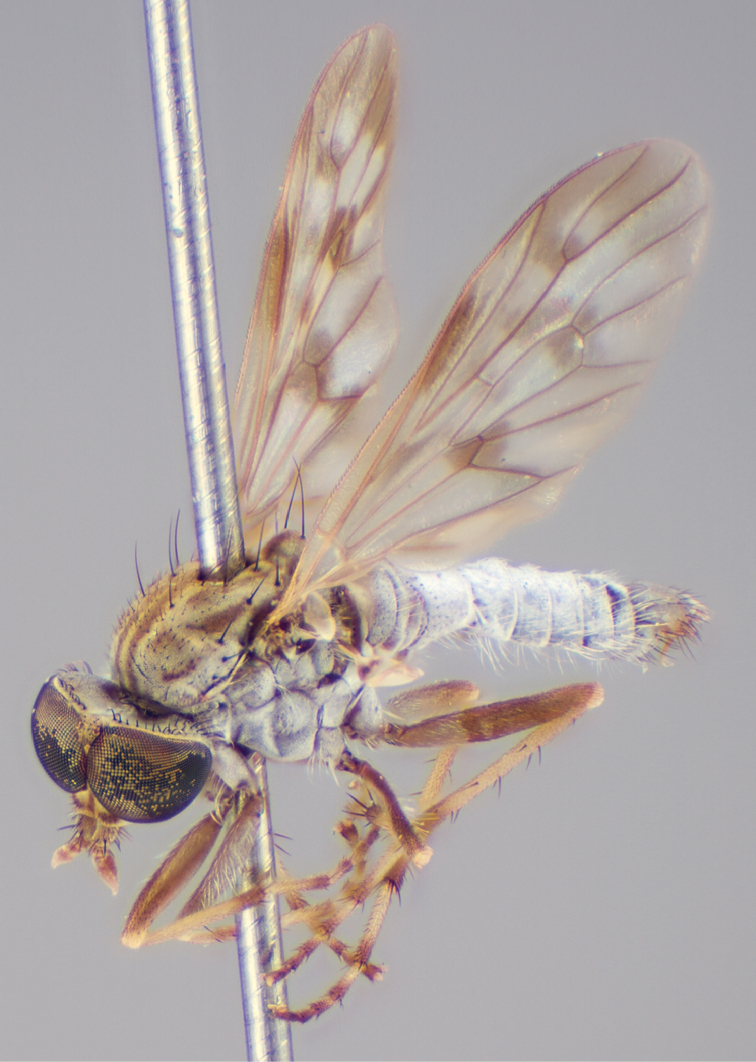
*Manestella cooloola* sp. n., male, oblique view. Body length = 2.8 mm.

**Figure 24.**
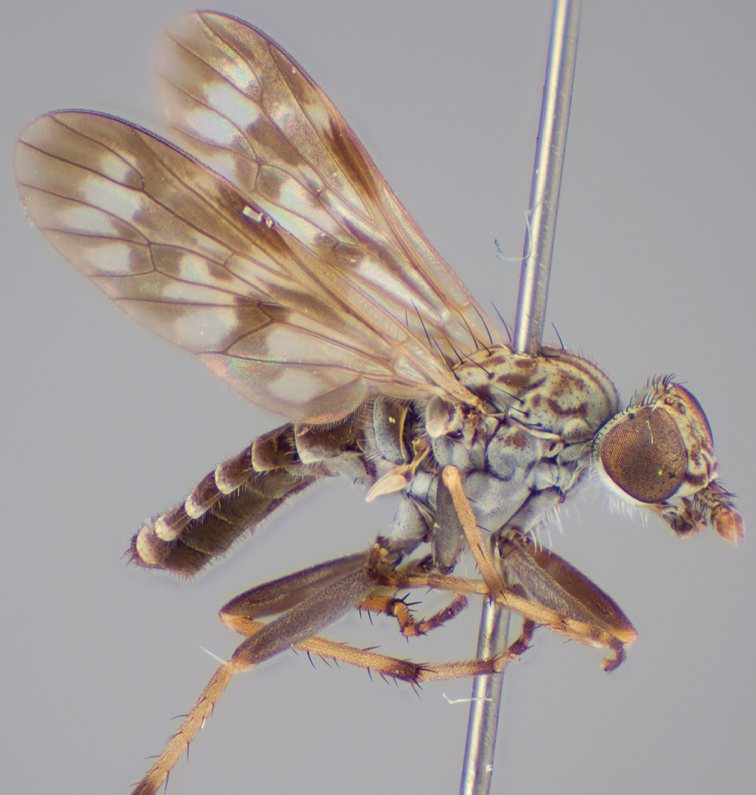
*Manestella cooloola* sp. n., female, oblique view. Body length = 4.2 mm.

**Figure 25.**
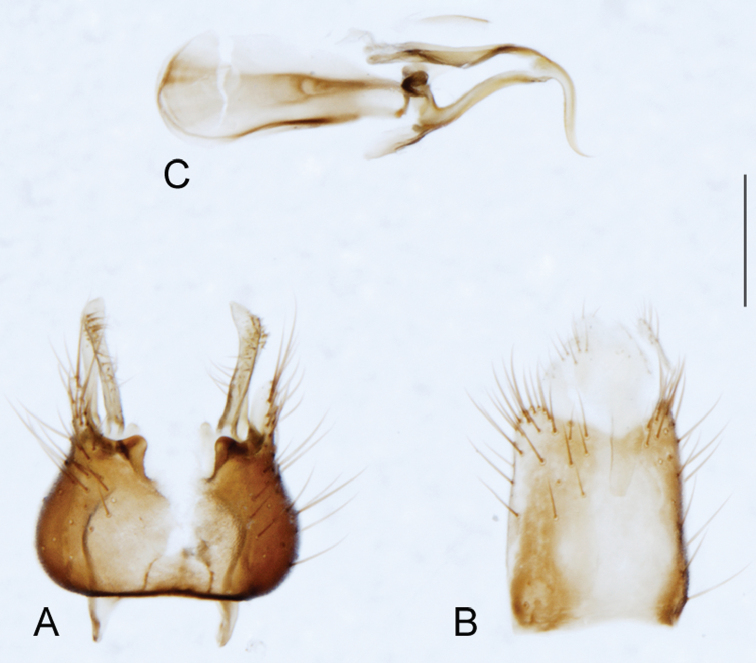
*Manestella cooloola* sp. n., male genitalia **A** epandrium **B** gonocoxites, ventral view **C** aedeagus, lateral view. Scale line = 0.2 mm.

**Figure 26.**
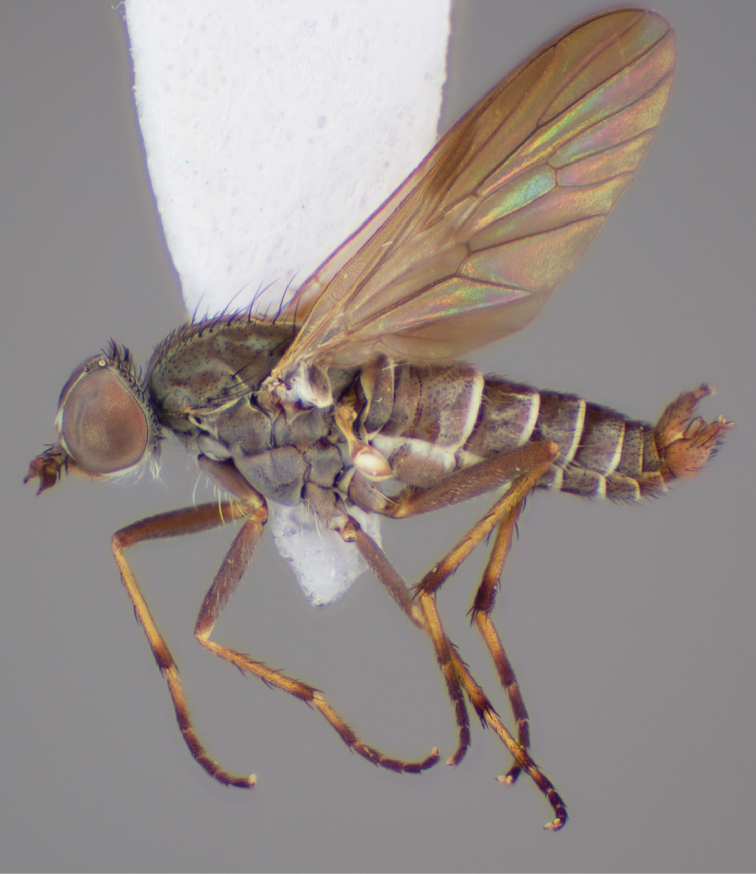
*Manestella fumosa* sp. n., male, lateral view. Body length = 3.0 mm.

**Figure 27.**
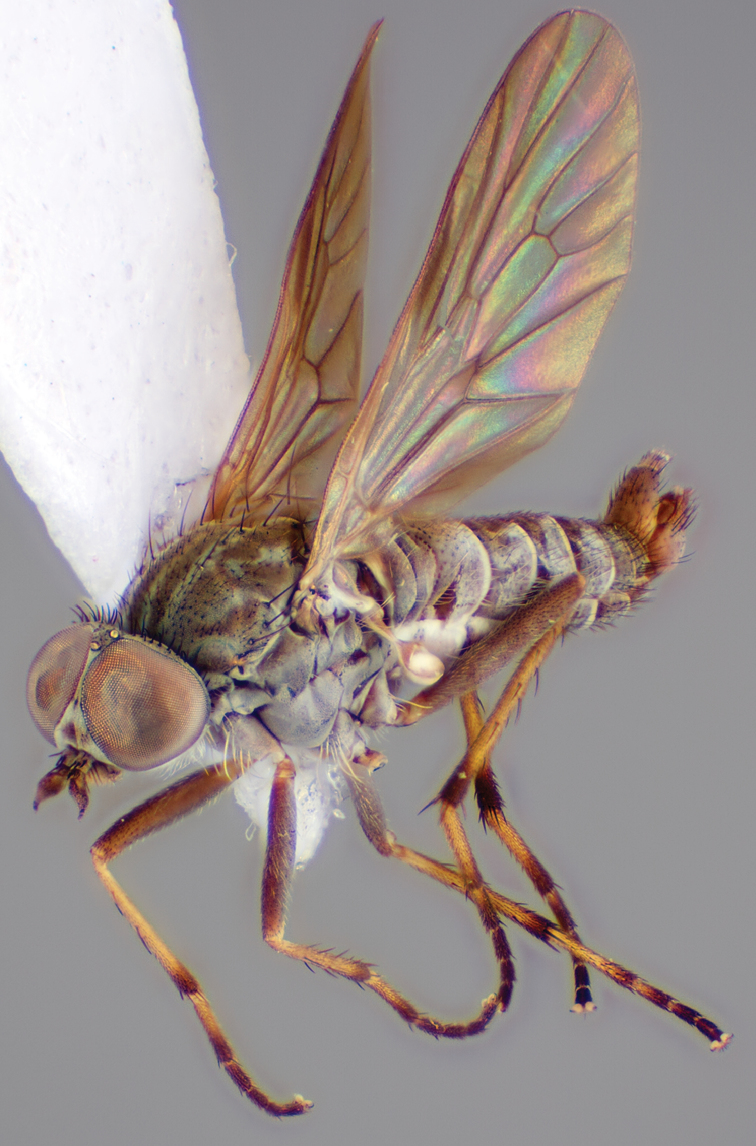
*Manestella fumosa* sp. n., male, oblique view. Body length = 3.0 mm.

**Figure 28.**
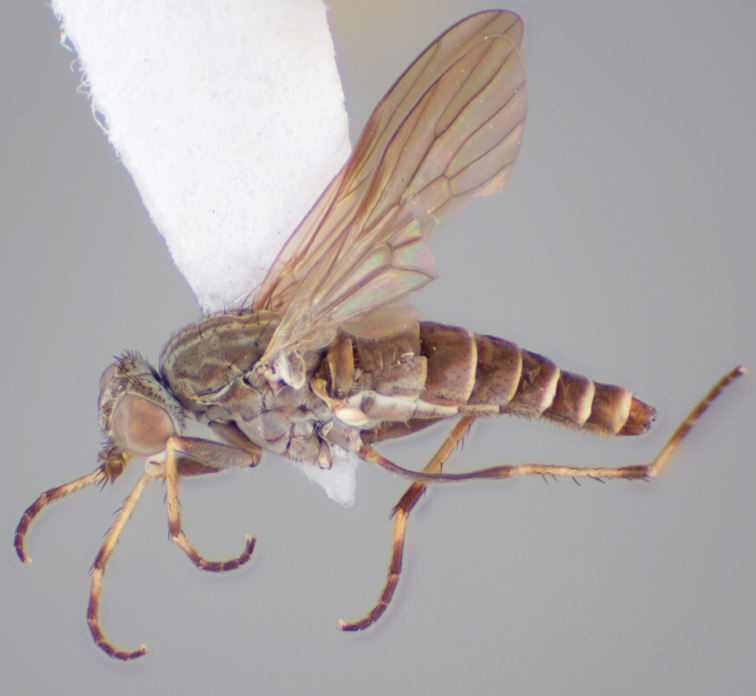
*Manestella fumosa* sp. n., female, lateral view. Body length = 4.0 mm.

**Figure 29.**
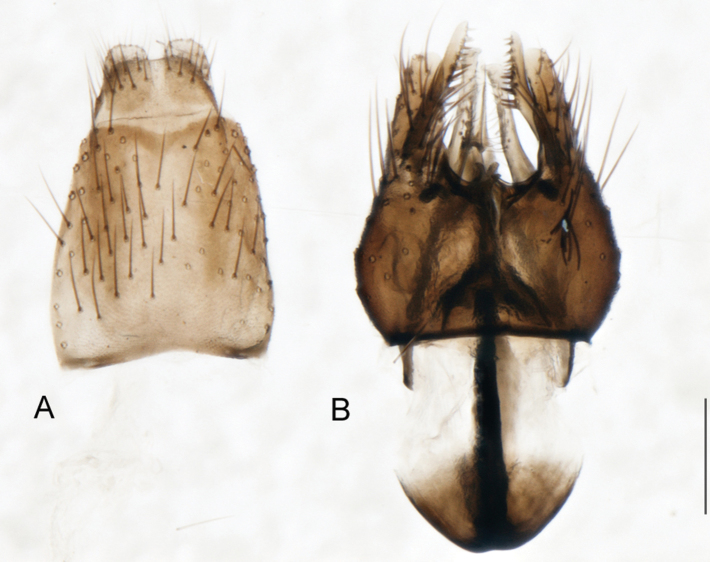
*Manestella fumosa* sp. n., male genitalia **A** epandrium **B** gonocoxites with aedeagus *in situ*, ventral view. Scale line = 0.2 mm.

**Figure 30.**
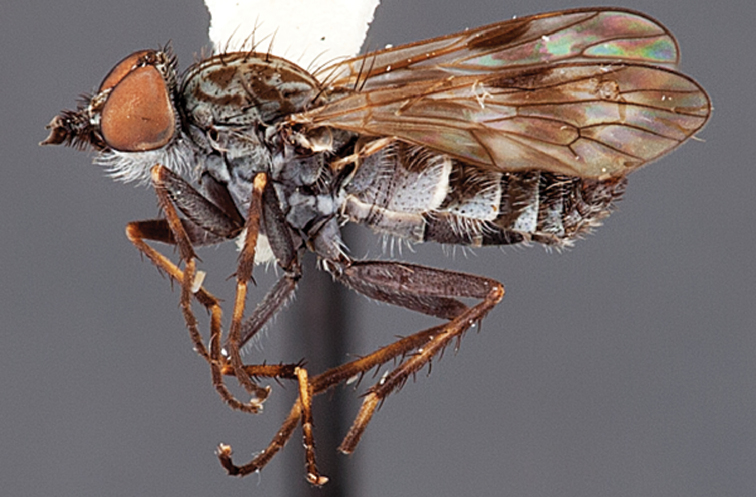
*Manestella incompleta* sp. n., male, lateral view. Body length = 3.8 mm.

**Figure 31.**
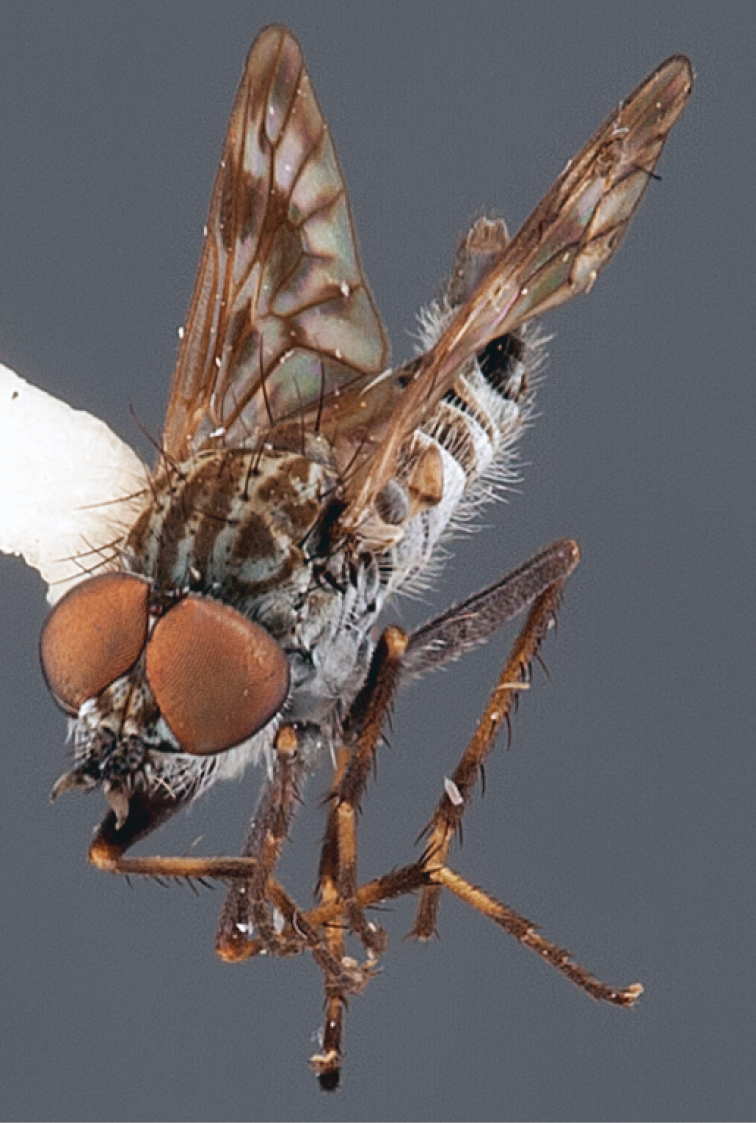
*Manestella incompleta* sp. n., male, oblique view. Body length = 3.8 mm.

**Figure 32.**
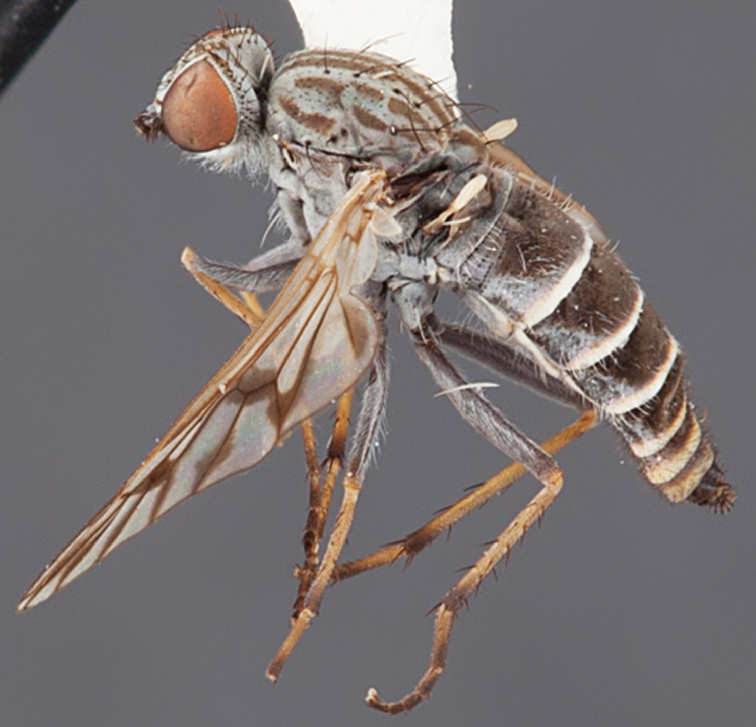
*Manestella incompleta* sp. n., female, lateral view. Body length = 4.3 mm.

**Figure 33.**
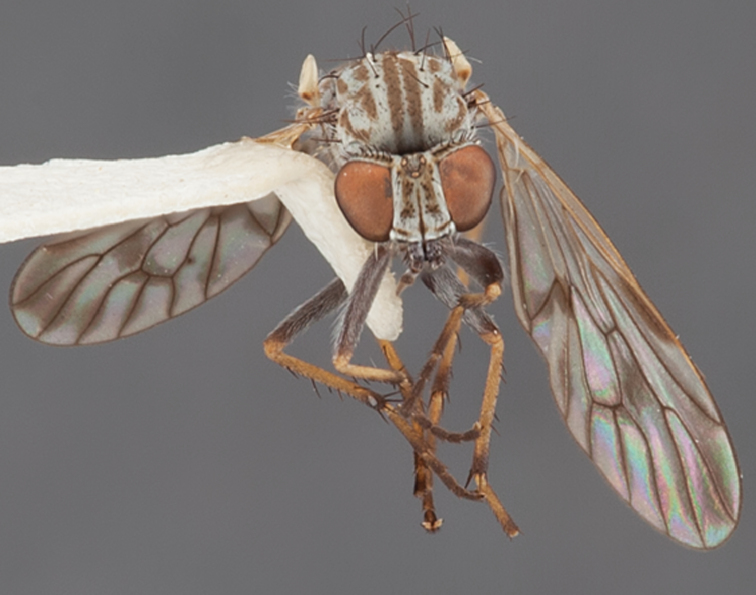
*Manestella incompleta* sp. n., female, anterior view. Body length = 4.3 mm.

**Figure 34.**
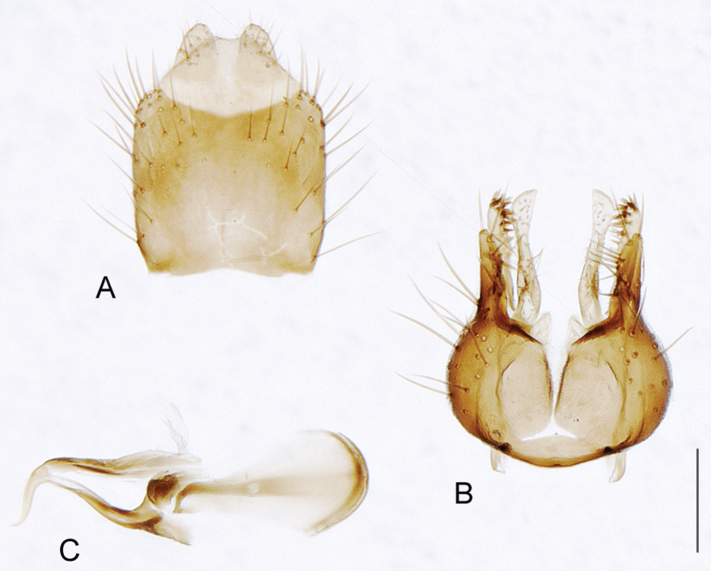
*Manestella incompleta* sp. n., male genitalia **A** epandrium **B** gonocoxites with aedeagus *in situ*, ventral view. Scale line = 0.2 mm.

**Figure 35.**
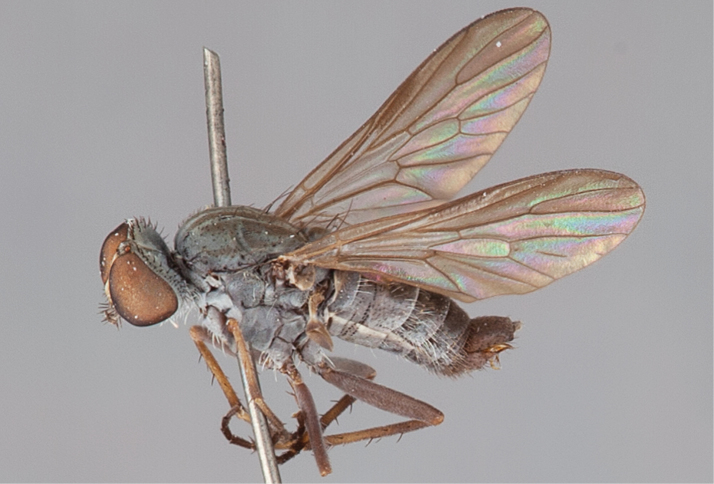
*Manestella nubis* sp. n., male, lateral view. Body length = 3.5 mm.

**Figure 36.**
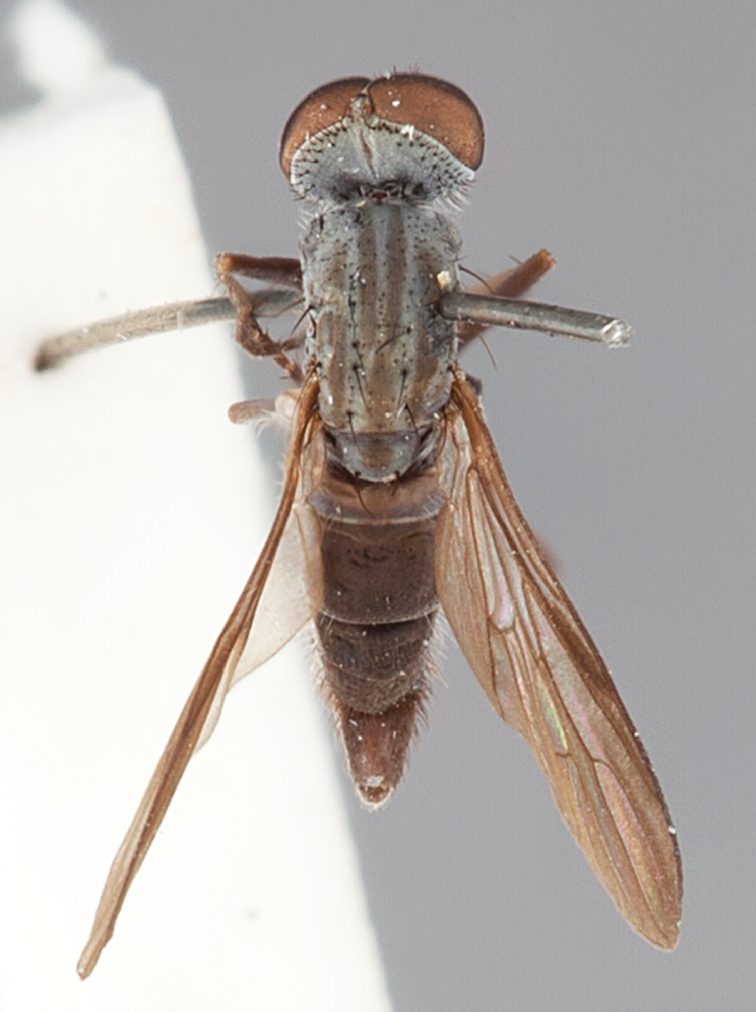
*Manestella nubis* sp. n., male, dorsal view. Body length = 3.5 mm.

**Figure 37.**
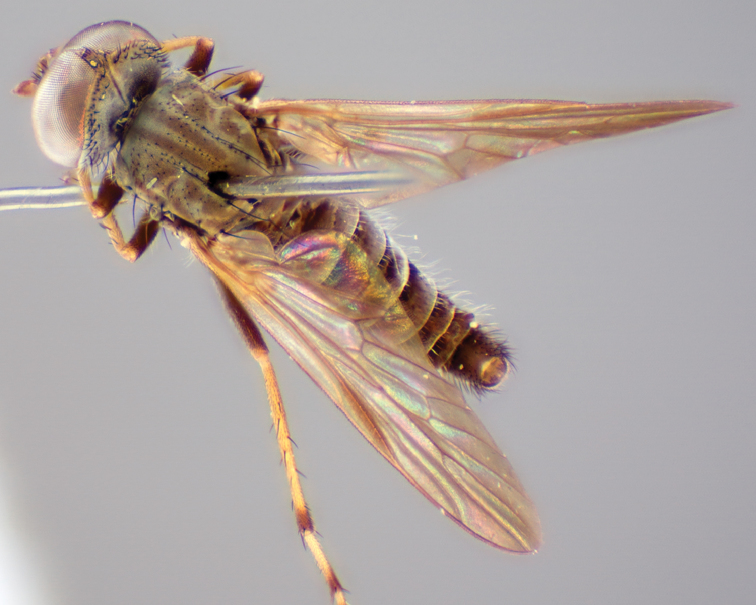
*Manestella obscura* sp. n., male, dorsal view. Body length = 3.4 mm.

**Figure 38.**
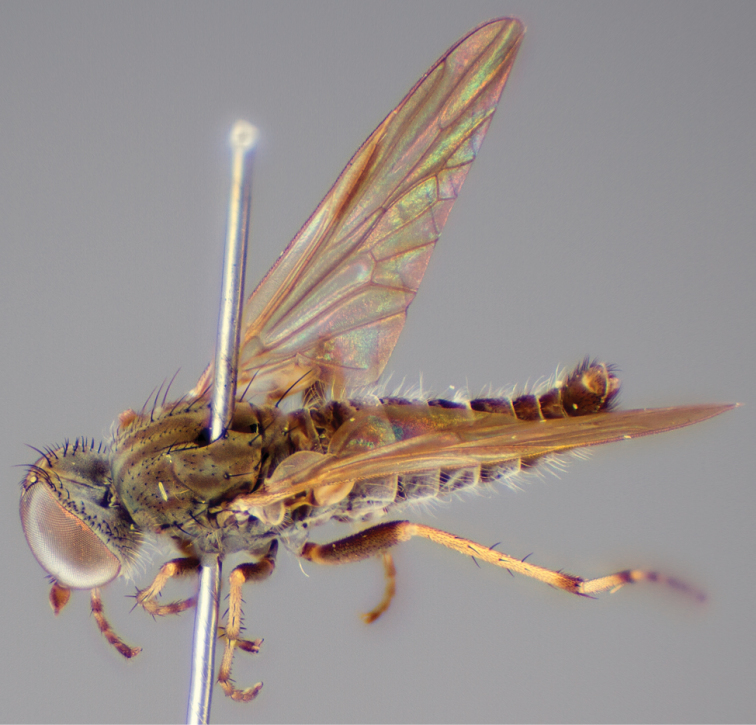
*Manestella obscura* sp. n., male, oblique view. Body length = 3.4 mm.

**Figure 39.**
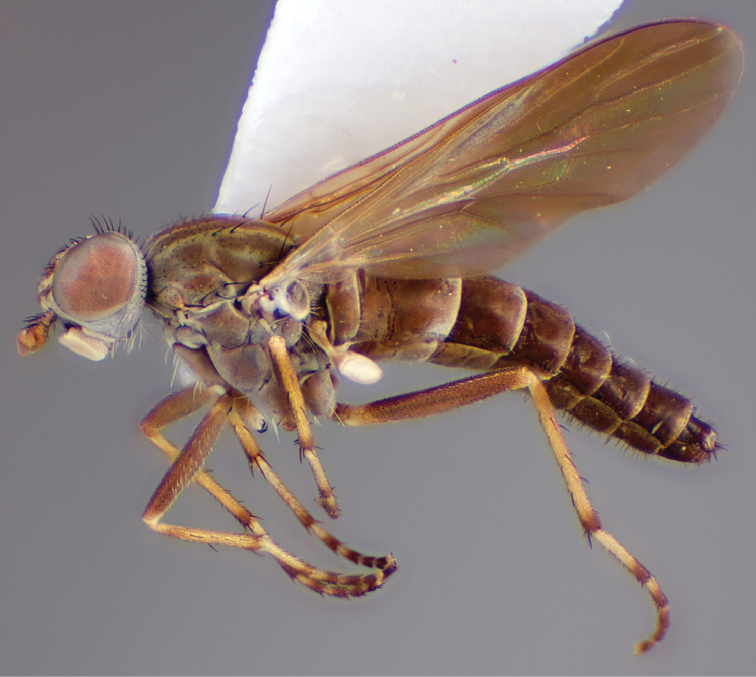
*Manestella obscura* sp. n., female, lateral view. Body length = 4.2 mm.

**Figure 40.**
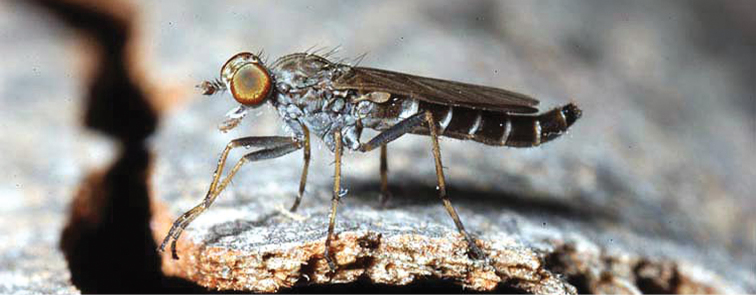
*Manestella obscura* sp. n., female, Queensland, Brisbane Forest Park. Body length = 4.5 mm. Photograph credit: Riley Nelson.

**Figure 41.**
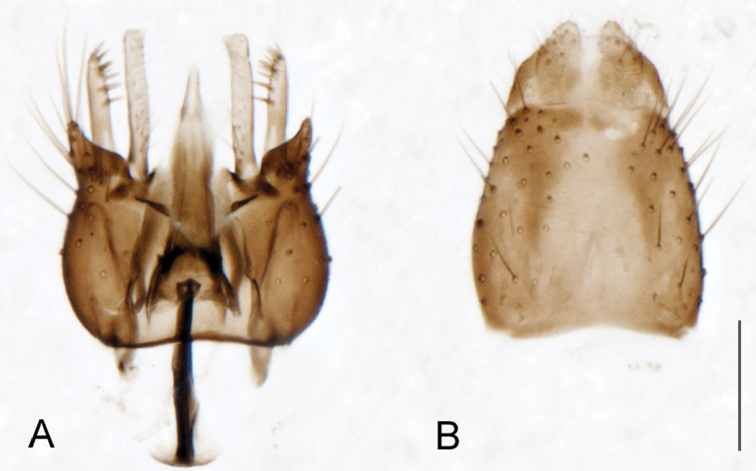
*Manestella obscura* sp. n., male genitalia **A** gonocoxites with aedeagus *in situ*, ventral view **B** epandrium. Scale line = 0.2 mm.

**Figure 42.**
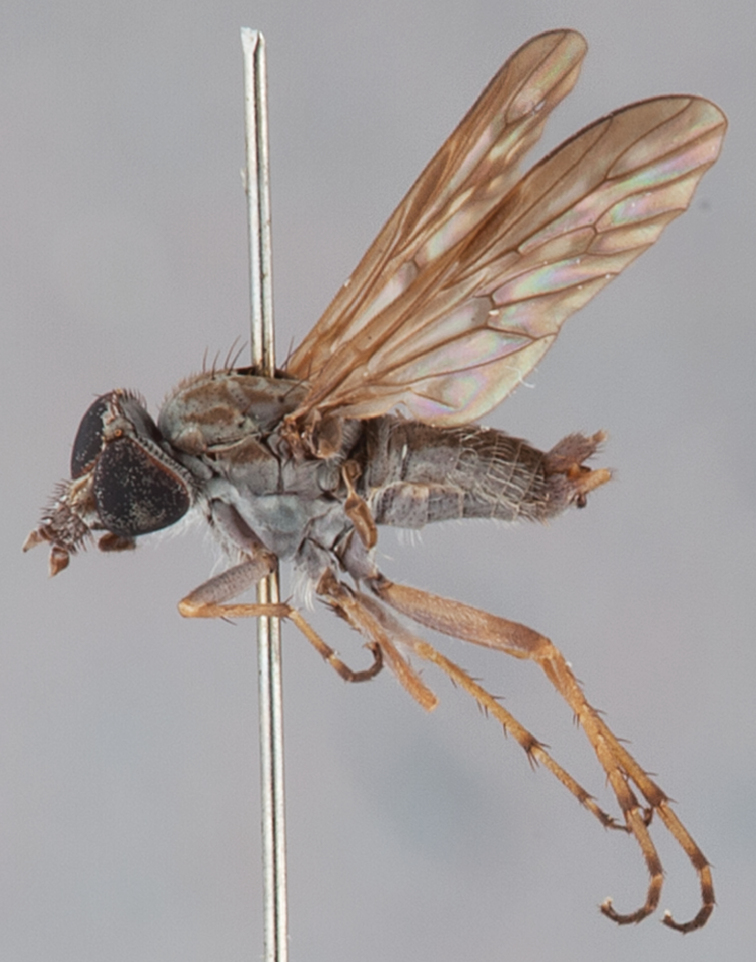
*Manestella ocellaris* sp. n., male, lateral view. Body length = 3.3 mm.

**Figure 43.**
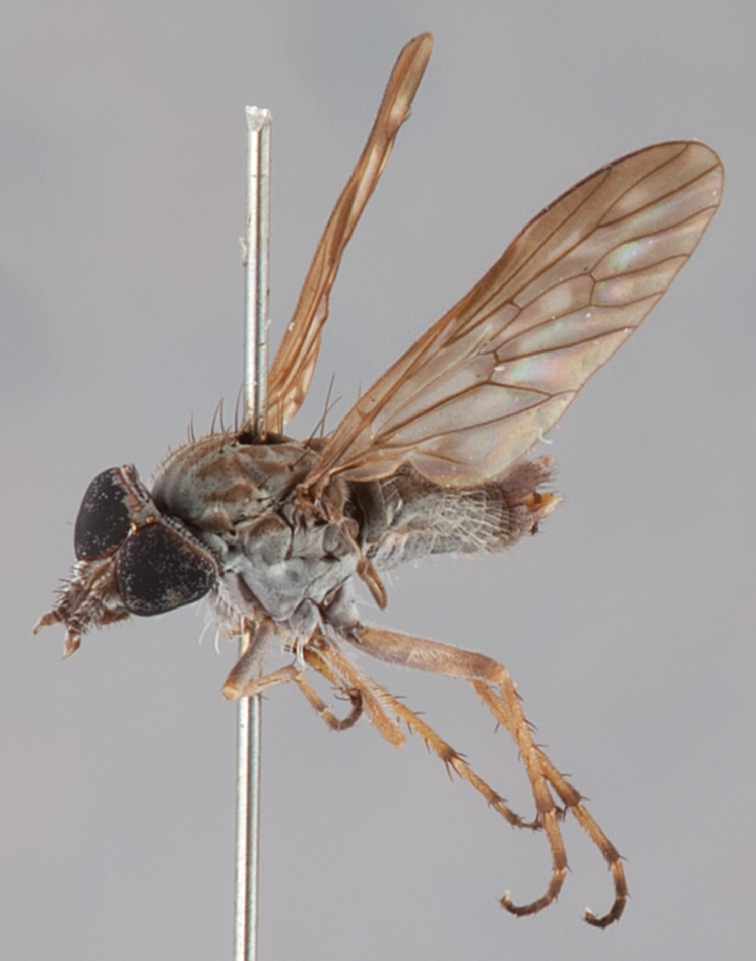
*Manestella ocellaris* sp. n., male, oblique view. Body length = 3.3 mm.

**Figure 44.**
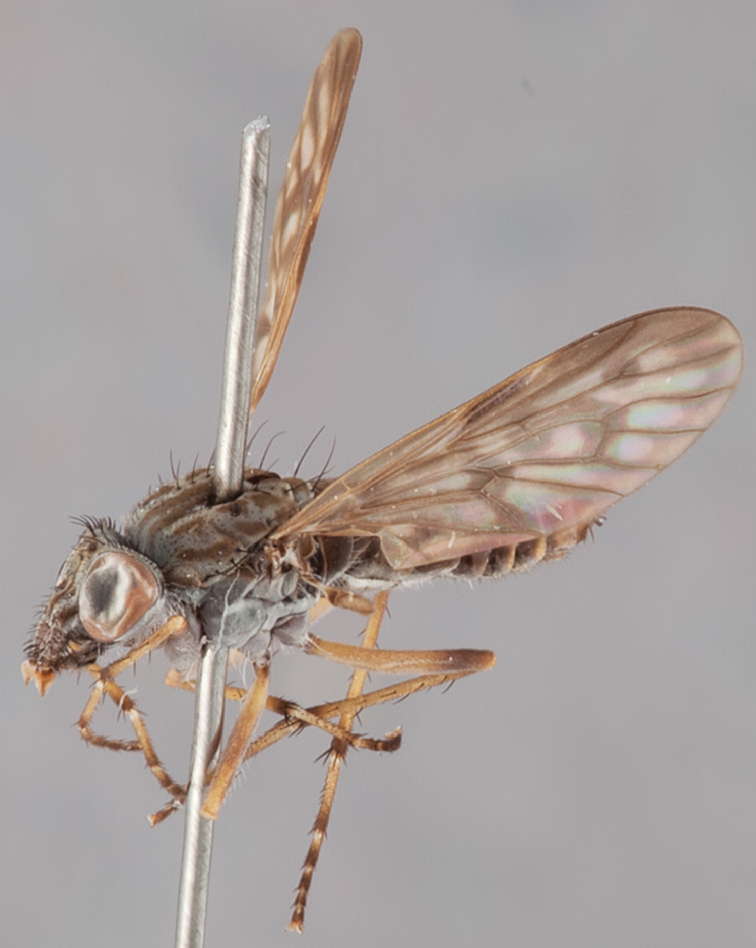
*Manestella ocellaris* sp. n., female, oblique view. Body length = 4.0 mm.

**Figure 45.**
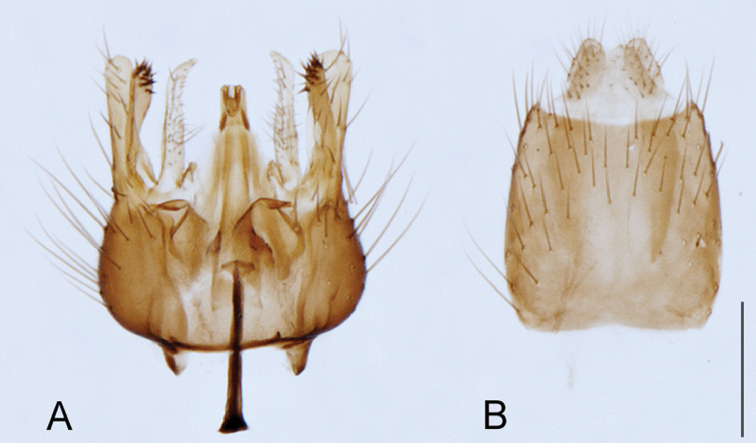
*Manestella ocellaris* sp. n., male genitalia **A** gonocoxites with aedeagus *in situ*, ventral view **B** epandrium. Scale line = 0.2 mm.

**Figure 46.**
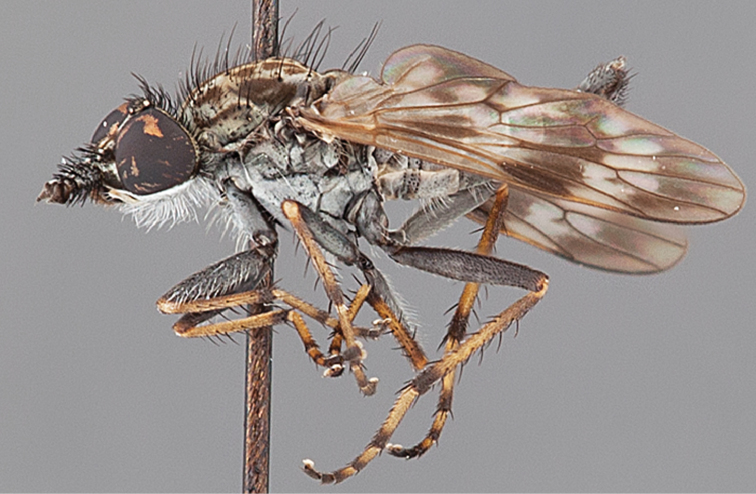
*Manestella persona* sp. n., male, lateral view. Body length = 4.0 mm.

**Figure 47.**
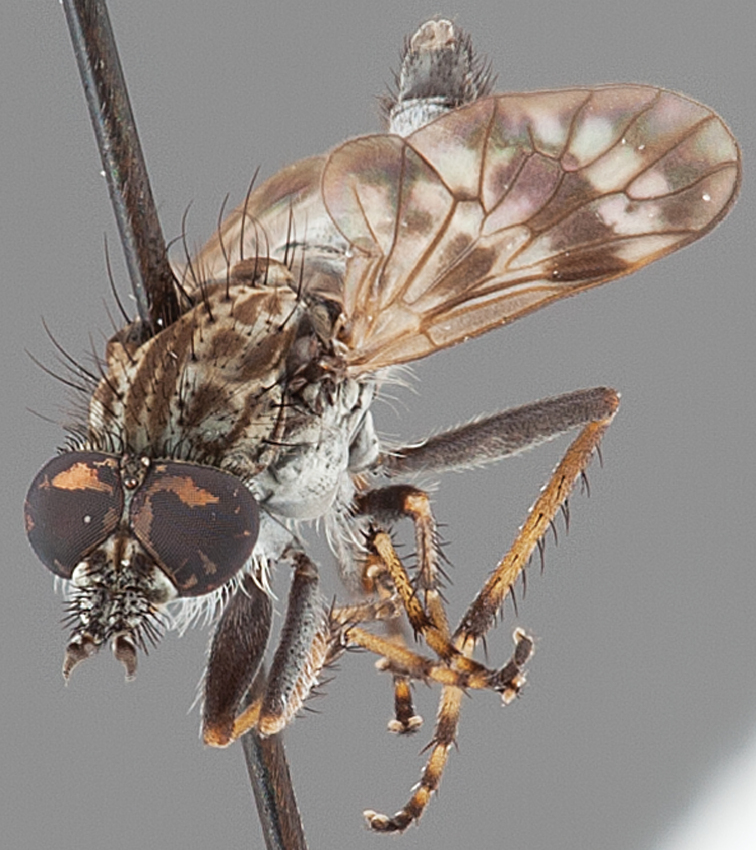
*Manestella persona* sp. n., male, oblique view. Body length = 4.0 mm.

**Figure 48.**
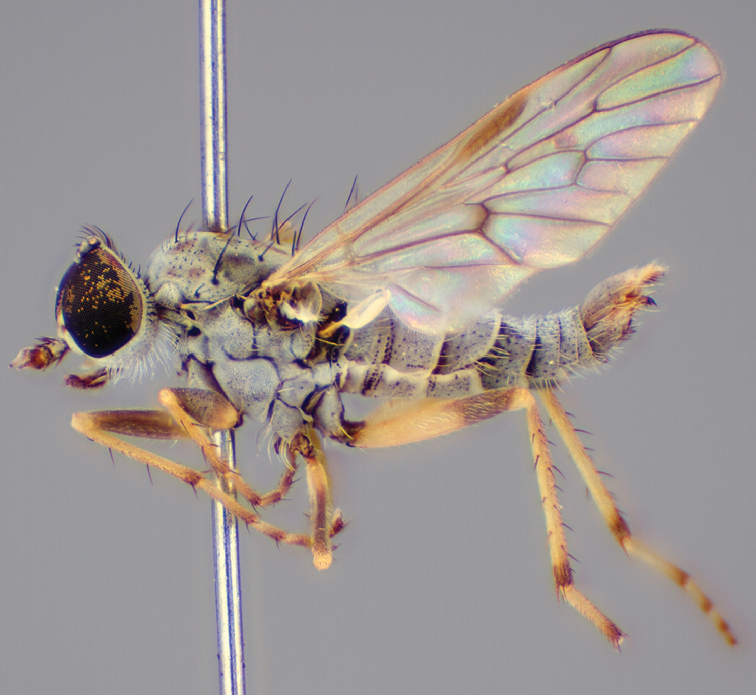
*Manestella poecilothorax* sp. n., male, lateral view. Body length = 3.5 mm.

**Figure 49.**
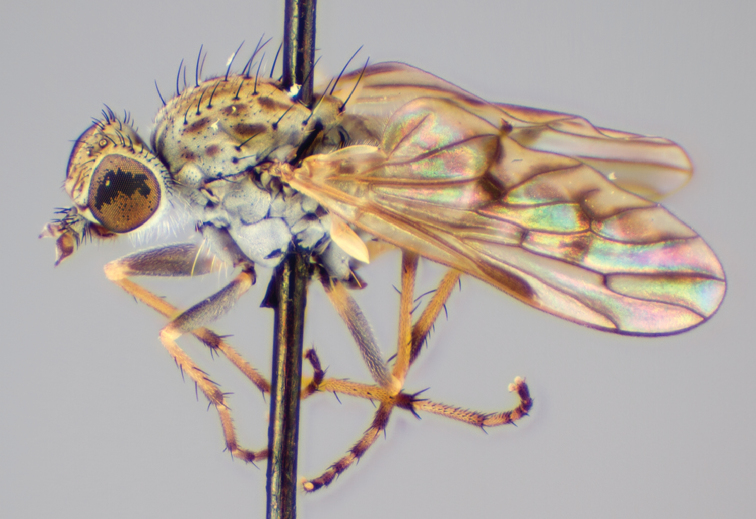
*Manestella poecilothorax* sp. n., female, lateral view. Body length = 4.5 mm.

**Figure 50.**
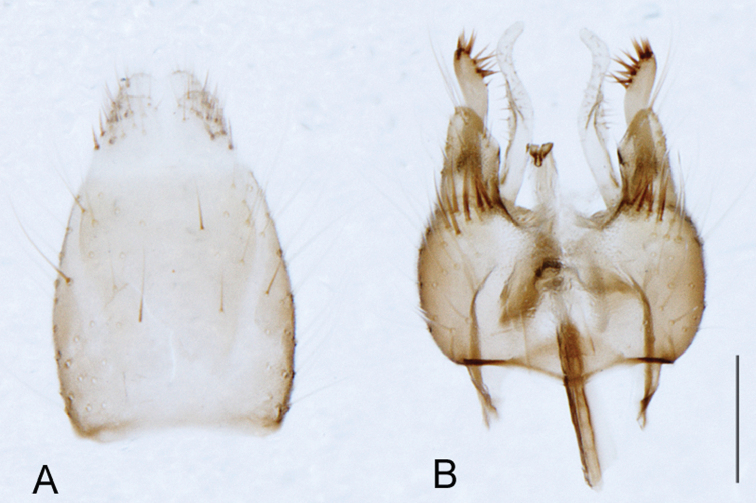
*Manestella poecilothorax* sp. n., male genitalia **A** epandrium **B** gonocoxites with aedeagus *in situ*, ventral view. Scale line = 0.2 mm.

**Figure 51.**
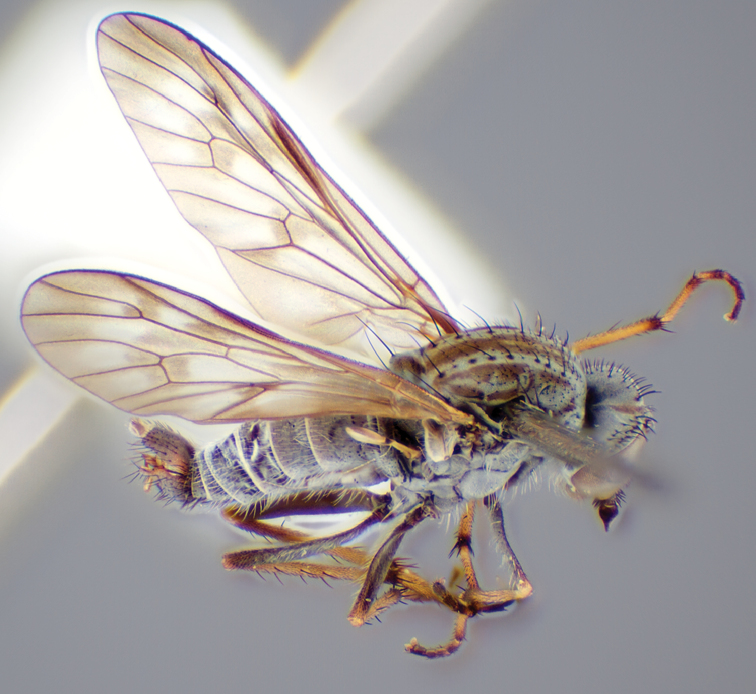
*Manestella tristriata* (Mann), male, lateral view. Body length = 3.5 mm.

**Figure 52.**
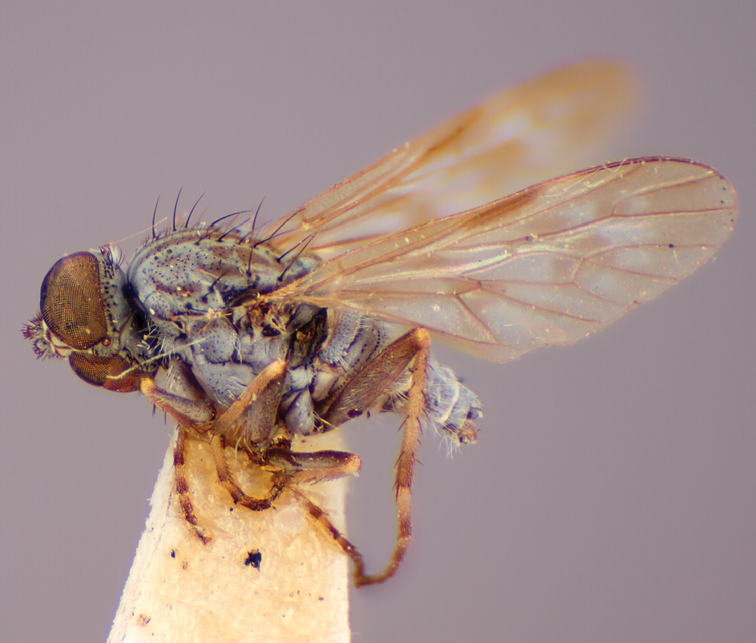
*Manestella tristriata* (Mann), holotype male, lateral view. Body length = 3.3 mm.

**Figure 53.**
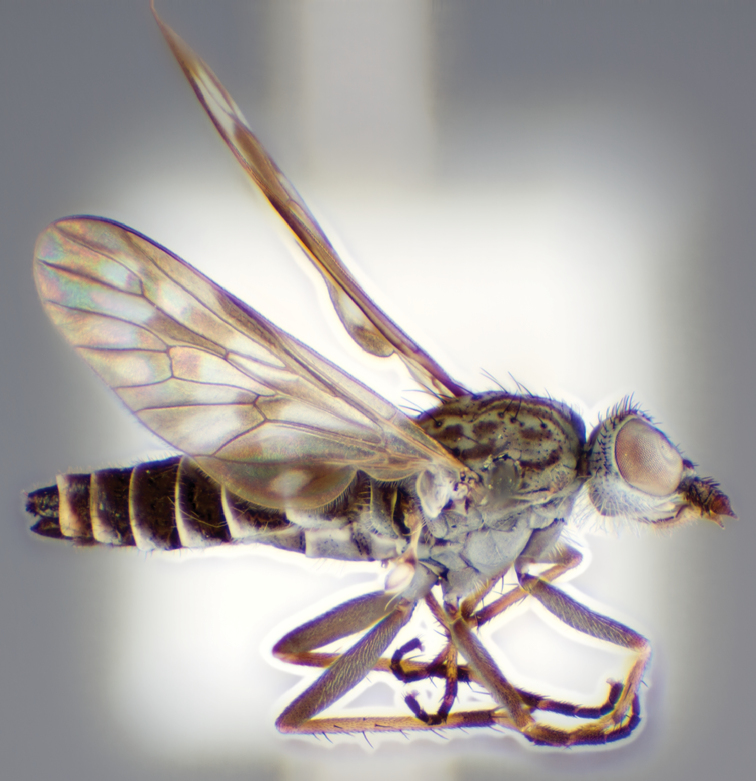
*Manestella tristriata* (Mann), female, lateral view. Body length = 4.5 mm.

**Figure 54.**
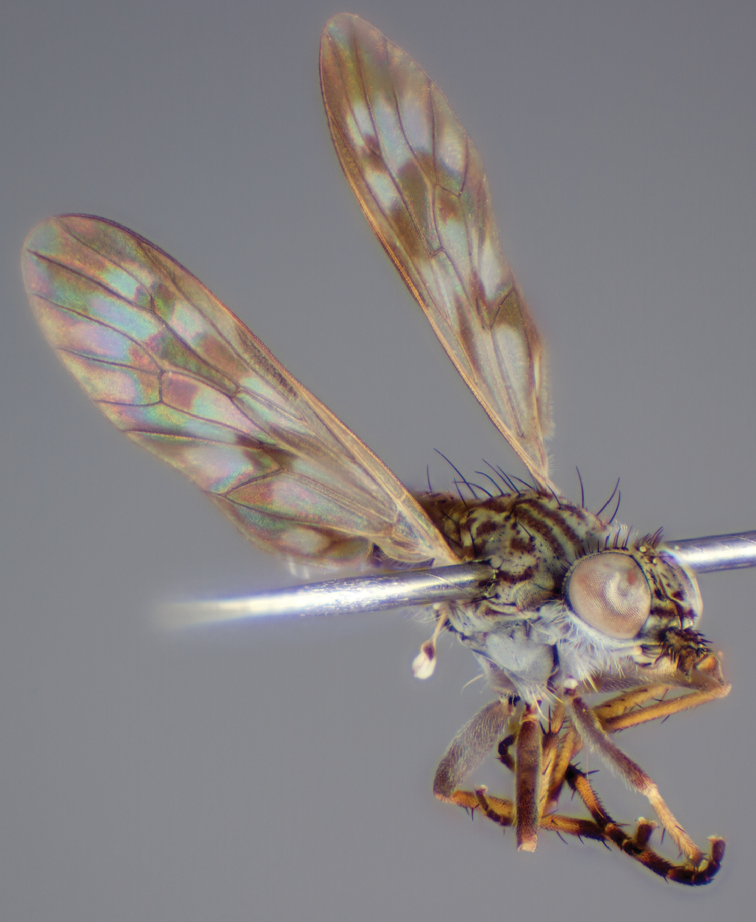
*Manestella tristriata* (Mann), female, oblique view. Body length = 4.5 mm.

**Figure 55.**
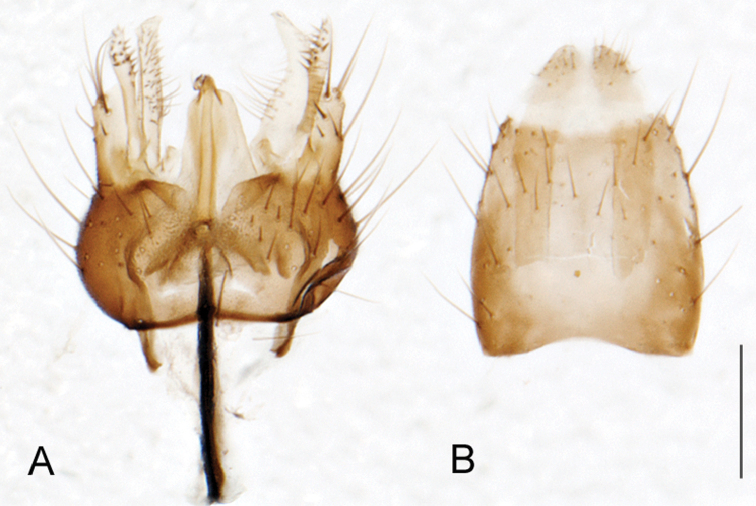
*Manestella tristriata* (Mann), male genitalia **A** gonocoxites with aedeagus *in situ*, ventral view **B** epandrium. Scale line = 0.2 mm.

**Figure 56.**
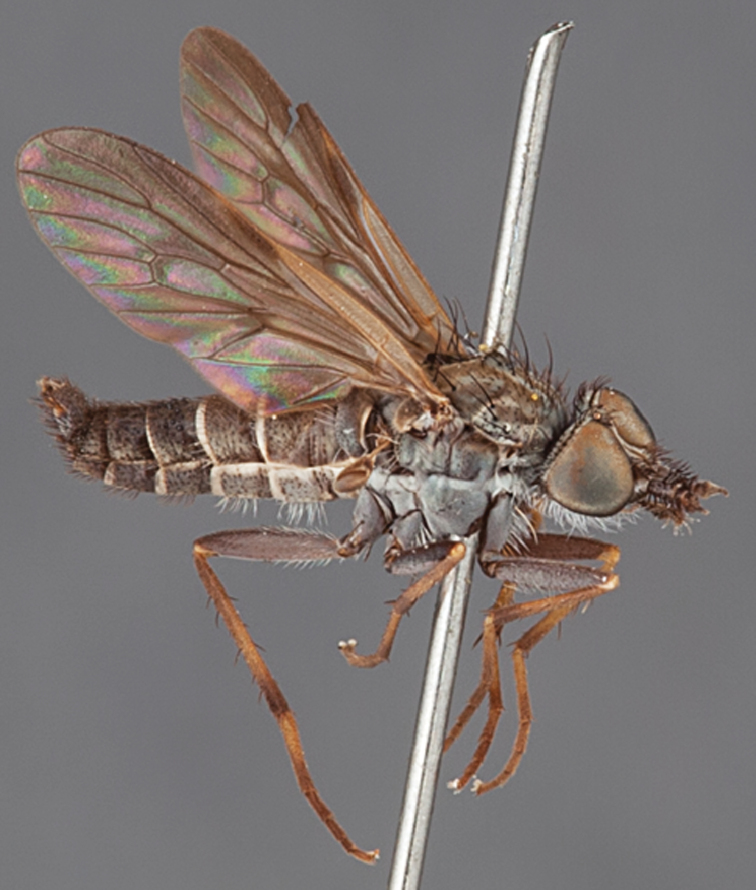
*Manestella umbrapennis* sp. n., male, lateral view. Body length = 3.0 mm.

**Figure 57.**
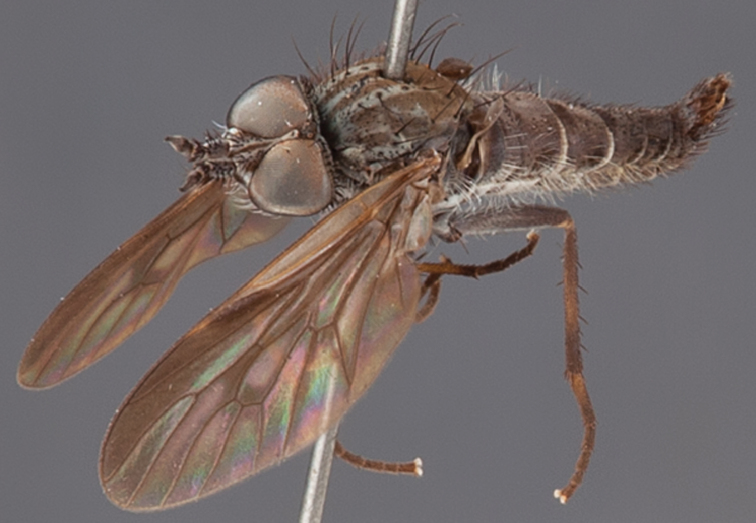
*Manestella umbrapennis* sp. n., male, oblique view. Body length = 3.0 mm.

**Figure 58.**
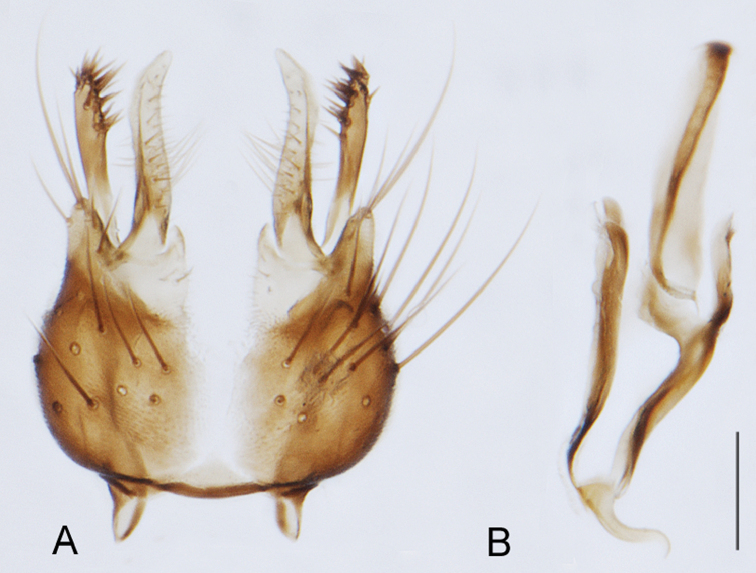
*Manestella umbrapennis* sp. n., male genitalia **A** gonocoxites, ventral view **B** aedeagus lateral view. Scale line = 0.2 mm.

**Figure 59.**
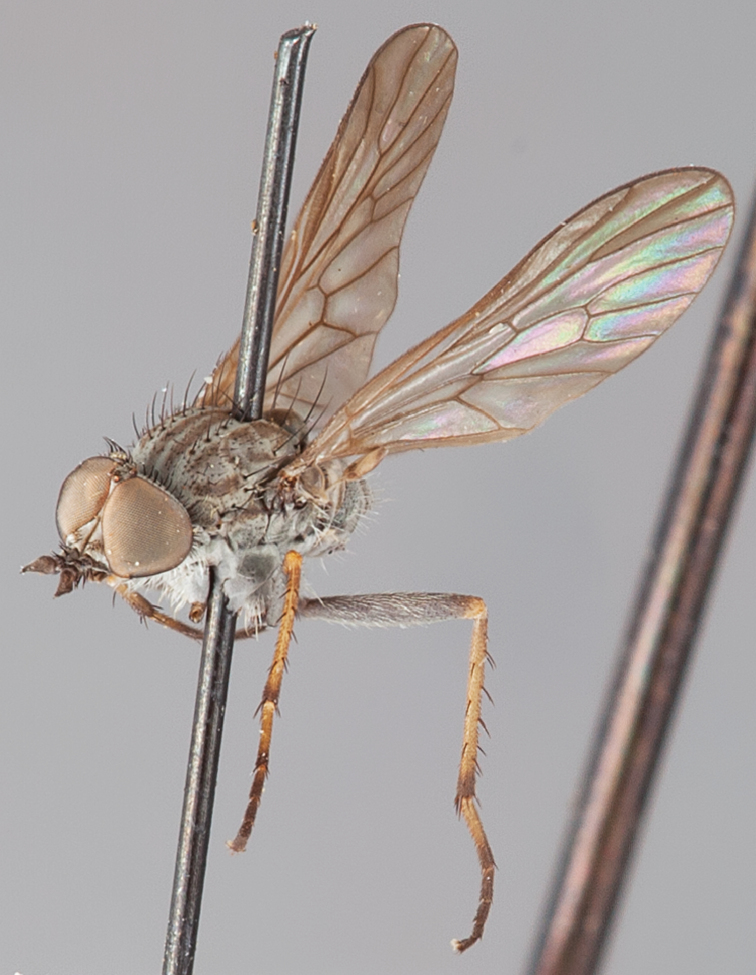
*Manestella vasta* sp. n., male, oblique view. Body length = 3.0 mm.

**Figure 60.**
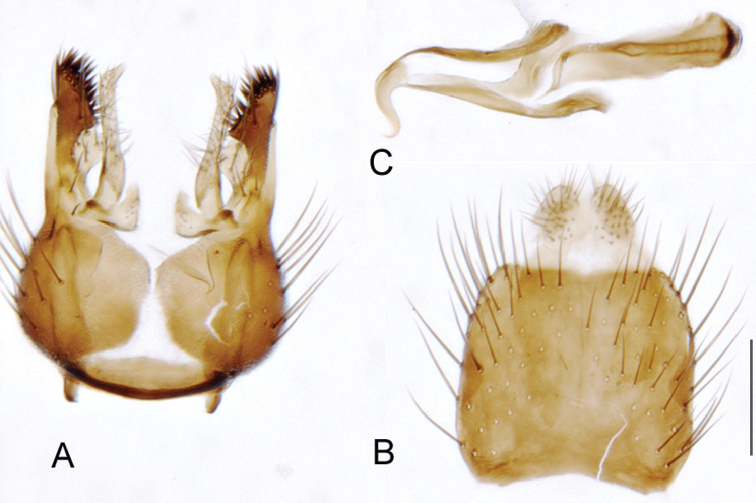
*Manestella vasta* sp. n., male genitalia **A** epandrium **B** gonocoxites, ventral view **C** aedeagus, lateral view. Scale line = 0.2 mm.

**Figure 62.**
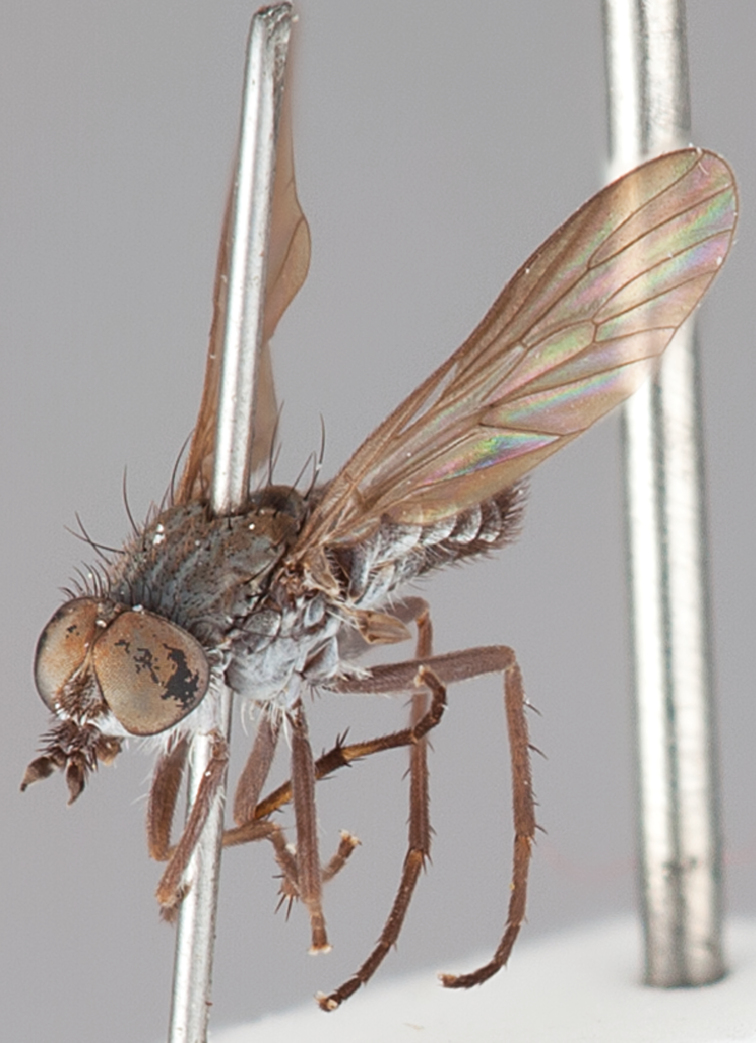
*Manestella vespera* sp. n., male, oblique view. Body length = 3.4 mm.

**Figure 63.**
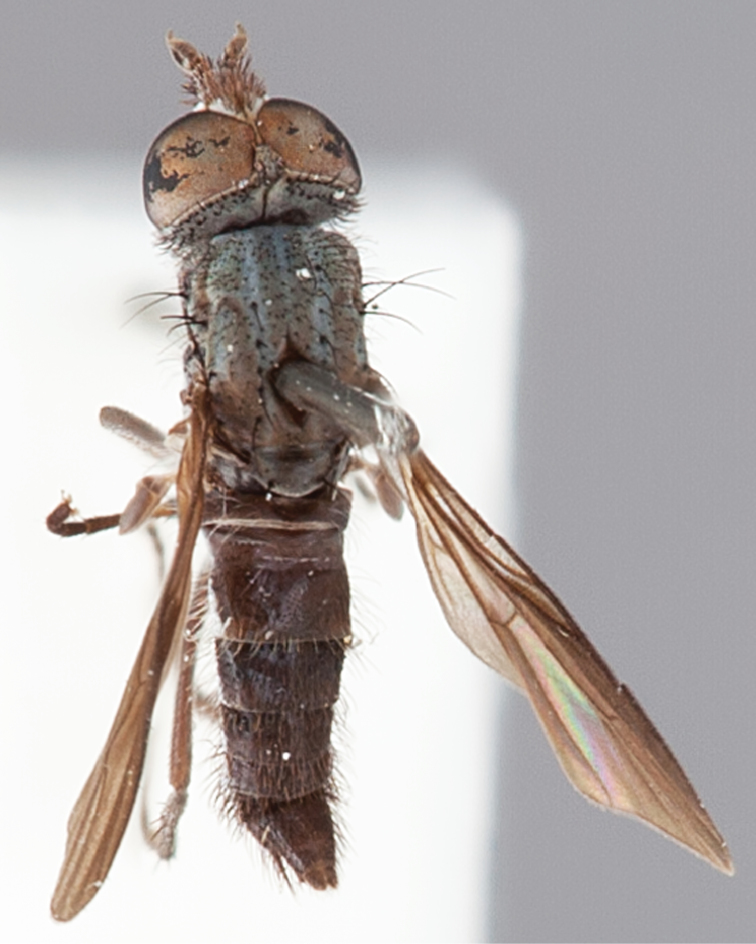
*Manestella vespera* sp. n., male, dorsal view. Body length = 3.4 mm.

**Figure 64.**
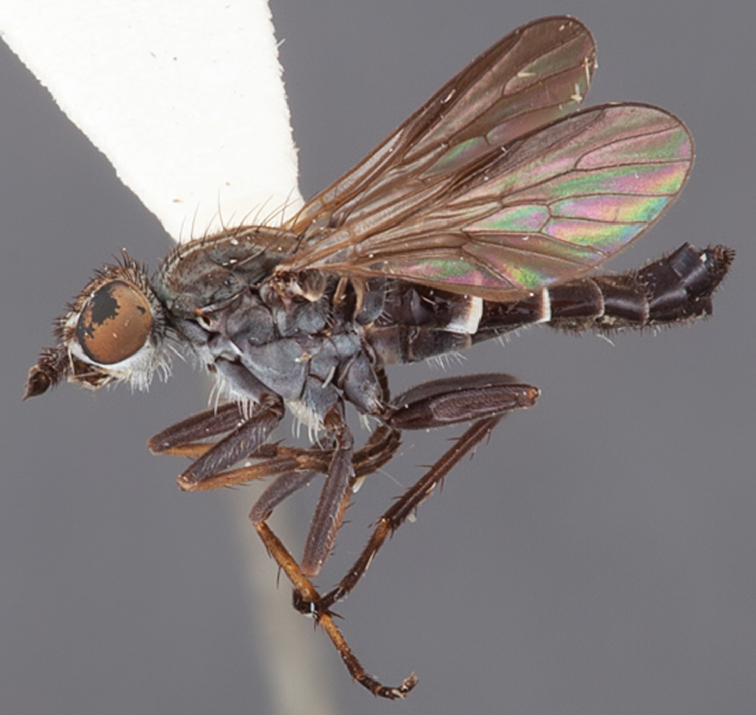
*Manestella vespera* sp. n., female, lateral view. Body length = 4.0 mm.

**Figure 65.**
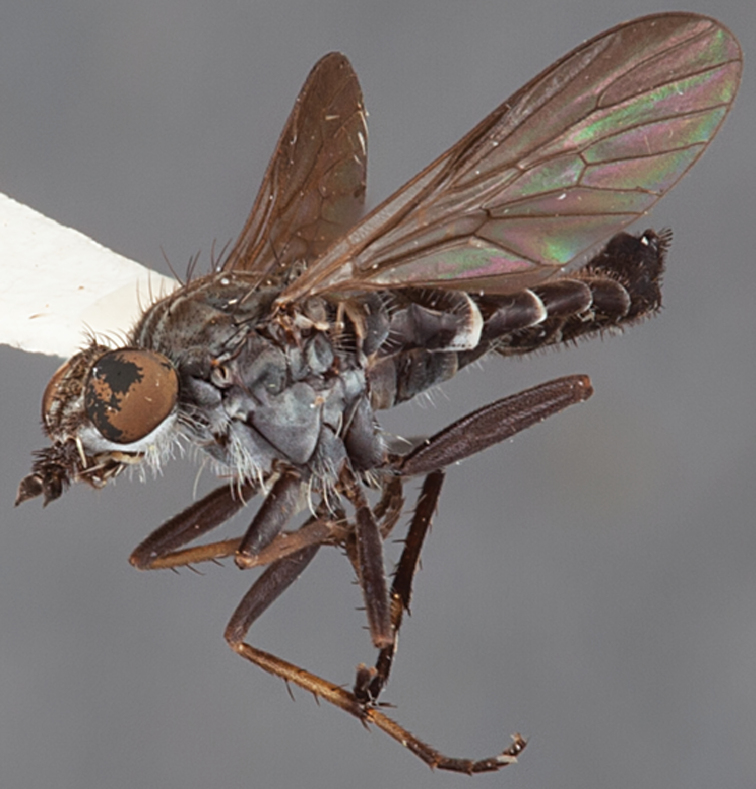
*Manestella vespera* sp. n., female, oblique view. Body length = 4.0 mm.

**Figure 66.**
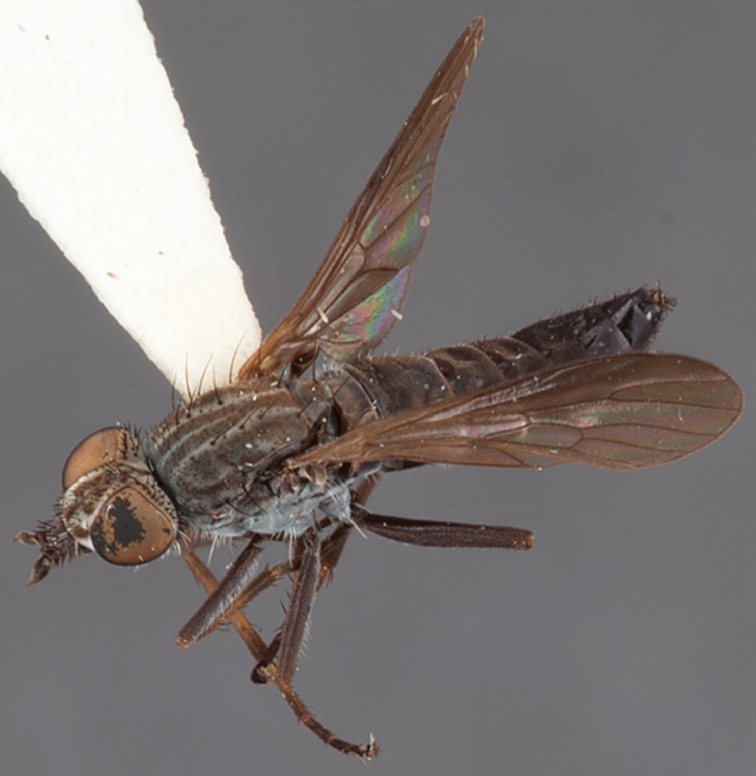
*Manestella vespera* sp. n., female, oblique view. Body length = 4.0 mm.

**Figure 67.**
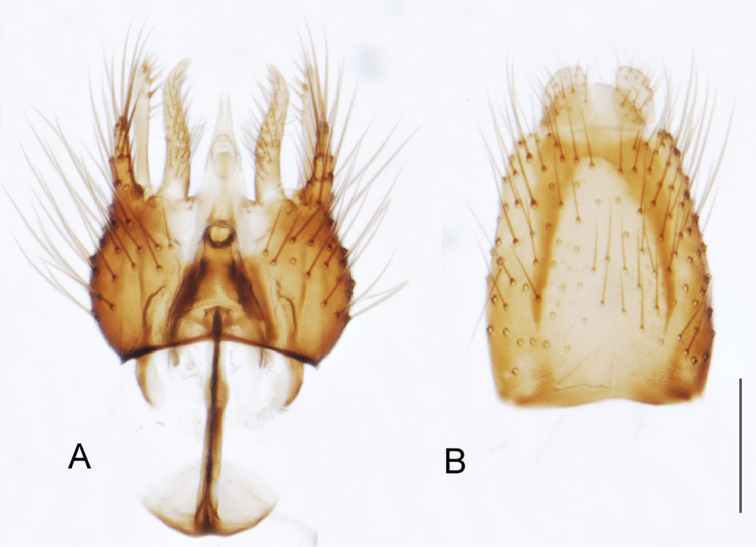
*Manestella vespera* sp. n., male genitalia **A** gonocoxites with aedeagus *in situ*, ventral view **B** epandrium. Scale line = 0.2 mm.

**Figure 69.**
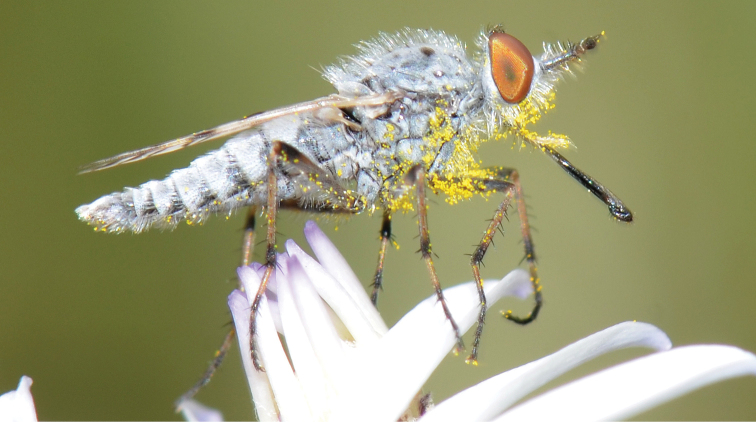
*Medomega averyi* sp. n., male, Western Australia, Stratton. Body length = 7.0 mm. Photograph credit: Fred and Jean Hort.

**Figure 70.**
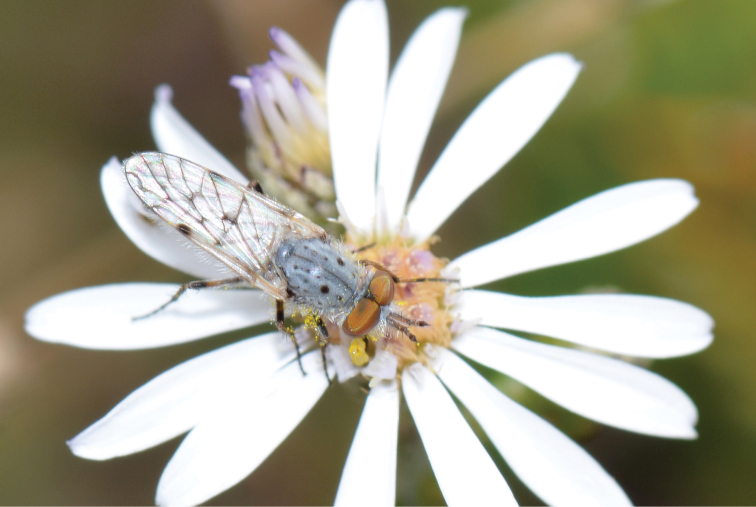
*Medomega averyi* sp. n., male, Western Australia, Stratton. Body length = 7.0 mm. Photograph credit: Fred and Jean Hort.

**Figure 73.**
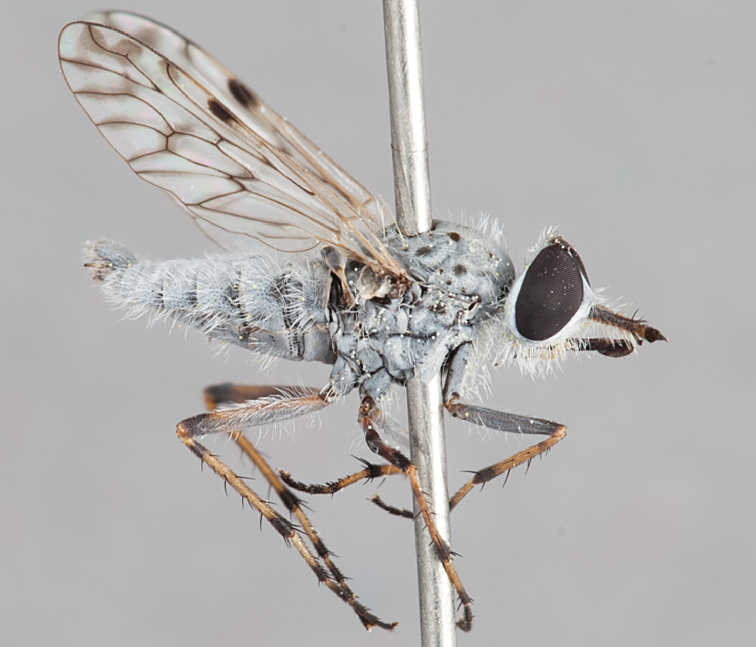
*Medomega averyi* sp. n., male, lateral view. Body length = 6.2 mm.

**Figure 74.**
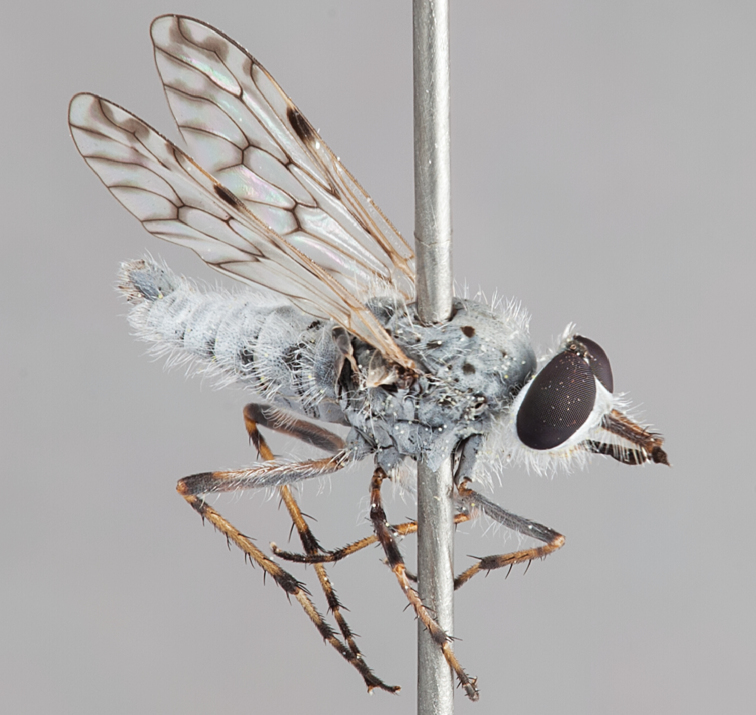
*Medomega averyi* sp. n., male, oblique view. Body length = 6.2 mm.

**Figure 75.**
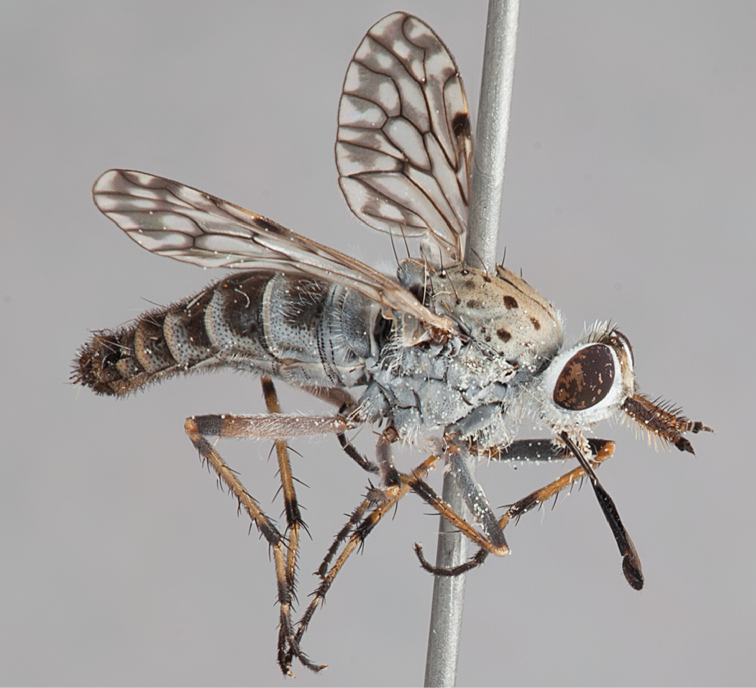
*Medomega averyi* sp. n., female, oblique view. Body length = 7.2 mm.

**Figure 76.**
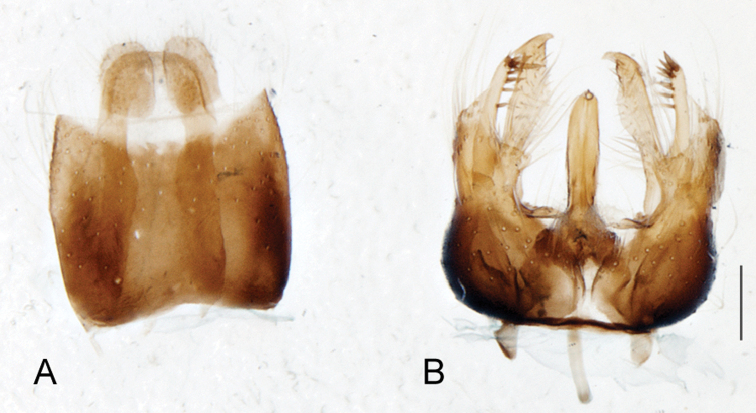
*Medomega averyi* sp. n., male genitalia **A** epandrium **B** gonocoxites with aedeagus *in situ*, ventral view. Scale line = 0.2 mm.

**Figure 77.**
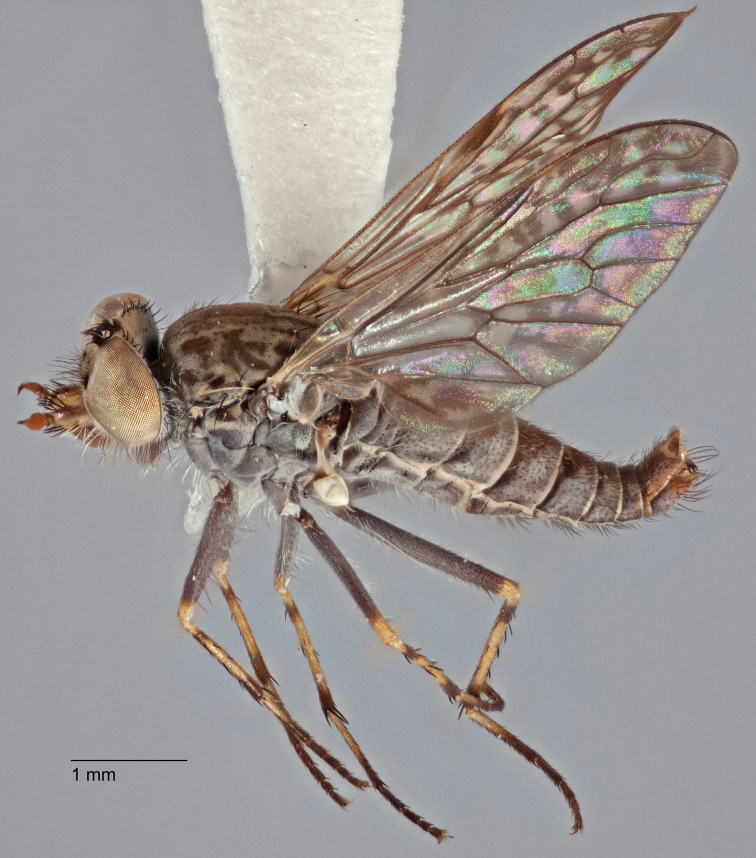
*Medomega bailmeup* sp. n., male, lateral view. Body length = 5.5 mm.

**Figure 78.**
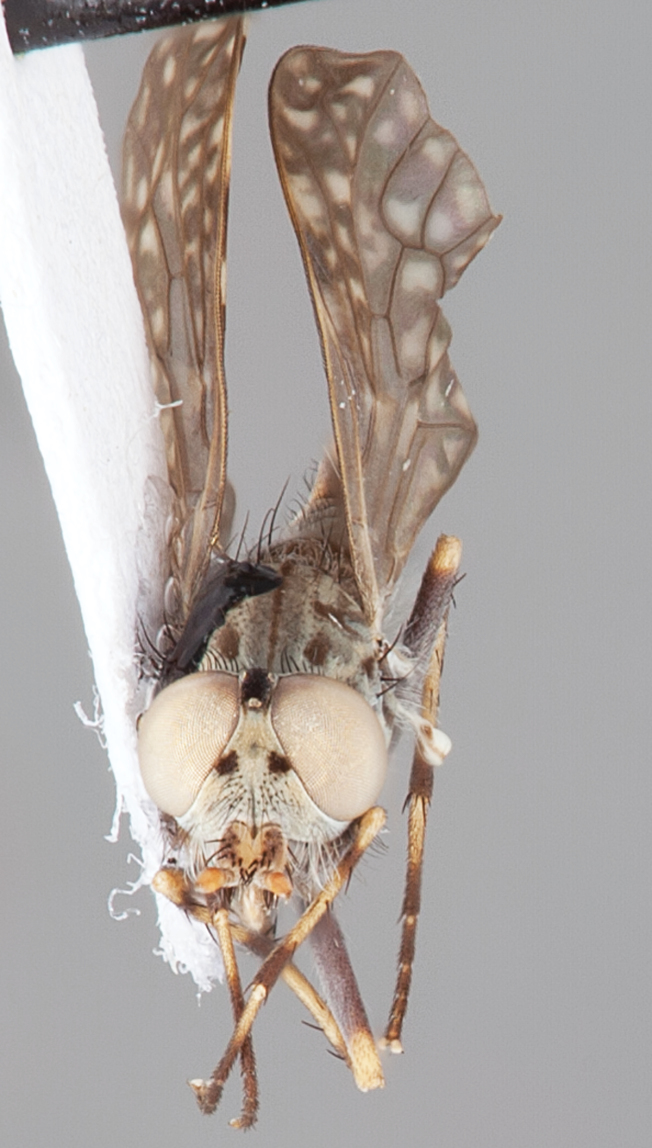
*Medomega bailmeup* sp. n., male, anterior view. Body length = 5.5 mm.

**Figure 79.**
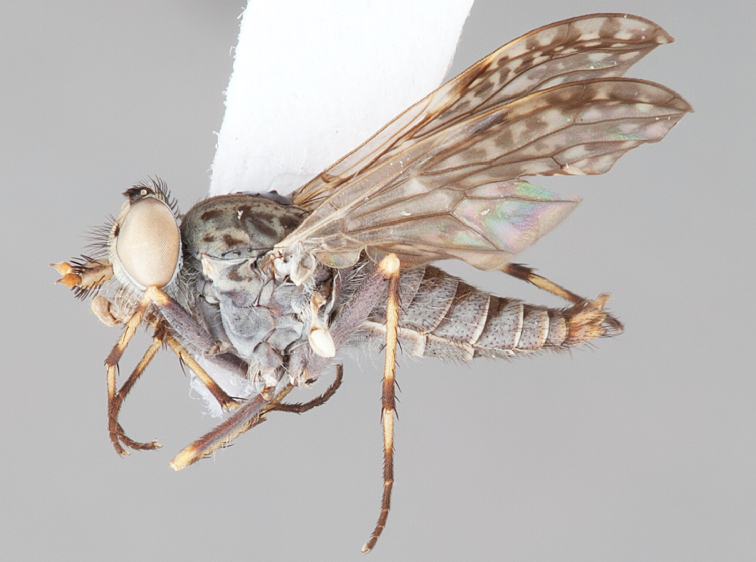
*Medomega bailmeup* sp. n., male, lateral view. Body length = 5.5 mm.

**Figure 80.**
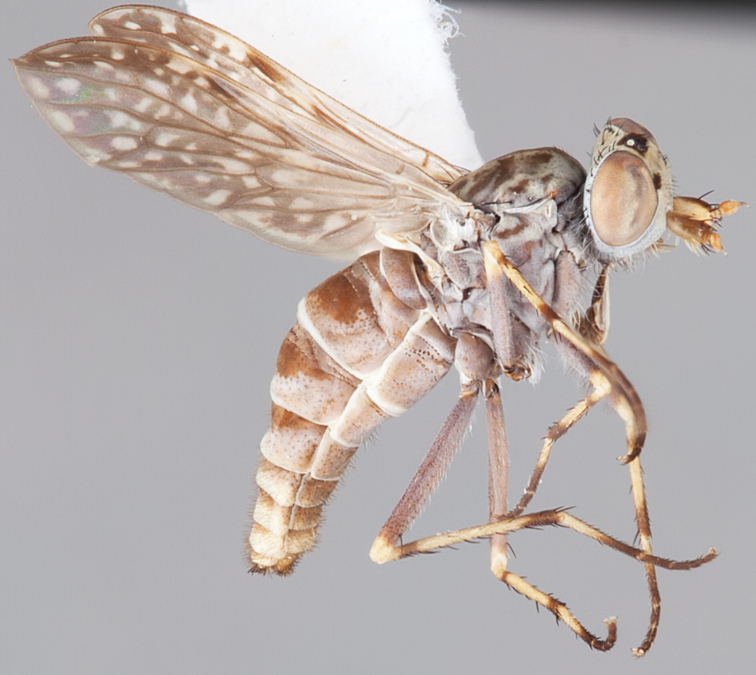
*Medomega bailmeup* sp. n., female, lateral view. Body length = 6.0 mm.

**Figure 81.**
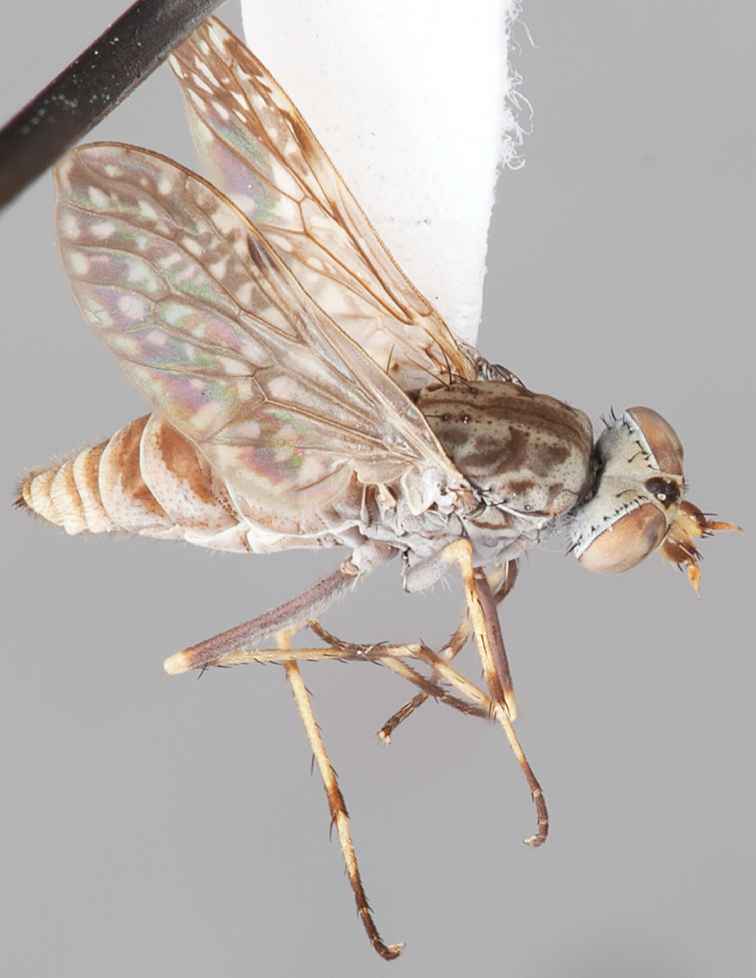
*Medomega bailmeup* sp. n., female, oblique view. Body length = 6.0 mm.

**Figure 82.**
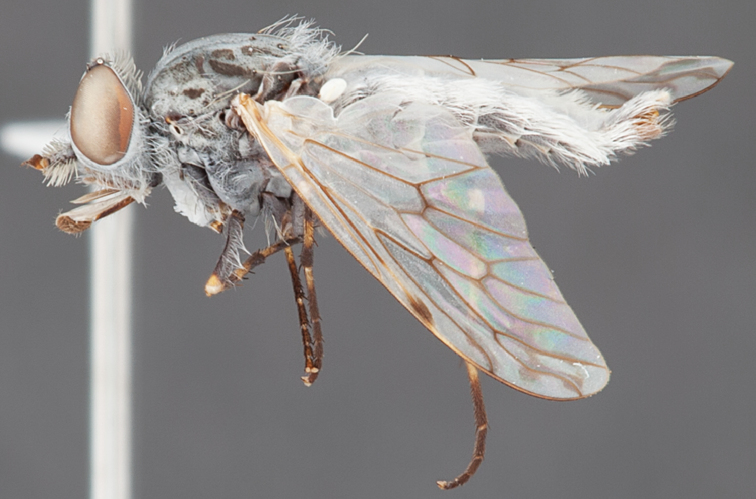
*Medomega chlamydos* sp. n., male, lateral view. Body length = 6.0 mm.

**Figure 83.**
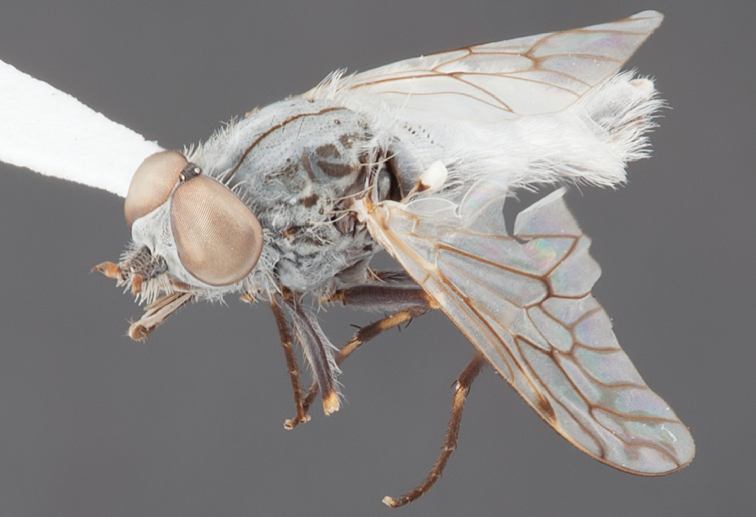
*Medomega chlamydos* sp. n., male, oblique view. Body length = 6.0 mm.

**Figure 84.**
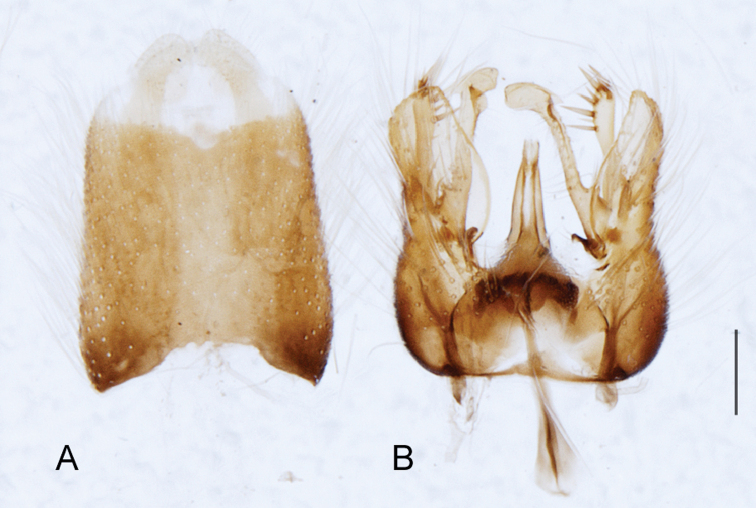
*Medomega chlamydos* sp. n., male genitalia **A** epandrium **B** gonocoxites with aedeagus *in situ*, ventral view. Scale line = 0.2 mm.

**Figure 85.**
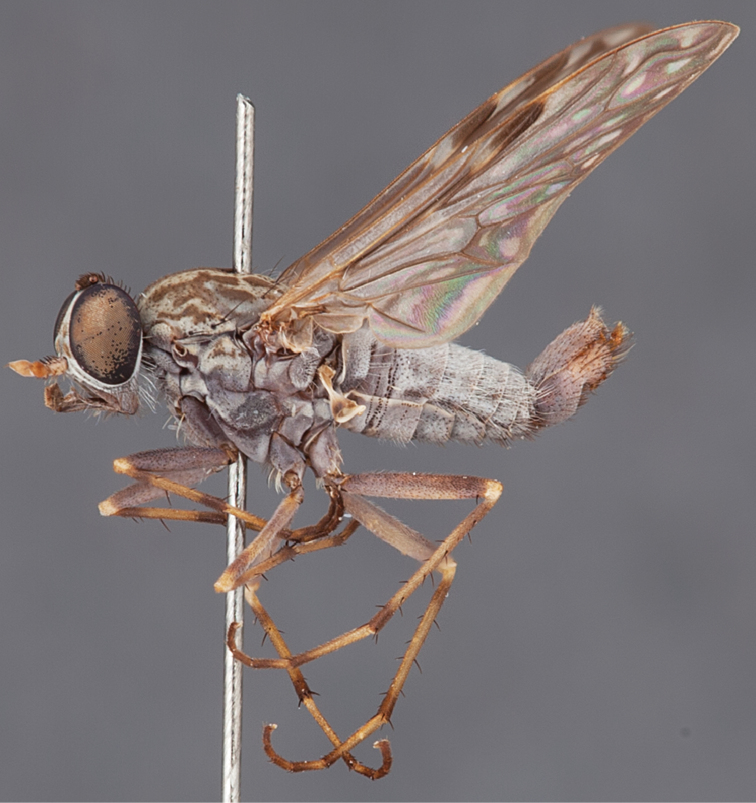
*Medomega danielsi* sp. n., male, lateral view. Body length = 6.0 mm.

**Figure 86.**
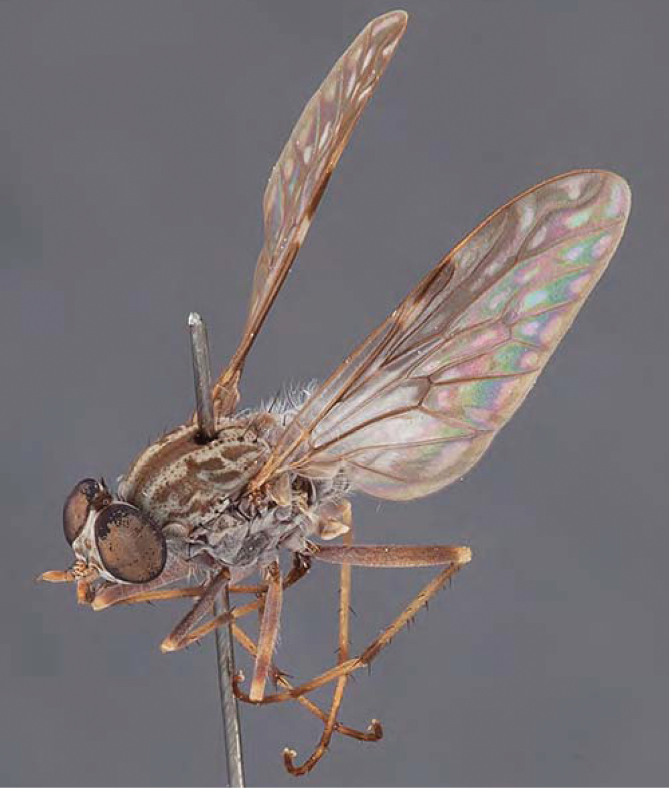
*Medomega danielsi* sp. n., male, oblique view. Body length = 6.0 mm.

**Figure 87.**
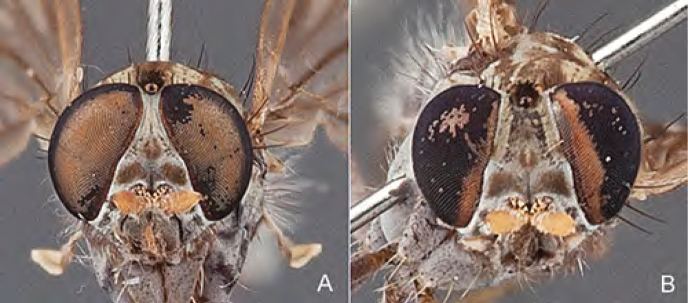
*Medomega danielsi* sp. n.: A, male head, anterior view; B, female head, anterior view.

**Figure 88.**
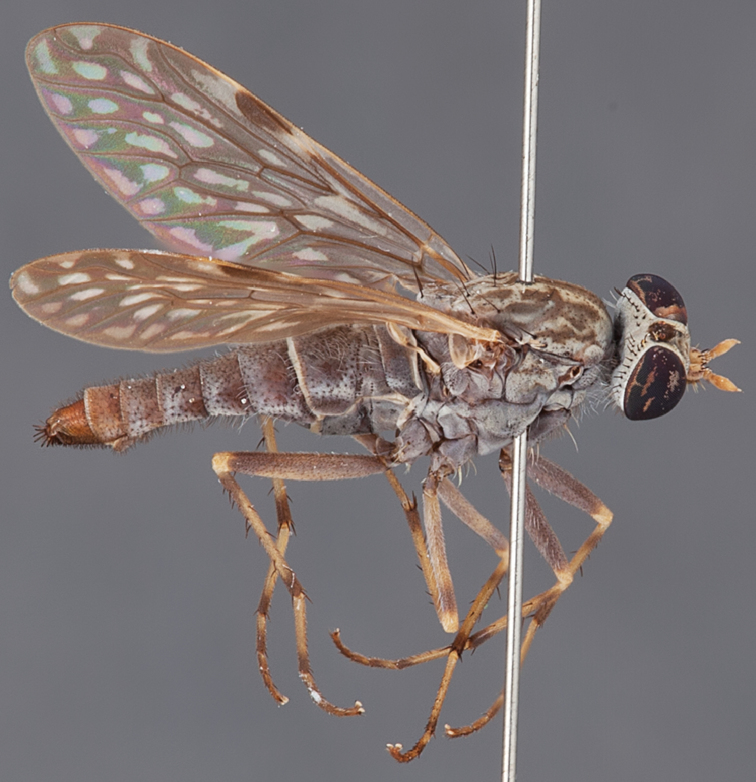
*Medomega danielsi* sp. n., female, lateral view. Body length = 6.5 mm.

**Figure 89.**
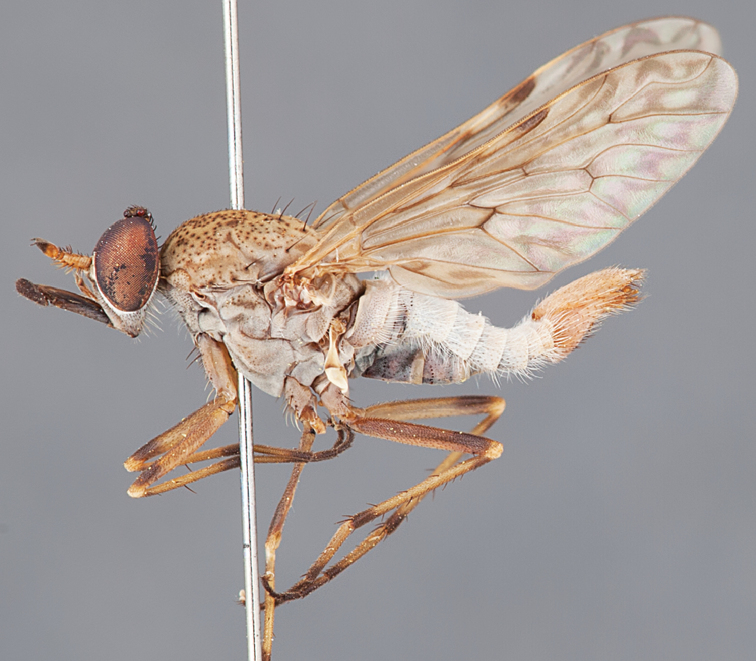
*Medomega gigasathe* sp. n., male, lateral view. Body length = 7.5 mm.

**Figure 90.**
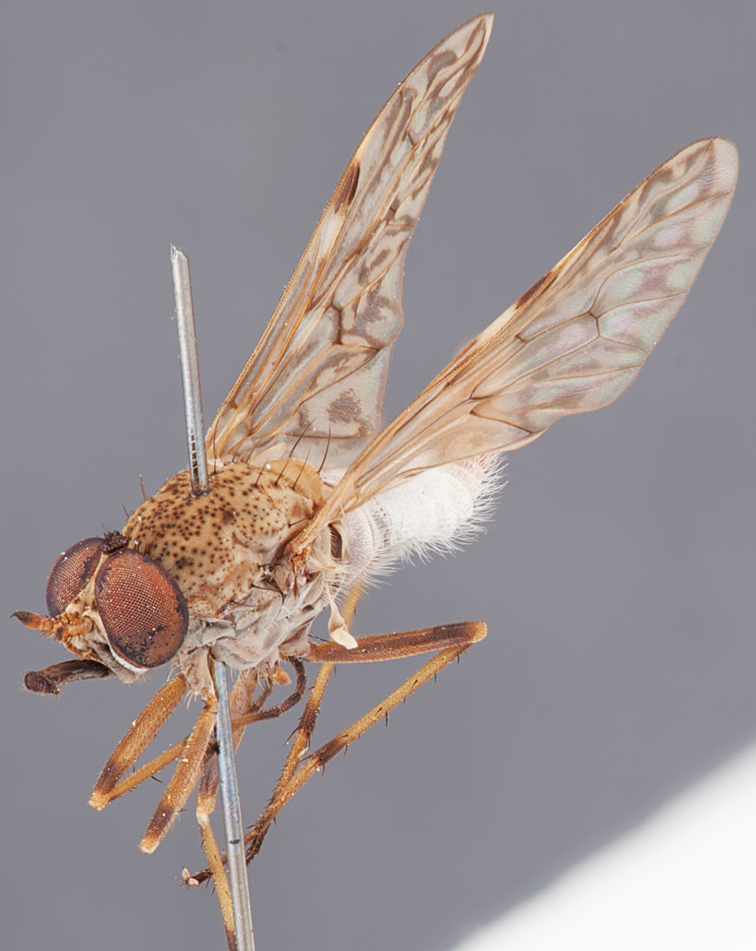
*Medomega gigasathe* sp. n., male, oblique view. Body length = 7.5 mm.

**Figure 91.**
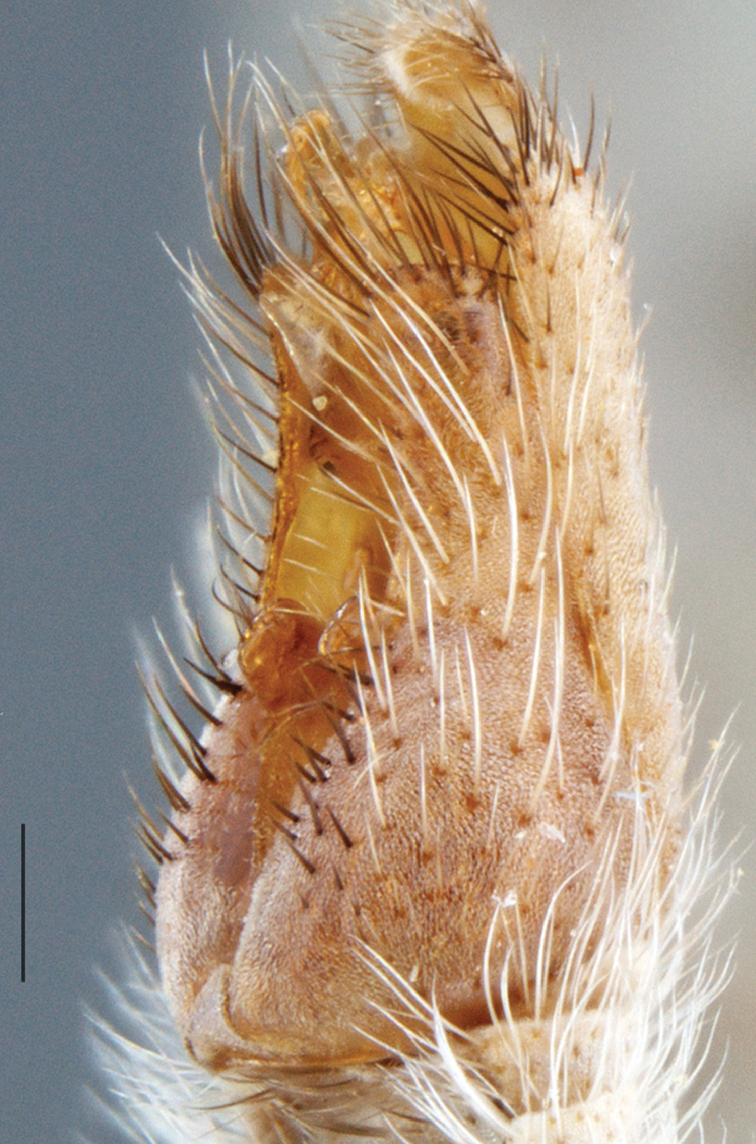
*Medomega gigasathe* sp. n., male genitalia, lateral view. Scale line = 0.2 mm.

**Figure 92.**
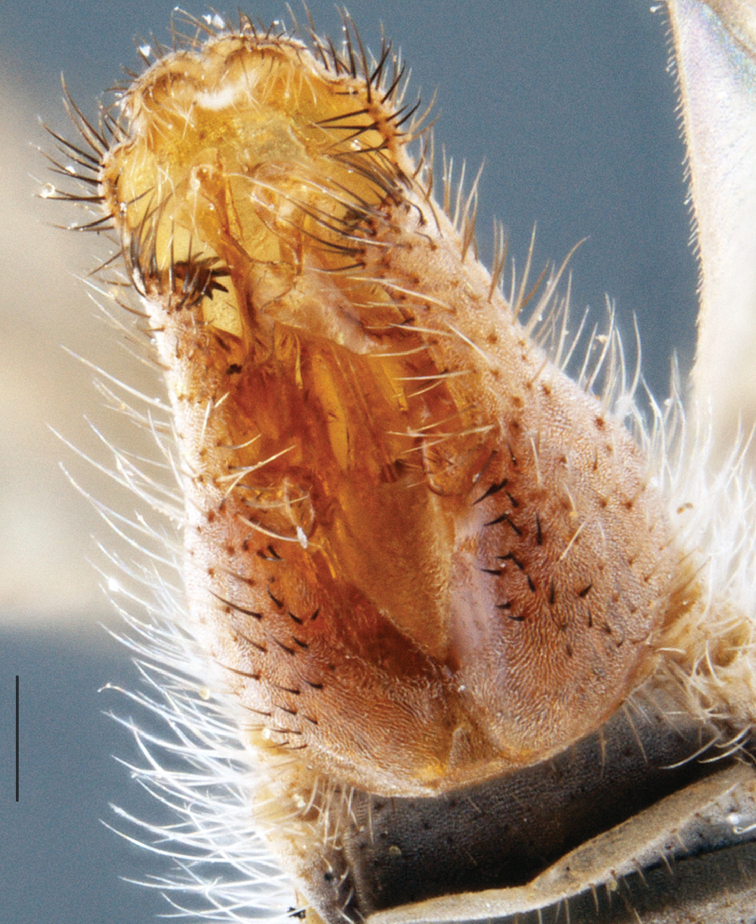
*Medomega gigasathe* sp. n., male genitalia, ventral view. Scale line = 0.2 mm.

**Figure 94.**
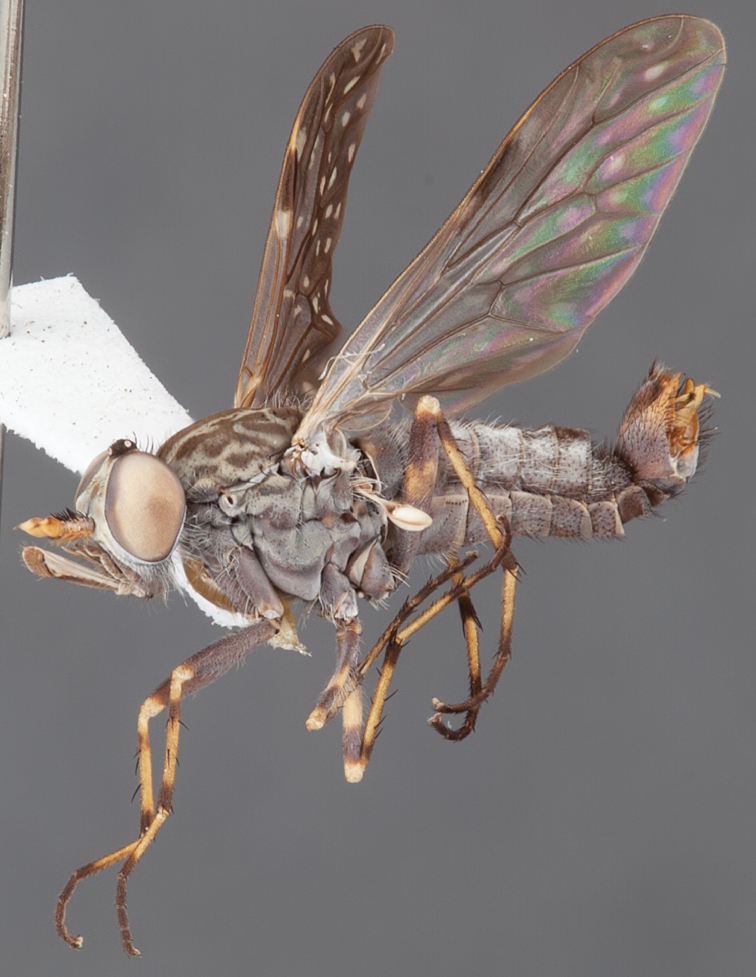
*Medomega nebrias* sp. n., male, oblique view. Body length = 7.0 mm.

**Figure 95.**
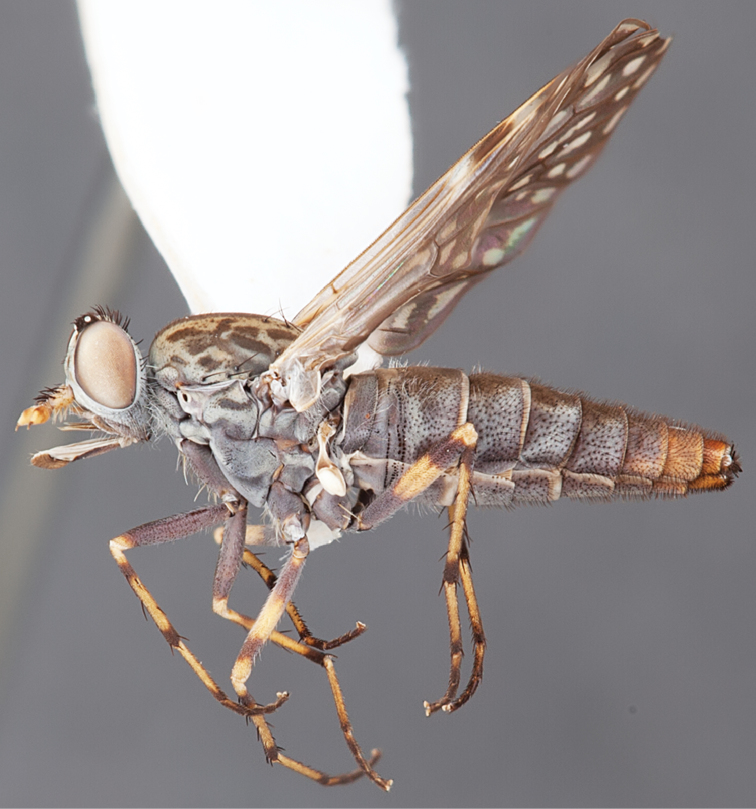
*Medomega nebrias* sp. n., female, lateral view. Body length = 7.5 mm.

**Figure 96.**
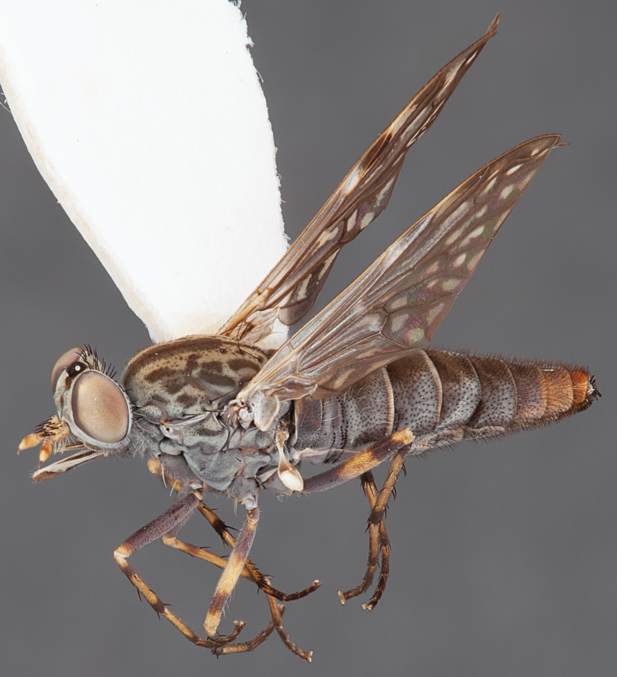
*Medomega nebrias* sp. n., female, oblique view. Body length = 7.5 mm.

**Figure 97.**
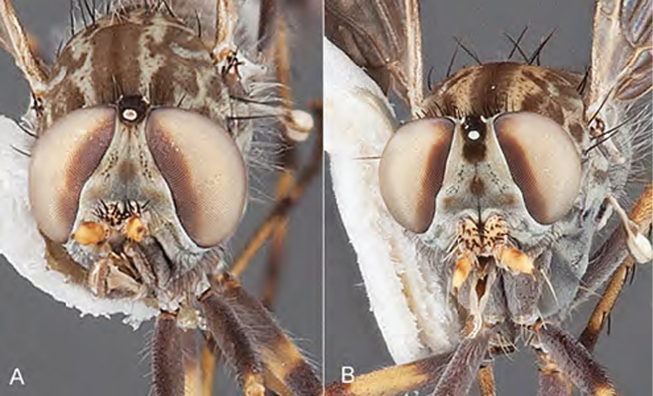
*Medomega nebrias* sp. n.: **A** male head, anterior view **B** female head, anterior view.

**Figure 98.**
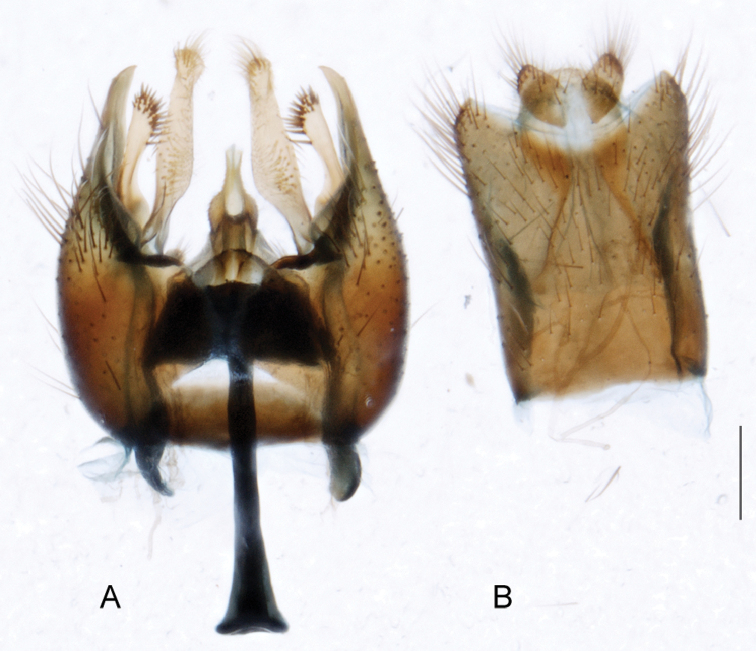
*Medomega nebrias* sp. n., male genitalia **A** epandrium **B** gonocoxites with aedeagus *in situ*, ventral view. Scale line = 0.2 mm.
